# Transposable elements are associated with genome-specific gene expression in bread wheat

**DOI:** 10.3389/fpls.2022.1072232

**Published:** 2023-01-12

**Authors:** Inbar Bariah, Liel Gribun, Khalil Kashkush

**Affiliations:** Department of Life Sciences, Ben-Gurion University, Beer-Sheva, Israel

**Keywords:** transposable elements, wheat, genome evolution, allopolyploidy, genome-specific, *Triticum aestivum*, gene expression, copy number variation

## Abstract

**Introduction:**

Recent studies in wheat emphasized the importance of TEs, which occupy ~85% of the wheat genome, as a major source of intraspecific genetic variation due to their recent activity and involvement in genomic rearrangements. The contribution of TEs to structural and functional variations in bread wheat genes is not fully understood.

**Methods:**

Here, publicly available RNA-Seq databases of bread wheat were integrated to identify TE insertions within gene bodies (exons\ introns) and assess the impact of TE insertions on gene expression variations of homoeologs gene groups. Overall, 70,818 homoeologs genes were analyzed: 55,170 genes appeared in each one of the three subgenomes (termed ABD), named triads; 12,640 genes appeared in two of the three subgenomes (in A and B only, termed AB; or in A and D only, termed AD; or in B and D only, termed BD);, named dyads; and 3,008 genes underwent duplication in one of the three subgenomes (two copies in: subgenome A, termed AABD; subgenome B, termed ABBD; or subgenome D, termed ABDD), named tetrads.

**Results:**

To this end, we found that ~36% of the 70,818 genes contained at least one TE insertion within the gene body, mostly in triads. Analysis of 14,258 triads revealed that the presence of TE insertion in at least one of the triad genes (7,439 triads) was associated with balanced expression (similar expression levels) between the homoeolog genes. TE insertions within the exon or in the untranslated regions (UTRs) of one or more of the homoeologs in a triad were significantly associated with homoeolog expression bias. Furthermore, we found a statistically significant correlation between the presence\absence of TEs insertions belonging to six TE superfamilies and 17 TE subfamilies and the suppression of a single homoeolog gene. A significant association was observed between the presence of TE insertions from specific superfamilies and the expression of genes that are associated with biotic and abiotic stress responses.

**Conclusion:**

Our data strongly indicate that TEs might play a prominent role in controlling gene expression in a genome-specific manner in bread wheat.

## 1 Introduction

Transposable elements (TEs) are a major component of plant genomes (Mhiri et al., 2022), e.g., they account for ~85% of the bread wheat genome (Appels et al., 2018; Wicker et al., 2018). Once thought of as “junk DNA” and “parasites”, today, a growing body of evidence reveals that TEs have a prominent role in genome evolution (Avni et al., 2017; Bourque et al., 2018; Dubin et al., 2018). TEs are capable of moving and increasing their copy number within the host genome mainly through copy and paste (Class I, retrotransposons) or cut and paste (Class II) mechanisms (Wicker et al., 2007). The highly repetitive nature, high abundance, and activity of TEs might trigger massive structural genomic rearrangements (Gray, 2000; Bourque et al., 2018; Krasileva, 2019). They are considered a great source for genetic variation, mainly creating new alleles by transposing within gene bodies (Bourque et al., 2018; Dubin et al., 2018).

The high abundance of TEs near and within plant genes might impact the function of those genes by influencing both transcriptional and post-transcriptional levels and lead to the creation of novel transcripts (Schrader and Schmitz, 2019; Bariah et al., 2020; Zhang et al., 2021). The mere presence of TE, adjacent or within the transcribed region, might result in gene downregulation or silencing due to epigenetic modifications or interfering with enhancers or regulatory promoter elements (Dubin et al., 2018). Furthermore, TEs contain regulatory sequences such as promoters, transcription factors binding sits, and target sites for post-transcriptional degradation, which might affect adjacent gene expression or even modulate gene expression through complex transcriptional regulatory networks (Bourque et al., 2018; Dubin et al., 2018; Qiu and Köhler, 2020; Zhang et al., 2021). Additionally, the insertion of TE into a gene body might result in the creation of new isoforms through exonization, truncation, alternative splicing, or even by the domestication of TE-derived coding sequences into host genes, potentially altering the gene function (Keidar et al., 2018; Poretti et al., 2019; Crescente et al., 2022).

Wheat (*Triticum*- *Aegilops* group) is among the world’s most widely grown crops, providing a significant portion of daily human caloric intake (Shewry and Hey, 2015; Levy and Feldman, 2022). The most widely grown bread wheat, *Triticum aestivum*, is a relatively new polyploid species that has been generated by two subsequent allopolyploidization events between members of two closely related genera, *Triticum* and *Aegilops* (Avni et al., 2017; Appels et al., 2018; Levy and Feldman, 2022). Allopolyploidization is the only mechanism that enables the formation of a new species in one step (Feldman and Levy, 2005). The rapid genomic structural and functional alterations accompanied with an allopolyploidization process have been intensively studied in recent years (Ramírez-González et al., 2018; Salina and Adonina, 2018; Fox et al., 2020; Juery et al., 2020). While currently there is still a debate regarding the extent of TE activity following allopolyploidization in wheat (amplification bursts vs. slow accumulation), there is no doubt that rapid TE turnover occurred during the wheat group evolution (Wicker et al., 2018; Bariah et al., 2020). The great contribution of TEs to genome plasticity might affect the ability of the new polyploid species to survive and rapidly adapt to various biological, environmental, and even cultivation environment stress (Van De Peer et al., 2017; Levy and Feldman, 2022).

The huge number of TE insertions adjacent to wheat genes led researchers to investigate the possible role of TEs in gene regulation (Wicker et al., 2018; Keidar-Friedman et al., 2020). Wicker et al. (2018) found no strong associations between specific TE families found near promoters and various expression modules. Additionally, a study by Ramírez-González et al., 2018 focused on the effect of TE insertions within triads (homeologs with a 1:1:1 correspondence across the three bread wheat sub-genomes- ABD) genes promoters and found no correlation between the presence of TEs in gene promoters and altered expression patterns between the three homeolog genes. However, Ramírez-González et al., 2018 did observe that higher TE abundance in the vicinity of the translation start site correlated with triads that showed more dynamic expression patterns across different tissues. This observation led Ramírez-González et al., 2018 to suggest a possible role for TEs in gene regulation as cis-regulatory elements or through other epigenetic mechanisms in a tissue-specific manner. Moreover, recent studies showed that TEs, specifically MITEs (Miniature Inverted repeat TEs), which are prevalent in the vicinity of wheat genes, might act as miRNAs precursors in wheat and thus can potentially shape regulatory gene networks (Poretti et al., 2019; Crescente et al., 2022). While the effect of TE insertions into promoter regions in wheat has been well-investigated, very little is known about the possible effect of TE insertions within gene bodies (Li et al., 2014; Xi et al., 2016; Keidar et al., 2018; Keidar-Friedman et al., 2018; Domb et al., 2019; Jiang et al., 2019). Here, the analysis of a large amount of publicly available databases in bread wheat facilitated the assessment of the functional impact of TE insertions within gene bodies in a genome-specific manner.

## 2 Methods

### 2.1 Identification of TE insertions within gene bodies

To identify TE insertions within gene bodies (exons\ introns) in the *Chinese Spring* bread wheat cultivar (*CS42*), we integrated data from two publicly available databases (Appels et al., 2018; Juery et al., 2020). The name, homoeologous group IDs, assignment to one of the five chromosomal regions (R1, R2a, C, R2b, and R3), and the start and stop positions of 70,818 wheat genes belonging to 6,320 dyads (12,640 genes belongs to homoeologous groups that underwent elimination of a single gene), 18,390 triads (55,170 genes), and 752 tetrads (3,008 genes, belongs to homoeologous groups that underwent duplication of a single gene) were retrieved from Juery et al. (2020) and integrated with the IWGSC RefSeq v1.0 assembly coordinates for TEs (Appels et al., 2018) using python 3.7 (Guttag, 2021) scripts. Prior to the data integration, the IWGSC RefSeq v1.0 assembly annotations for TEs were organized using pandas, a Python package used for data analysis (Reback et al., 2021), and filtered to include only repeats defined as “repeat region” (nested repeats and repeat fragments were removed). Next, overlaps between repeat regions and each of the 70,818 genes were detected based on the genes and TEs coordinates and summarized in **Supplementary Table S1**. To compare the proportions of TE-containing genes between dyads, triads, and tetrads, the Chi-square test of independence of variables was performed using the *chi2_contingency* function from Python *SciPy* (RRID : SCR_008058)*. Stats* module (Virtanen et al., 2020).

### 2.2 Polymorphic TE insertions within gene bodies

Following the identification of TE insertions within gene bodies and the characterization of TE insertions distribution, we wanted to assess the polymorphism(s) generated by TE insertions between the homoeologous copies in dyads, triads, and tetrads. For this, we used the *pandas* Python package (RRID : SCR_018214) (Reback et al., 2021) to organize the genes (see **Supplementary Table S1**) as homoeologous groups (**Supplementary Table S2**) according to the homoeologous group IDs and to sum the number of genes which contained one or more TE insertion within the gene body in each expressed homoeologous group (a group that includes one or more expressed gene, not expressed groups were removed from the analysis). For each of the homoeologous groups, we determined whether it was a polymorphic or monomorphic group. If all the homoeologs in a specific homoeologous group contained TE insertion (not depending on TE type or insertion location within the gene), the homoeologous group was considered as monomorphic. However, if one or more, but not all, of the homoeologs in the group contained TE insertion, the homoeologous group was considered polymorphic.

To test whether TE insertions were randomly distributed between the genes or rather tend to be more\ less polymorphic than expected, we focused only on homoeologous groups that included TE insertions in one or more of the homoeologs gene bodies (referred to as homoeologous groups that include TE insertions) and were determined to be expressed (include one or more expressed gene). Then, we performed the Chi-square Goodness of Fit Test separately for dyads, triads, and tetrads, to test whether the numbers of monomorphic and polymorphic homoeologous groups fit the expected numbers calculated based on the proportions of gene bodies that contain TE insertions. The expected number of monomorphic and polymorphic homoeologous groups was calculated assuming a binomial distribution of the presence \ absence of TE insertions within a gene body. First, the probability of a single gene including TE insertion was calculated based on the number of genes containing TE insertions and belonging to TE containing homoeologous group and the total number of genes belonging to TE containing homoeologous group. Then, the expected number of monomorphic and polymorphic homoeologous groups was calculated according to binomial distribution using the probability of a single gene including TE insertion and then divided by the probability of a homoeologous group to have at least one TE insertion (conditional probability) and multiplied by the number of groups containing one or more TE insertion. The observed numbers of polymorphic and monomorphic homoeologous groups were compared with the calculated expected numbers using the *chisquare* function from Python *SciPy* (RRID : SCR_008058)*. Stats* module (Virtanen et al., 2020).

### 2.3 Correlation between polymorphic TE insertions within gene bodies and homoeolog expression bias

To assess the possible impact of TE insertional polymorphism within gene bodies on the relative gene expression in the homoeologous groups, we used summarized data “relative contribution category in brief” retrieved from Juery et al. (2020), on the assignment of each of the homoeologous groups to relative contribution categories. Based on this analysis, if all the homoeologs in a specific homoeologous group had similar relative abundance, the group was assigned to the balanced category, while groups in which different relative abundance was observed between the homoeologs were assigned to one of the non-balanced categories (Juery et al., 2020). Specifically, triads were assigned to the balanced category, homoeolog-suppressed category, or homoeolog-dominant category, dyads were assigned to a balanced category or homoeolog-suppressed category, and tetrads were assigned to either one of the following categories: balanced category, tetrads with one suppressed copy, tetrads with two suppressed copies, and tetrads with one dominant copy (Juery et al., 2020). Additionally, some of the homoeologous groups were referred to as not expressed and thus were excluded from further analysis. The assignment of the homoeologous groups to relative contribution categories was performed according to the calculation method described by Ramírez-González et al. (2018) and based on the same RNA-seq data used by Ramírez-González et al. (2018) for 123 samples of bread wheat (*Chinese Spring*) taken from 15 different tissues under non-stress conditions (Juery et al., 2020). The dependency between polymorphism and balanced\ non-balanced expression of the homoeologs was tested using the Chi-square test of independence of variables with the *chi2_contingency* function from Python *SciPy. Stats* module (Virtanen et al., 2020).

### 2.4 Correlation between TE insertion within gene bodies and homoeolog expression bias in triads

Here we used data on the relative expression abundance of the homoeologs in each of the triads (Ramírez-González et al., 2018) and the IWGSC RefSeq v1.0 assembly annotations for genes and TEs (Appels et al., 2018) to assess the possible impact of TE insertions on gene expression variations of homoeologous groups. We used 55,422 genes that had a 1:1:1 correspondence across the three homoeologous subgenomes (A, B, and D) of bread wheat (18,474 homoeolog triads) from Ramírez-González et al. (2018) and identified TE insertion within each of the genes bodies as described in **Supplementary Table S1** (see **Supplementary Table S3**). For each of the triads, the TE classification (superfamily and subfamily) was determined for elements that were found to be inserted within the gene bodies of the genes in the triad (**Supplementary Tables S4**, **S5**). TE subfamily names were according to the ClariTeRep naming system (Wicker et al., 2018), in which the three first letters of the subfamily name represent the TE superfamily, and the number at the end of the name represents the family and in some cases is followed by a dot and a number, which represents specific subfamily within the TE family.

In addition to the identification of TE insertions within gene bodies, we identified TE insertions found specifically within exons and within the UTRs using a similar approach, combining the exons, 5’ UTRs, and 3’ UTRs coordinates for each gene according to IWGSC RefSeq v1.0 HC genes annotations with TEs coordinates (Appels et al., 2018). Then, we integrated data from files dividing the homoeolog triads into seven relative contribution categories (Ramírez-González et al., 2018), to create **Supplementary Table S3**.

The seven files divided the triads into contribution categories as follows: triads for which a similar abundance of transcripts was observed from each of the three homoeologs were assigned to a balanced category, while triads that showed a higher or lower abundance of transcripts from a single homoeolog relative to the other two, were assigned to one of six non-balanced categories. The non-balanced categories include three homoeolog-dominant categories (A dominant, B dominant, and D dominant) and three homoeolog-suppressed categories (A suppressed, B suppressed, and D suppressed) (Ramírez-González et al., 2018). Each triad was attributed to one of the above categories based on ternary diagrams representing the relative expression of each homoeolog and by comparison to the ideal normalized expression bias for the seven categories as described by Ramírez-González et al., (Ramírez-González et al., 2018). The analysis was performed for RNA-seq data from several different studies (Ramírez-González et al., 2018) (a total of 850 wheat RNA-sequencing samples), which were organized into partly overlapping datasets. Here we focused on data generated from 123 RNA-Seq samples of bread wheat (*Chinese Spring*) (Ramírez-González et al., 2018). The 123 samples were derived from 15 different tissues under non-stress conditions. For our analysis, we focused on 14,258 triads which were found to be syntenic and expressed in at least 6 out of 15 tissues tested for this dataset (see **Supplementary Table S3**) (Ramírez-González et al., 2018).

For the following analysis, the three homoeolog genes in each one of the 14,258 triads were combined, meaning that a triad was referred to as a triad that included TE insertions if one or more TE insertions were found within the sequence of at least one of the triad genes. The dependency between the presence\ absence of TE insertions within the gene bodies, exons, or UTRs and different contribution categories was analyzed using the Chi-square test of independence of variables with the *chi2_contingency* function from Python *SciPy. Stats* module (Virtanen et al., 2020). The dependency between the TE superfamilies\ subfamilies from which insertions were present\ absent in at least one of the triad genes and the different contribution categories was analyzed using the Chi-square test of independence of variables with the *chi2_contingency* function from Python *SciPy. Stats* module and corrected for multiple testing using the *multipletests* function from the Python *statsmodels* module (RRID : SCR_016074) with the Benjamini/Hochberg Procedure (non-negative) (Virtanen et al., 2020).

### 2.5 Gene ontology enrichment analysis

Gene ontology (GO) provides structured, computable knowledge regarding the functions of genes and gene products in three non‐overlapping domains of molecular biology (Carbon et al., 2019). The three domains are Biological Process (BP), which refers to a biological objective to which the gene or gene product contributes, Molecular Function (MF), defined as the biochemical activity of a gene product and Cellular Component (CC), which refers to the location in the cell where a gene product is active (Ashburner et al., 2000). GO enrichment analysis is used to find over-represented GO terms in a gene set compared to a reference set.

Here, we performed GO enrichment analysis for triads, including TE insertions from each of the 14 TE superfamilies (see **Table 1**). Additionally, we selected triads that belonged to specific relative contribution categories and included TE insertions from superfamilies that showed a correlation to the mentioned category (see **Table 2**). The reference set for all the GO enrichment analyses performed in this study was the whole set of 14,258 expressed and syntenic triads. GO Singular Enrichment Analysis (SEA) was performed using the AgriGO toolkit (RRID : SCR_006989) (Tian et al., 2017) with Fisher’s exact test to identify enriched GO terms.

**Table 1:** Analysis of the 7,439 expressed and syntenic triads which contained TE insertions belonging to 14 different TE superfamilies.

					GO classes^3^		
Code^1^	Class	Order	Superfamily	Triads^2^	BP	CC	MF
RLG	Class I (retrotransposons)	LTR	*Gypsy*	1,134	438	70	148
RLC			*Copia*	1,359	371	67	157
RLX			Unclassified LTR-retrotransposons	474	132	15	84
RIX		non-LTR (LINE)	Long interspersed nuclear elements	1,458	416	97	209
SIX		non-LTR (SINE)	Short interspersed nuclear elements	20	19	11	23
DTC	Class II (DNA transposons)	TIR	*CACTA*	2,960	734	140	327
DTM			*Mutator*	428	185	11	80
DTX			unknown	2,099	606	89	248
DTH			*Harbinger*	456	142	21	94
DTT			*Mariner*	4,576	956	156	400
DTA			*hAT*	9	7	1	4
DXX		unknown	unknown	105	68	15	22
DHH		Helitron	*Helitron*	2	–	–	–
XXX	unknown	unknown	unknown	1,347	266	16	225

^1^ The three letters code represents the class (first letter), order (second letter) and superfamily (third letter) of the TE (Wicker et al., 2007).

^2^ Number of triads in which at least one of the genes includes TE insertion from the specific superfamily. Note that the sum of the triad column is larger than 7,439. This is since some triads include insertions from more than one subfamily.

^3^ Number of significantly enriched GO terms found in GO SEA preformed for triads which include TE insertions from mentioned superfamily in each of the three biological objective to which the gene or gene product contributes: BP, Biological Process, CC, Cellular Component and MF, Molecular Function.”-” notes missing values due to short query list which did not met the criteria for enrichment analysis.

**Table 2:** TE superfamilies for which the presence\absence of TE insertion in at least one of the triad genes correlated with specific triad expression patterns.

		TE insertion			
TE superfamily^1^	corrected p-values^2^	Yes	No	Yes	No
		Balanced triads^3^		Non-balanced triads^4^	
**RLC**	0.008837323	1132	5252	227	828
**DTT**	6.26E-06	4001	2383	575	480
**DTM**	0.000236883	338	6046	90	965
**DTX**	0.01607886	1837	4547	262	793
**SIX**	0.008282664	12	6372	8	1047
**RLX**	0.00382206	382	6002	92	963
**XXX**	0.01015199	1123	5261	224	831
		**Suppressed triads^5^**		**Not suppressed triads^6^**	
**RLC**	0.014455	192	690	1167	5390
**DTT**	0.000675	488	394	4088	2469
**DTM**	0.023678	68	814	360	6197
**DTX**	0.047574	220	662	1879	4678
**RLX**	0.000675	83	799	391	6166
**XXX**	0.006588	194	688	1153	5404
		**Dominant triads^7^**		**Not dominant triads^8^**	
**DTT**	0.013897	87	86	4489	2777
**DTM**	0.001364	22	151	406	6860

^1^ The three letters code represents the class (first letter), order (secuned letter) and superfamily (third letter) of the TE (Wicker et al., 2007).

^2^ χ^2^ corrected p-values for multiple tests using Benjamini/Hochberg Procedure (non-negative).

^3,5,7^ Number of triads belonging to the mentioned category (balanced, suppressed, or dominant), in which at least one homoeolog contains TE insertion from the mention superfamily (Yes) or none of the homoeologs contain TE insertion from the mention superfamily (No).

^4,6,8^ Number of triads that does not belong to the mentioned category, in which at least one homoeolog contains TE insertion from the mention superfamily (Yes) or none of the homoeologs contain TE insertion from the mention superfamily (No). For the balanced category it will refer to the number of triads from the homoeolog-dominant or homoeolog-suppressed categories, for the suppressed categories it will refer to the number of triads from balanced or to one of the homoeolog-dominant categories and for the dominant categories it will include triads belong to either the balanced or to one of the homoeolog- suppressed categories.

Following the GO SEA, enriched GO terms for each GO category (Biological function, Cellular component, and Molecular function) were visualized as a scatter plot generated by REVIGO (RRID : SCR_005825) (Supek et al., 2011). REVIGO summarizes the GO terms lists generated from the GO SEA by reducing functional redundancies based on the value provided and visualizes the remaining GO terms as a scatterplot, where more semantically similar GO terms are found closer to each other in the plot. For each GO SEA, we provided REVIGO, a list of GO terms that were found to be significantly enriched with false discovery rate (FDR) less or equal to 0.05 and their FDR value which is an adjusted p-value that enables us to have less false positive results then if the p-value was used. The scatterplots generated by REVIGO were imported into R, where wanted labels were added, and others were moved manually to slightly different coordinates to better visualize all the labels.

## 3 Results

### 3.1 Different TE insertion patterns within gene bodies in dyads, triads, and tetrads

**Figure 1:**
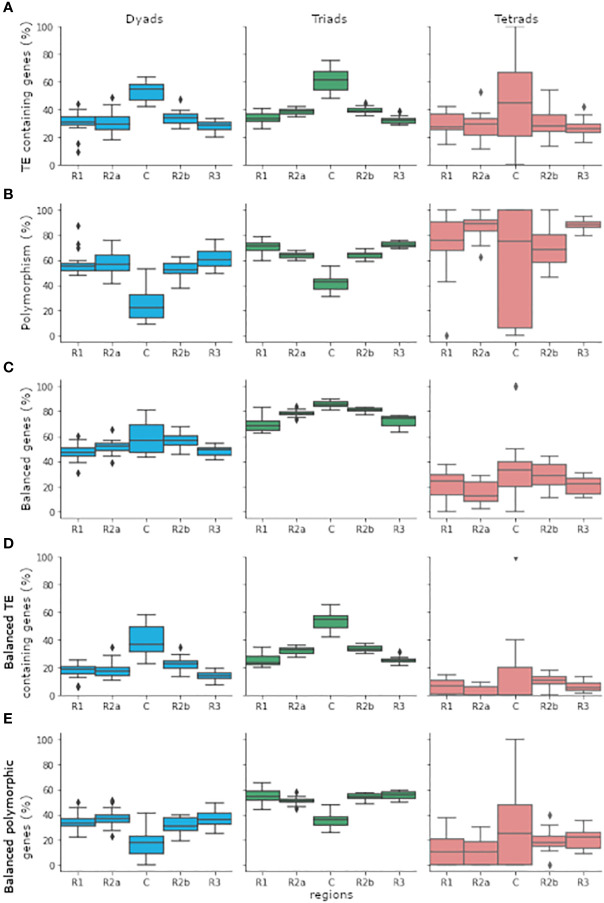
The distribution of genes belonging to the dyads, triads, or tetrads categories in the five chromosomal regions (R1, R2a, C, R2b, and R3). Gene distribution was calculated individually for genes that belong to the same category and found in the same and chromosomal region in each one of 18 bread wheat chromosomes. Genes found in chromosomes 1A, 1B, and 1D were eliminated from the analysis due to the lack of tetrads genes in the centromeres of chromosomes 1A and 1B. **(A)** Percentage of TE containing genes out of total genes. **(B)** Percentage of genes belonging to polymorphic group out of genes that found in TE containing group. **(C)** Percentage of genes belonging to balanced group out of total genes. **(D)** Percentage of genes belonging to balanced group out of genes that found in TE containing group. **(E)** Percentage of genes belonging to balanced and polymorphic group out of genes that found in TE containing group. The boxplots depict the first quartile (Q1) and the third quartile (Q3) of the data with the median between them. The whiskers extend from the box to 1.5x the interquartile range (IQR). Rhombuses represent values found past the end of the whiskers. boxplots were drawn using the *boxplot* function from the seaborn python package (Waskom, 2021).

In order to perform a genome-wide analysis of TE insertions within wheat gene bodies, 70,818 bread wheat genes belonging to 6,320 dyads, 18,390 triads, and 752 tetrads were analyzed. TE insertions within gene bodies were identified based on the IWGSC RefSeq v1.0 assembly coordinates for HC genes and TEs (Appels et al., 2018). We found that ~36% of the 70,818 genes (25,811 genes) contain at least one TE insertion within the gene body, with higher proportions of TE containing genes observed for triads (20,975 genes, 38.02%) relative to dyads (3,972 genes, 31.42%) and tetrads (864 genes, 28.72%) (**Figure 1A**; **Supplementary Figure S1**). The difference in the proportions of TE containing genes between the dyads, triads, and tetrads genes was statistically significant (χ^2^ = 273.99, p < 0.001). TE insertions were found either in all the homoeologous copies in the group (i.e., for triads: monomorphic insertion in the three sub-genomes) or only in some of the homoeolog genes (i.e., for triads: polymorphic insertion in the three sub-genomes).

The differences in TE abundance between the dyads, triads, and tetrads categories might be the result of the different chromosomal distribution patterns of the genes between the categories. While triads are more abundant in the proximal region (R2a, C and R2b), which contains a higher proportion of TEs, dyads, and tetrads are most abundant in the distal region (R1 and R3), which was found to have lower TE density (Wicker et al., 2018; Juery et al., 2020). The proportions of TEs containing genes belonging to each of the three categories in each of the five chromosomal regions are shown in **Figure 1A**. To test whether the difference in TEs abundant between the dyads, triads, and tetrads is mainly due to the chromosomal distribution of the genes, we performed the analysis separately for the proximal and distal regions. Significant differences in TEs abundant within gene bodies from dyads, triads, and tetrads were observed for each region separately, displaying the same pattern observed for the whole genome. Out of the 41,503 genes (4,781 belonging to dyads, 35,447 to triads, and 1,275 to tetrads) found in the proximal region, 40.29% included TE insertion within the gene body, with significant differences in proportion (χ^2^ = 135.78, p < 0.001) between dyads (1,665 genes, 34.83%), triads (14,675 genes, 41.40%), and tetrads (380 genes, 29.80%) genes. A lower proportion of TE containing genes was observed in the distal regions, where only 31.01% of the 29,315 genes (7,859 belonging to dyads, 19,723 to triads, and 1,733 to tetrads) included TE insertion. However, the significant differences in proportion (χ^2^ = 25.77, p < 0.001) of TE containing genes were still observed in the distal region, with a higher proportion of TE insertion in triad genes (6,300 genes, 31.94%) relative to dyads (2,307 genes, 29.35%) and tetrads (484 genes, 27.93%).

### 3.2 Polymorphic TE insertions within gene bodies and homoeologous group expression patterns

To assess the associations between TE insertion patterns and gene expression in dyads, triads, and tetrads, we first grouped the homoeologs copies from each homoeologous group and determined for each expressed group (a group that includes one or more expressed gene) whether it is monomorphic or polymorphic. Homoeologous group was considered as monomorphic if all the homoeologs copies in the group included at least one TE insertion or polymorphic if at least one but not all the homoeologs copies in the group included TE insertion within the gene body (**Figure 1B**, **Supplementary Table S2**). Then, the number of monomorphic and polymorphic homoeologous groups was compared with the numbers predicted by our module, which was based on the assumption that the presence\ absence of TE insertions within a gene is random. Out of 5,059 expressed dyads, 47.16% (2,386 dyads) included TE insertions, meaning at least one TE insertion was present within one or more of the homoeologs gene bodies. Focusing only on the 2,386 dyads that include TE insertions, we found that 54.99% (1,312 dyads) were polymorphic and included TE insertions in only one of the homoeologs gene bodies, a result that did not fit our module (χ^2^ = 136.78, p < 0.001) which predicted that only 43.13% (~1,029 dyads) would be polymorphic. While the percentage of polymorphic dyads was higher than our module anticipated, the opposite trend was observed for triads and tetrads. Out of 17,676 expressed triads, 59.24% (10,471 triads) included TE insertions, with 64.08% of the triads identified as polymorphic (6,710 triads), a distribution that did not match our module (χ^2^ = 212.36, p < 0.001), which predict that 70.57% of the triads (~7390 triads) will be polymorphic. For tetrads, we found that out of the 667 expressed tetrads, 58.17% (388 tetrads) included TE insertions, and 82.73% (321 tetrads) of the TE insertions containing tetrads were found to be polymorphic. This result also did not fit our module (χ^2^ = 43.47, p < 0.001), which predicts that 91.88% of the tetrads (~356 tetrads) will be polymorphic.

To reduce the effect of the different chromosomal distribution patterns of dyads, triads, and tetrads on our analysis, we reperformed the analysis focusing on homoeologous groups in which all the homoeologs copies were found in the same chromosomal region (proximal\ distal). Thus, the numbers of monomorphic and polymorphic homoeologous groups and the predicted distribution were counted and calculated separately for dyads, triads, and tetrads in each chromosomal region. Similar patterns to those observed for the whole chromosome were observed separately for the proximal and distal regions. Out of the 2,386 expressed dyads that include TE insertions, 872 included only genes found in the proximal region, and 1,288 included only genes found in the distal region. The numbers of polymorphic dyads in both the proximal and distal regions were higher than expected by our module. They did not match our predictions (proximal region: χ^2^ = 33.11, p < 0.001, distal region: χ^2^ = 95.11, p < 0.001), with 48.62% polymorphic dyads (424 dyads) at the proximal region and 59.24% polymorphic dyads (763) at the distal region. In contrast, our module predicted that 39.19% of the dyads found in the proximal region (~341 dyads) and 45.70% of the dyads in the distal region would be polymorphic. For triads, we found that out of the 10,471 expressed TE containing groups, 6,654 triads included only genes located in the proximal region and 2,901 triads included only genes located in the distal region. The number of polymorphic triads was lower than the number predicted by our module (proximal region: χ^2^ = 154.72, p < 0.001, distal region: χ^2^ = 28.96, p < 0.001), with 59.78% polymorphic triads (3,978 triads) at the proximal region and 72.84% polymorphic triads (2,113 triads) in the distal region, versus 66.96% in the proximal region (~4455 triads) and 77.04% in the distal region (~2,235 triads) predicted by our module. The distribution of polymorphic tetrads also did not fit the numbers predicted by our module (proximal region: χ^2^ = 25.53, p < 0.001, distal region: χ2 = 16.65, p < 0.001). Out of the 388 expressed tetrads which include TE insertions, 132 tetrads included only genes located in the proximal region, and 79.55% (105 tetrads) of them were found to be polymorphic, while our module predicted that 91.69% of the tetrads (~121 tetrads) in the proximal region would be polymorphic. Moreover, out of the 181 TE containing expressed tetrads that included only genes located in the distal region, only 83.43% were found to be polymorphic, while our module predicted that 91.76% of the tetrads in the distal region (~166 tetrads) would be polymorphic.

Next, we aimed to assess whether polymorphic TE insertions affect the relative expression within the homoeologous group. The data on the relative expression within each homoeologous group was retrieved from Juery et al. (2020), which performed the analysis for 123 RNA-Seq samples of bread wheat (*Chinese Spring*) taken from 15 different tissues under non-stress conditions, and was integrated into **Supplementary Table S2**. Expressed homoeologous groups were assigned as balanced if all the homoeologs showed similar transcript abundance or as homoeolog-dominant or homoeolog-suppressed (non-balanced), based on the relative higher\ lower transcript abundance of each homoeolog (**Figure 1C**) (Juery et al., 2020). To learn about the possible effect of TE insertions within gene bodies on the relative expression, we focused only on TE containing homoeologous groups and tested the correlation between polymorphism and expression patterns separately for dyads, triads, and tetrads (**Figures 1B, D, E**). Our analysis revealed that a higher percentage of the polymorphic homoeologous groups belonged to one of the non-balanced expression categories relative to monomorphic groups, with a significant difference in proportions for dyads and triads (dyads: χ^2^ = 11.34, p < 0.001, triads: χ^2^ = 73.45, p < 0.001), while for tetrads the differences in proportions were not statistically significant (χ^2^ = 0.32, p = 0.57). However, different results were obtained when the analysis was performed separately for the proximal and the distal chromosomal regions. For dyads, 37.88% (497 dyads) of the 1,312 polymorphic groups showed non-balanced expression, while only 31.19% (335 dyads) of the 1,074 monomorphic groups showed non-balanced expression. A statistically significant correlation (χ^2^ = 9.96, p = 0.002 < 0.05) was also identified between polymorphism and relative expression pattern for dyads that contained only genes found in the proximal region (872 dyads), with 32.55% of the polymorphic dyads (138 dyads) and 22.77% of the monomorphic dyads (102 dyads) found in one of the non-balanced categories. However, no significant correlation was found between polymorphism and relative expression patterns for dyads which include only genes located in the distal region (χ^2^ = 0.05, p = 0.82 > 0.05), although higher proportions of polymorphic dyads were found in non-balanced categories (37.75%, 288 dyads) relative to monomorphic dyads (36.95%, 194 dyads). Similar to dyads, for triads, 18.21% (1,222 triads) of the 6,710 polymorphic groups were classified as non-balanced, compared to 11.81% (444 triads) of the 3,761 monomorphic triads that were classified as non-balanced. The correlation between non-balanced expression and polymorphism in triads was also observed separately for triads that include only genes located in the proximal region (χ^2^ = 52.98, p < 0.001) and for triads that include only genes located at the distal region (χ^2^ = 5.54, p = 0.02 < 0.05). At the proximal region, 16.37% of the polymorphic triads (651 triads) and 10.05% of the monomorphic triads (269 triads) were classified as non-balanced, and at the distal region, 21.15% of the polymorphic triads (447 triads) and 17.13% of the monomorphic triads (135 triads) were classified as non-balanced. Finally, for tetrads, 77.26% (248 tetrads) of the 321 polymorphic groups and 73.13% (49 tetrads) of the 67 monomorphic groups were assigned to one of the non-balanced categories. No statistically significant dependency between polymorphism and homoeologous group expression pattern was identified upon performing the analysis separately for tetrads which include genes located only at the proximal (χ^2^ = 0.43, p = 0.51 > 0.05) or only at the distal (χ^2^ = 0.02, p = 0.89 > 0.05) chromosome regions.

### 3.3 TE content within gene bodies and triad expression patterns

Here, we aimed to study the possible effect of TE insertions on gene expression in wheat. We analyzed 14,258 expressed and syntenic triads that were assigned to 7 relative contribution categories according to the calculation method described by Ramírez-González et al. (2018). Most of the 14,258 triads (11,834 triads, 83%) showed balanced expression, meaning a similar relative abundance of transcripts was observed for the three homoeologs. The remaining 2,424 triads were divided between 6 non-balanced categories, with 13.99% of the triads (1,995 triads) assigned to one of the homoeolog-suppressed relative contribution categories (5.16% of the triads belonged to the A suppressed category, 5.31% to the B suppressed category and 3.52% to the D suppressed category) and 3.01% of the triads (429 triads) assigned to one of the homoeolog-dominant relative contribution categories (0.90% of the triads belonged to the A dominant category, 1.05% to the B dominant category and 1.07% to the D dominant category). TE insertions within the gene bodies of triads genes were identified based on the IWGSC RefSeq v1.0 assembly coordinates for high confidence (HC) genes and TEs (Appels et al., 2018).

The analysis of the 14,258 expressed and syntenic triads revealed that the presence of TE insertions in at least one of the triad genes (7,439 triads) correlated to balanced expression between the homoeolog genes. Out of the 14,258 expressed and syntenic homoeolog triads, 52.17% (7,439 triads) contain one or more TE insertions (based on repeat regions coordinates) within the gene body sequence of at least one of the genes in the triad (triads that include TE insertions). A higher proportion of triads that include TE insertions are found in the balanced expression category (6,384, 85.82%) relative to triads that don’t include TE insertions (5,450, 79.92%) with a statistically significant difference in proportions (χ^2^ = 87.18, p < 0.001).

The TEs that were found to be within gene bodies represented all the 14 TE superfamilies identified in the wheat genome (see **Table 1**) and belonged to 455 subfamilies out of the 570 subfamilies annotated by the IWGSC as “repeat region”, as was counted from the annotation file (Appels et al., 2018). To learn about the possible association between TE type and the relative expression contribution of each of the homoeologs in the triad, we tested separately for each TE superfamily and subfamily whether the presence\ absence of TE insertions from said type within gene bodies correlated with balanced, suppressed, or dominant relative expression of the homoeologs. Here, we focused only on TE groups (superfamily or subfamily) that had sufficient sample size, mining 5 or more cases were observed for all the combinations of the tested conditions for the group with the examined relative expression category. For instance, the number of triads in which TE insertions from specific TE superfamily were presence\ absent must be five or higher both in balanced and non-balanced categories for the superfamily to be included in the analysis against the balanced relative expression category. Out of the 14 TEs superfamilies, 12 were found adequate for analysis against the balanced expression category (DTA and DHH were removed from the analysis), and 7 TEs superfamilies showed a statistically significant correlation (Chi-square corrected p-value ≤ 0.05, **Table 2**) with balanced\ non-balanced expression categories. The correlation between superfamily and balanced expression was negative for 5 (SIX, DTM, RLX, RLC, and XXX, **Table 2**) of the 7 superfamilies and positive for the remaining 2 superfamilies (DTT and DTX). The same 12 superfamilies that were found adequate for analysis against the balanced expression category were also found adequate for comparison against homoeolog-suppressed\ non-suppressed expression categories, with the remaining 2 superfamilies (DTA and DHH, **Table 2**) excluded from the analysis due to a low number of cases. Specific superfamilies also showed a statistically significant correlation with homoeolog-suppressed\ non-suppressed expression categories. In total, 6 superfamilies showed statistically significant correlation with homoeolog-suppressed\ non-suppressed expression categories (Chi-square corrected p-value ≤ 0.05, **Table 2**), all of them also showed correlation with balanced\ non-balanced expression categories. TEs superfamilies that showed a positive correlation with balanced expression showed a negative correlation with suppressed expression (DTT and DTX, **Table 2**), while TEs superfamilies that showed a negative correlation with balanced expression showed a positive correlation with suppressed expression (DTM, RLX, RLC and XXX, **Table 2**). Finally, only 10 of the 14 superfamilies were found fitted for analysis against the homoeolog-dominant\ non-dominant expression categories (SIX, DTA, DXX, and DHH were removed from the analysis), with only 2 TE superfamilies (DTM and DTT) showing statistically significant correlation with homoeolog-dominant\ non-dominant expression categories (Chi-square corrected p-value ≤ 0.05, **Table 2**), both also found to correlate with balanced expression significantly. The DTM superfamily showed a positive correlation with dominant expression and a negative correlation with balanced expression, while the DTT superfamily showed a negative correlation with dominant expression and a positive correlation with balanced expression.

Next, we performed a similar analysis for TE subfamilies. The majority of the 455 TE subfamilies found within gene bodies were excluded from the analysis due to the small sample size: out of the 455 TE subfamilies, 303 subfamilies were eliminated from the analysis for the balanced expression category, 323 subfamilies were excluded from the analysis for the suppressed expression categories, and 433 subfamilies were excluded from the analysis for the dominant expression categories. Out of the TEs subfamilies which were found adequate for analysis, 19 subfamilies showed a statistically significant correlation (Chi-square corrected p-value ≤ 0.05, **Table 3**) with balanced\ non-balanced expression categories, 17 subfamilies showed a statistically significant correlation (Chi-square corrected p-value ≤ 0.05, **Table 4**) with homoeolog-suppressed\ non-suppressed expression categories and none of the TE subfamilies showed statistically significant correlation with homoeolog-dominant\ non-dominant expression categories. Fourteen of the subfamilies that showed a significant correlation between presence\absence of TE insertions and suppression of a single homoeolog gene also showed a correlation with balanced relative expression of the homoeologs, while the other subfamilies were found in correlation only to suppressed (3 subfamilies) or balanced (5 subfamilies) relative expression. Of the 17 subfamilies that showed statistically significant correlation with homoeolog-suppressed expression categories, only the *DTT_famn14* subfamily showed a negative correlation with homoeolog-suppressed expression, while insertions of the remaining 16 subfamilies appeared in higher proportions than expected in the homoeolog-suppressed categories. Similarly, 17 of the 19 subfamilies that showed a statistically significant correlation with homoeolog-balanced expression showed a negative correlation with balanced expression, and only two subfamilies, *RIX_famc8* and *DTT_famn14*, showed a positive correlation with homoeolog-balanced expression.

**Table 3:** TE subfamilies for which the presence/absence of TE insertion in at least one of the triad genes correlated with balanced relative expression of the three homoeologs.

CLARITE name^1^	corrected p-values^2^	TE insertion			
		Yes	No	Yes	No
		Balanced triads^3^		Non-balanced triads^4^	
**DTC_famc11.1**	0.046018	23	6361	11	1044
**RLC_famc6**	0.000736	18	6366	13	1042
**RLC_famc1.6**	0.00292	6	6378	7	1048
**RLC_famc20**	9.35E-11	22	6362	24	1031
**RLC_famc7.1**	0.034407	7	6377	6	1049
**DTC_famc4.3**	0.00766	5	6379	6	1049
**DTM_famc9**	0.04758	20	6364	10	1045
**RLG_famc1.1**	0.002862	13	6371	10	1045
**RIX_famc1**	0.004706	212	6172	59	996
**RLC_famc8**	0.001236	14	6370	11	1044
**RIX_famc8**	0.029024	693	5691	82	973
**DTM_famc8**	0.04758	20	6364	10	1045
**DTT_famn14**	0.000511	721	5663	72	983
**XXX_famc13**	0.000302	99	6285	38	1017
**SIX_famc1**	0.026102	9	6375	7	1048
**XXX_famc16**	3.10E-05	119	6265	46	1009
**RLX_famc22**	0.001572	29	6355	16	1039
**XXX_famc112**	0.001236	14	6370	11	1044
**RIX_famc15**	3.23E-10	24	6360	24	1031

^1^ According to Wicker et al. (Wicker et al., 2018). TE names were selected based on the ClariTeRep naming system, which assigns simple numbers to individual families and subfamilies.

^2^ χ^2^ corrected p-values for multiple tests using Benjamini/Hochberg Procedure (non-negative).

^3^ Number of triads belonging to the homoeolog-balanced category, in which at least one homoeolog contains TE insertion from the mention subfamily (Yes) or none of the homoeologs contain TE insertion from the mention subfamily (No).

^4^ Number of triads belonging to one of the non-balanced categories, meaning to one of the homoeolog-dominant or homoeolog-suppressed categories, in which at least one homoeolog contains TE insertion from the mention subfamily (Yes) or none of the homoeologs contain TE insertion from the mention subfamily (No).

**Table 4:** TE subfamilies for which the presence/absence of TE insertion in at least one of the triad genes correlated with the suppression of a single homoeolog gene.

CLARITE name^1^	corrected p-values^2^	TE insertion			
		Yes	No	Yes	No
		Suppressed triads^3^		Not suppressed triads^4^	
**DTC_famc11.1**	0.028255	10	872	24	6533
**RLC_famc6**	0.013022	10	872	21	6536
**RLC_famc1.6**	0.000371	7	875	6	6551
**RLC_famc20**	3.85E-10	21	861	25	6532
**XXX_famc33**	0.013022	5	877	5	6552
**RLC_famc7.1**	0.008919	6	876	7	6550
**RLG_famc1.1**	0.018009	8	874	15	6542
**RIX_famc1**	0.017541	49	833	222	6335
**RLC_famc8**	0.008831	9	873	16	6541
**DTT_famn14**	0.000371	57	825	736	5821
**RLG_famc15**	0.026756	8	874	16	6541
**XXX_famc13**	3.68E-05	35	847	102	6455
**XXX_famc16**	1.02E-06	43	839	122	6435
**XXX_famc140**	0.018009	8	874	15	6542
**RLX_famc22**	0.000371	15	867	30	6527
**XXX_famc112**	7.95E-05	11	871	14	6543
**RIX_famc15**	1.88E-10	22	860	26	6531

^1^ According to Wicker et al. (Wicker et al., 2018). TE names were selected based on the ClariTeRep naming system, which assigns simple numbers to individual families and subfamilies.

^2^ χ^2^ corrected p-values for multiple tests using Benjamini/Hochberg Procedure (non-negative).

^3^ Number of triads belonging to one of the homoeolog-suppressed categories, in which at least one homoeolog contains TE insertion from the mention subfamily (Yes) or none of the homoeologs contain TE insertion from the mention subfamily (No).

^4^ Number of triads belonging to the balanced category or to one of the homoeolog-dominant categories, in which at least one homoeolog contains TE insertion from the mention subfamily (Yes) or none of the homoeologs contain TE insertion from the mention subfamily (No).

### 3.4 Triads which include TE insertions belonging to specific TE superfamilies were associated with various GO terms

**Figure 2:**
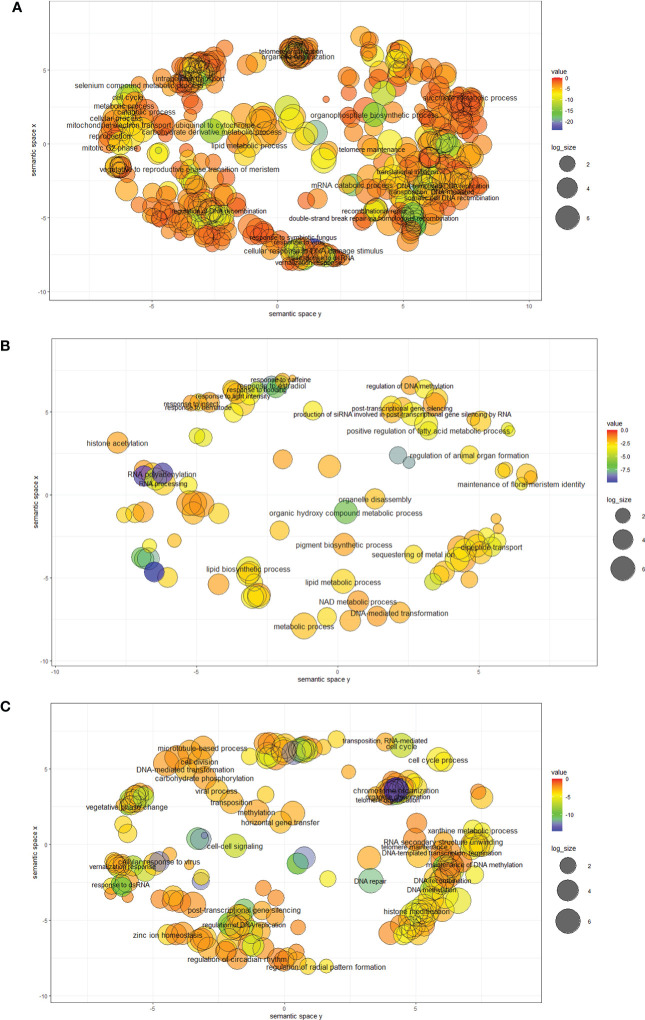
Significantly enriched GO BP (Biological prosses) terms found in GO SEA preformed for triads which include TE insertions from the DTT **(A)**, DTM **(B)** and RLC **(C)** superfamilies (TE codes are based on Wicker et al., 2007). GO SEAs were preformed using AgriGO toolkit with Fisher’s exact test (FDR ≤ 0.05). Following the GO SEA, the enriched GO terms for BP for each superfamily were visualized as a scatter plot generated by REVIGO. Closer GO terms in the plot are showing higher semantically similarity to each other. The bubble color indicates the FDR value and the size indicates the frequency of the GO term in the GOA database, bubbles of more general terms are larger.

To assess the association between the presence of TE insertions from specific types within gene bodies and gene function, we tested whether triads that include TE insertions from each of the 14 TEs superfamilies were associated with specific cellular functions. GO SEA conducted by AgriGO toolkit (Tian et al., 2017) against the database of the 14,258 expressed and syntenic triads revealed that triads which include TEs from each of 13 specific superfamilies were enriched for numerous GO terms from the BP, MF, and CC domains (**Table 5**; **Figure 2** and **Supplementary Figures S2-S13**, **Supplementary Tables S6-S25**). The DTA superfamily was excluded from the analysis due to the small sample size.

**Table 5:** Significantly enriched GO terms found in GO SEA preformed for triads which include TE insertions from mentioned superfamily in each of the three GO domains classes: Biological Process (BP), Cellular Component (CC), and Molecular Function (MF).

Class	GO term	GO ID	RLG^1*^	RLC^1*^	RLX^1*^	RIX^1*^	SIX^1*^	DTC^1*^	DTM^1*^	DTX^1*^	DTH^1*^	DTT^1*^	DTA^1*^	DXX^1*^	XXX^1*^
BP	gene silencing by RNA	GO:0031047	+	+	–	+	–	+	–	+	+	+	–	–	+
	Cell cycle	GO:0007049	+	+	–	+	–	+	–	+	–	+	–	–	+
	Organelle organization	GO:0006996	+	+	+	+	–	+	–	+	–	+	–	–	–
	Recombinational repair	GO:0000725	+	+	–	+	–	+	–	+	–	+	–	–	+
	DNA recombination	GO:0006310	+	+	–	+	–	+	–	+	–	+	–	+	+
	Telomere organization	GO:0032200	+	+	–	+	–	+	–	+	–	+	–	–	–
	DNA-templated DNA replication	GO:0006261	–	–	–	–	–	+	–	+	–	+	–	–	–
	Transposition, RNA-mediated	GO:0032197	–	+	–	–	–	+	–	–	–		–	–	–
	DNA methylation	GO:0006306	+	+	–	+	–	+	–	+	+	+	–	–	+
	Transposition, DNA-mediated	GO:0006313	–	–	–	–	–	–	–	+	–	+	–	–	–
	response to virus	GO:0009615	–	+	–	+	–	+	–	+	–	+	–	–	+
	Response to nematode	GO:0009624	+		–	–	+	–	+	–	–	+	–	–	+
	vernalization response	GO:0010048	+	+	+	–	–	+	–	–	–	+	–	–	–
	Response to symbiotic fungus	GO:0009610	–	–	–	–	–	–	–	+	–	+	–	+	–
MF	transposase activity	GO:0004803	–	–	–	–	–	–	–	+	–	+	–	–	–
	ligase activity	GO:0016874	+	+	–	+	–	+	–	+	–	+	–	–	–
	helicase activity	GO:0004386	+	+	+	+	–	+	–	+	–	+	–	–	+
	DNA-directed DNA polymerase activity	GO:0003887	–	–	–	+	–	–	–	+	–	+	–	–	–
	DNA insertion or deletion binding	GO:0032135	–	–	–	–	–	+	–	+	–	+	–	–	–
CC	DNA repair complex	GO:1990391	+	+	–	+	–	+	–	+	–	+	–	–	–
	RISC complex	GO:0016442	+	+	–	–	–	+	–	+	–	+	–	–	–
	RNAi effector complex	GO:0031332	+	+	–	–	–	+	–	+	–	+	–	–	–
	chromosome	GO:0005694	+	+	–	+	–	+	–	+	–		–	–	–
	RNA polymerase I complex	GO:0005736	–	–	+	–	–	–	–	+	–	+	–	–	–

^1^A code for each of the 13 TE superfamilies, representing the class (first letter), order (second letter) and superfamily (third letter) (Wicker et al., 2007). “+” notes that the respective GO term was found to be enriched in triads that include TE insertion from the mention superfamily. “-” notes that the respective go term was not found to be enriched in triads which include TE from the specific superfamily. GO SEA was preformed using AgriGO toolkit (Tian et al., 2017) with Fisher’s exact test (FDR ≤ 0.05).

In the BP domain, a significantly correlation was found between the presence of TEs from specific superfamilies within the triad and basic cell processes like gene silencing by RNA, cell cycle, organelle organization, recombinational repair, DNA recombination, telomere organization, DNA-templated DNA replication, and DNA methylation. Additionality, a significant correlation was found between the presence of TE insertions from specific superfamilies and response to biotic and abiotic stress, such as response to virus, response to nematode, vernalization response, and response to symbiotic fungus. Interestingly, triads that include TEs from specific superfamilies were also found to be associated with GO terms from the BP domine associated with the transposition mechanisms of the two TE classes, including transposition, RNA-mediated, and DNA-mediated (**Table 5**; **Figure 2** and **Supplementary Figures S2-S13**, **Supplementary Tables S6-S25**). For the MF domain association was observed between the presence of TE insertions from specific superfamilies within the triad and enzymes activities and that carry out basic cell processes, including ligase activity, helicase activity, and DNA-directed DNA polymerase activity and with DNA repair, including DNA insertion or deletion binding (tale 5). Similarly, for the CC domain, an association was observed with the RNA polymerase I complex, responsible for basic cell activity, and with the DNA repair complex (**Table 5**; **Supplementary Figures S2-S13**, **Supplementary Tables S6-S25**). In addition, our analysis revealed enrichment in terms associated with the regulation of gene expression, such as the RISC complex (CC) and RNAi effector complex (CC), and transposase activity (MF) (**Table 5**; **Supplementary Figures S2-S13**, **Supplementary Tables S6-S25**).

While some of the TE superfamilies were found to be significantly enriched for most of the mentioned GO terms, others showed enrichment for only a few of the GO terms we decided to focus on or even only for one of the mentioned terms (**Table 5**). For instance, triads that included TE insertions belonging to the DTT superfamily (**Figure 2A**) were found to be enriched for all the GO terms mentioned in **Table 5** except for transposition, RNA-mediated (BP), and chromosome (CC), while triads that included TE insertions belonging to the DTH superfamily showed association with only 2 of the GO terms from **Table 5**, gene silencing by RNA (BP) and DNA methylation (BP).

Following the GO SEA performed for triads that included TE insertions from specific superfamilies within the gene bodies, we further examined whether triads that showed a specific relative expression pattern and included TE insertions from specific superfamilies would associate with different GO terms relative to all the triads which include TE insertions from the same superfamily. For this purpose, we focused on triads that include TE insertions from superfamilies that we found that their presence within a triad correlated with specific relative expression patterns and are found in the relevant expression category (shown in **Table 2**). For example, triads that include DTT insertions were significantly more likely to be found in the balanced relative expression category compering to triads that included TE insertions but did not include insertions of DTT TEs, and thus, the analysis was performed for triads that included insertions belonging to the DTT superfamily and showed balanced expression of the homoeologs (**Figure 2A**; **Supplementary Figures S2-S4**, **S11-S13**, **Supplementary Tables S12 and S20**). However, for triads that include insertions belonging to the DTM superfamily, the analysis was performed separately for triads that belonged to the suppressed and the dominant relative expression categories since triads that include DTM insertions were significantly more likely to be found in suppressed or dominant relative expression category in comparison to triads that included TE insertions but did not include insertions of DTM TEs (**Figure 2B**; **Supplementary Figures S2-S10**, **Supplementary Tables S6**, **S21**, **S22**).

Generally, similar GO terms were found to be significantly enriched for the same TE superfamily when all the TE containing triads were tested and upon focusing on triads from a specific relative expression contribution category (**Supplementary Figures S2-S13**; **Supplementary Tables S6-S25**). However, we noticed that in some cases, specific terms were found to be enriched by the analysis performed for all the triads with TE insertions from specific TE superfamily and were missing from the results when the analysis was performed only for triads from specific relative expression category, or the other way around. For example, for the DTM superfamily, while the GO terms production of siRNA involved in RNA interference (GO:0030422), regulation of DNA methylation (GO:0044030), and posttranscriptional gene silencing by RNA (GO:0035194) were found to be significantly enriched when the analysis was performed for all the DTM insertions containing triads they were missing from the results of the analysis for only triads from the dominant relative expression categories, and from the results of the analysis for only triads from the suppressed relative expression categories (**Figure 2B**; **Supplementary Figures S2-S10**, **Supplementary Tables S6**, **S21**, **S22**). However, significant association with GO terms that were not found to be enriched for all the DTM continuing triads was identified for the triads that include DTM insertions and belonging to one of the homoeolog-dominant expression categories, mainly associated with response to biotic and abiotic factors and aging (aging (GO:0007568), leaf senescence (GO:0010150), organ senescence (GO: 0010260), response to metal ion (GO:0010038), response to oxidative stress (GO:0006979), response to biotic stimulus(GO:0009607), innate immune response (GO:0045087)) (**Figure 2B**; **Supplementary Figures S2-S10**, **Supplementary Tables S6**, **S21**). Similarly, significant association with GO terms that were not found to be enriched for all the DTM continuing triads was identified for the triads that include DTM insertions and belonging to one of the homoeolog-suppressed expression categories, including GO terms related to pollen formation and circadian rhythm regulation (negative regulation of circadian rhythm (GO:0042754), pollen exine formation (GO:0010584), pollen wall assembly (GO:0010208), pollen development (GO:0009555)) (**Figure 2B**, **Supplementary Figures S2-S10**; **Supplementary Tables S6**, **S22**). Another example is the differences observed in the GO SEA results performed for all triads that include TE insertions from unknown class (XXX- unclassified repeats) versus the results obtained only for triads which include TE insertions from unknown class (XXX) and assigned to one of the homoeolog-suppressed expression categories (**Supplementary Figures S2-S4** , **S8-S10**, **Supplementary Tables S18**, **S25**). Go terms directly related to the regulating gene expression, DNA modifications, and response to biotic and abiotic stress (cellular response to glucose stimulus (GO:0071333)gene silencing by RNA (GO:0031047), chromatin silencing (GO:0006342), DNA methylation (GO:0006306), gene silencing(GO:0016458), RNA interference (GO:0016246), response to dsRNA(GO:0043331), response to virus (GO:0009615), response to nematode (GO:0009624)) were found to be significantly enriched in the analysis performed for all the XXX insertions containing triads and missing from the results obtained from the GO SEA for triads belonging to an homoeolog-suppressed expression category that include XXX insertions (**Supplementary Figures S2-S4**, **S8-S10**, **Supplementary Tables S18**, **S25**). Meanwhile, GO terms related to aging and some abiotic stress (aging (GO:0007568), negative regulation of growth (GO:0045926), leaf senescence (GO:0010150) response to salt stress (GO:0009651), cellular response to alcohol (GO:0097306)) were found to be enriched in the list of triads belonging to one of the homoeolog-suppressed expression categories and containing XXX insertions, and missing from the GO SEA results obtained for all the XXX insertions containing triads (**Supplementary Figures S2-S4**, **S8-S10**, **Supplementary Tables S18**, **S25**).

### 3.5 TE insertions site within the gene body and triad expression patterns

Next, we tested for a possible association between TE insertion context within the gene body (exon, 5’ UTR, or 3’ UTR) of one or more of the homoeologs in a triad and homoeolog expression bias. Of the 7,439 triads that include TE insertions, 10.55% (785) include TE insertion within an exon of at least one of the genes (**Supplementary Table S3**). A lower proportion of the triads that include TE insertion within an exon was found in the balanced expression category (608, 77.45%) compared to triads that contain TE insertions but did not contain TE insertions within exons (5,776, 86.80%) with a significant difference in proportions (χ^2^ = 49.70, p < 0.001). The significant difference in proportions of the triads that include TE within an exon in balanced vs. non-balanced relative contribution categories was also observed separately for homoeolog-suppressed vs. non-suppressed (balanced and dominant relative contribution) categories (χ^2^ = 30.65, p < 0.001) and for homoeolog-dominant vs. non-dominant (balanced and suppressed relative contribution) categories (χ^2^ = 18.64, p < 0.001). Out of the triads that contained TE insertions but did not contain insertions within exons, only 11.14% (741) were found in one of the homoeolog-suppressed categories, and 2.06% (137) were found in one of the homoeolog-dominant categories, while triads that include TE insertion within an exon were more likely to be found in both the homoeolog-suppressed (17.96%, 141 triads) and the homoeolog-dominant (4.59%, 36 triads) categories.

More specifically, of the 608 triads that include TE insertions within an exon of at least one of the genes in the triad, 10.53% (64) include TE insertions within the 5’ UTR and 61.02% (371) include TE insertions within the 3’ UTR (**Table S3**). A lower proportion of the triads that include TE insertions within the 5’ UTR (46, 71.88%) and 3’ UTR (299, 80.59%) was found in the balanced expression category compared to triads that contain TE insertions but did not contain insertions within the 5’ UTRs (6,338, 85.94%) or within the 3’ UTR (6085, 86.09%), respectively. The difference in proportions of the triads that include TE insertions within the 5’ UTR (χ^2^ = 9.19, p = 0.0024 < 0.05) and within the 3’ UTR (χ^2^ = 8.31, p = 0.0039 < 0.05) in the balanced expression category relative to the proportions of triads with no TE insertions in mentioned regions, were statistically significant. We observed that triads that include TE insertions within the 5’ UTR were more likely to be found both in the homoeolog-suppressed (15.63%, 10 triads) and the homoeolog-dominant (12.50%, 8 triads) categories, relative to triads that contain TE insertions but did not contain insertions the 5’ UTRs, with only 11.82% of the triads (872) assigned to one of the homoeolog-suppressed categories and 2.24% of the triads (165) assigned to one of the homoeolog-dominant categories. Similar results were observed for triads which include TE insertions within the 3’ UTR, which were also found in higher proportions in the homoeolog-suppressed (15.09%, 56 triads) and the homoeolog-dominant (4.31%, 16 triads) categories, in comparison to triads that contain TE insertions but did not contain insertions the 3’ UTRs, for which only 11.69% of the triads (826) were assigned to one of the homoeolog-suppressed categories and only 2.22% of the triads (157) were assigned to one of the homoeolog-dominant categories. While a significant difference was observed for the proportions of triads which include TE insertions within the UTRs and triads that contain TE insertions but did not contain insertions in the UTRs within homoeolog-dominant categories (for 5’ UTR: χ^2^ = 25.08, p < 0.001 and for 3’ UTR: χ2 = 5.90, p = 0.0152 < 0.05), the difference in proportion in the homoeolog-suppressed categories were not statistically significant (for 5’ UTR: χ^2^ = 0.55, p = 0.46 > 0.05 and for 3’ UTR: χ^2^ = 3.60, p = 0.058 > 0.05).

## 4 Discussion

As an allohexaploid species, the bread wheat contains three subgenomes, A, B, and D, which originated from three diploid genome donors that diverged from a common progenitor ~7 MYA (Million Years Ago) (Levy and Feldman, 2022). While high conservation in gene content and order was observed between the three subgenomes, almost no sequence conservation was found in the intergenic regions, containing mostly TEs (Appels et al., 2018; Wicker et al., 2018). The contribution of TEs to the differentiation between the three subgenomes of the young allohexaploid bread wheat might facilitate genetic and cytological diploidization, which is essential for the survival of the new species.

### 4.1 TE insertions are highly abundant within gene bodies

Together, dyads (11.7%), triads (51.1%), and tetrads (2.8%) are accounted for 65.6% of all bread wheat HC genes, while the rest of the genes deviate from these rations (Juery et al., 2020). While triads genes were kept in a 1:1:1 ratio between the three bread wheat subgenomes, dyads and tetrads homoeologous groups underwent copy number variations during the wheat group evolution (Juery et al., 2020). A study by Juery et al. (2020) showed that triads are diverse from dyads and tetrads in various characteristics, including conservation, chromosomal distribution, epigenetic modification, gene ontology, and expression patterns. Their findings led Juery et al. (2020) to suggest that the highly conserved triads belong to the bread wheat core genome, while dyads and tetrads are parts of the dispensable genome.

To address the possible effect of TE insertions within gene bodies on gene expression, we first identified TE insertions within dyads, triads, and tetrads genes. We found that a high percentage of the examined genes contained TE insertions within exons and introns, with the highest proportions of TE insertions found in triads genes. Additionally, genes found in the proximal region were more likely to include TE insertions within the gene body, suggesting that TE distribution within the gene body is in accordance with TE distribution across the chromosome, with lower density in the distal regions. However, the higher percentage of TE insertions in triads genes relative to dyads and tetrads genes persist throughout the different chromosomal regions. Therefore, the difference in the abundance of TE insertions within gene bodies between triads and dyads and tetrads genes is not only due to the higher abundance of triads genes in the TE rich proximal regions.

The higher abundance of TE insertions within triads genes relative to dyads and tetrads genes might be attributed to some of the distinguish characteristics of each of the categories. For instance, triads genes were found to be enriched in the H3K9ac active euchromatin mark and expressed at a higher level and higher breadth relative to dyads and tetrads genes, which were more associated with repressive H3K27me3 modification (Juery et al., 2020). There is evidence that some TEs are preferentially inserted into transcriptionally active regions near active histone marks (Bennetzen, 2000; Hirsch and Springer, 2017; Sultana et al., 2017), conditions that fit better to triads genes. Specifically, TEs belonging to the *Mariner* superfamily (DTT), the most abundant superfamily in triad genes, are known to be enriched in genes with high expression (Sultana et al., 2017). Moreover, the higher conservation of triads genes might contribute to the persistence of the TE insertion, provided that the insertion did not result in loss of fitness. Alternatively, the presence of TE insertion within the gene body might impact various characteristics of the gene and maybe ultimately on gene conservation. Further study is necessary for a better understanding of the processes leading to the unique TE distribution pattern observed in this study.

### 4.2 Polymorphic TE insertions within gene bodies associated with non-balanced expression within the homoeologous group

Since the three diploid genome donors of bread wheat originated from a common ancestor, it is not surprising that a high percentage of wheat HC genes are conserved and syntenic between the three subgenomes (Appels et al., 2018). Similarly, the abundances of 76% of TEs families were found to be similar between the A, B, and D sub-genomes of bread wheat and TE families distribution in promoter regions was found to be highly conserved between subgenomes (Wicker et al., 2018). However, almost no conserved TE insertions were observed between the three subgenomes, and more specifically, no conservation of TE insertions was observed between homeologous promoters (Wicker et al., 2018).

Here, we assigned homoeologous groups as monomorphic or polymorphic based only on the presence of TE insertions within all or only some of the gene bodies of the homoeologs in the group. While the TE insertions found within the homoeologs in a monomorphic group were not necessarily inherited from the common ancestor, did not necessarily belong to the same TE family, and might have been inserted in different locations in the sequence, in this part of our analysis we focused on the effect of the mere presence of TE insertion on the relative expression within the homoeologous group. However, the fact that the proportion of polymorphic groups did not match a module describing the random distribution of presence\ absence of TE insertions leads us to suggest that some of the TE insertions are indeed having a common origin, or alternatively, that common characteristics of the homoeologs affected the probability of TEs to insert into each one of the genes in the homoeologous group. The percentage of polymorphic groups was lower than expected for triads and tetrads and higher than expected for dyads, which were found to be the least conserved relative to triads and tetrads (Juery et al., 2020). Additionally, our analysis revealed a strong significant correlation between polymorphic TE insertions and non-balanced expression patterns of triads. We suggest that the mentioned correlation might be a result of either the effect of TE insertions on gene expression and \ or TE target site preference influenced by gene expression patterns and expression breadth.

### 4.3 Strong association between TE insertions within gene bodies and homoeolog expression bias

Since TE insertions were found to be abundant in triads, and a clear correlation was observed between TE insertion pattern and relative expression of the homoeolog within the triad, we focused on triads to further learn about the possible impact of TE insertions on gene expression, using existing data regarding the relative contribution of each homoeolog to the overall triad expression. Our analysis revealed that a great variety of TEs inserted within wheat gene bodies, both into introns and exons. Here, we observed a strong correlation between the presence of TE insertions in gene bodies and the balanced expression of the three homoeologs in the triad. Similar to the differences in TE abundant in triads vs. dyads and tetrads genes, the unique characteristics of each relative expression category might explain the difference in TE content. Syntenic triads that were classified as balanced showed higher expression levels and had higher levels of active histone markers than syntenic triads in the homoeolog-dominant and homoeolog-suppressed categories (Ramírez-González et al., 2018). As we suggested for triads vs. dyads and tetrads, those characteristics, together with the balanced triads over representation in low recombination regions (Ramírez-González et al., 2018), might lid to higher insertion rate and higher persistent of TE insertions in the balanced triads genes relative to triads from the non-balanced categories. This claim is supported by the very high abundant of insertions from the *Mariner* superfamily (DTT), which was found to be enriched in genes with high expression (Sultana et al., 2017), in balanced triads and by the strong correlation observed specifically between the presence of TE insertions from the DTT superfamily and balanced homoeologs expression. Additionally, we suggest that the presence of TE insertions within gene bodies might result in a change in gene expression, resulting in balanced expression of the homoeologs.

Generally, a strong correlation was observed between the presence of TE insertions within the triad and balanced expression. However, while considering the insertion site and TE type, a more complex relationship between the presence of TE insertion and homoeolog expression bias is revealed. We found that specific TE superfamilies and families were enriched in triads which showed specific relative expression patterns. Furthermore, the presence of TE insertions from 13 out of the 14 TE superfamilies within a triad associated with multiple GO terms enriched both in basic cellular functions and in response to environmental factors. Triads that contained TE insertions from each one of the 13 different TE superfamilies showed enrichment for a unique set of GO terms. Triads which include insertions of DTT and DTX, superfamilies for which a positive correlation was identified between TE presence within gene bodies and balanced expression of the triad, are found in association with numerous GO terms related to basic cell processes (**Figure 2A**). Additionally, triads which include insertions belonging to TE superfamilies that showed a positive correlation between TE presence within gene bodies and suppressed or dominant relative expression of the homoeologs (DTM, RLX, RLC, and XXX) were enriched for multiple GO terms. Specifically, triads that contained TE insertions from each of those 4 superfamilies (DTM, RLX, RLC, and XXX) were enriched for GO terms related to response to biotic and \ or abiotic stimulus (**Figures 2B, C**).

## 5 Conclusions

In this study, the integration and analysis of data from several publicly available databases revealed significant correlations between the presence of TE insertions within gene bodies, gene expression and gene function in a genome-specific manner in wheat. We found that TE insertion site within the gene (exon\ intron) and TE type (superfamily\ subfamily) correlate strongly with homoeolog expression bias. Additionally, presence of TE insertion from all tested TE superfamilies were found to associate with numerous gene functions. Future studies are needed to decipher the causes for such correlations. In addition, comparative analysis between bread wheat accessions might shed light on the evolutionary time frame for TE insertions into gene bodies and on the involved mechanisms connecting TE presence within the gene body, gene expression, and gene function.

## Resource identification initiative

SciPy (RRID : SCR_008058)

Pandas (RRID : SCR_018214)

statsmodel (RRID : SCR_016074)

agriGO (RRID : SCR_006989)

REViGO (RRID : SCR_005825)

## Data availability statement

The original contributions presented in the study are included in the article/**Supplementary Material**. Further inquiries can be directed to the corresponding author.

## Author contributions

IB: generated data, data analysis, wrote manuscript. LG: data analysis, wrote manuscript. KK: data analysis, wrote and edit the manuscript, corresponding author, submitted manuscript. All authors contributed to the article and approved the submitted version.
