# Supplementary material for: Transposable elements are associated with genome-specific gene expression in bread wheat
Source: Front Plant Sci. 2023 Jan 12;13:1072232. doi: 10.3389/fpls.2022.1072232 (PMC9878150; doi:10.3389/fpls.2022.1072232)
Supplement: Supplementary file 1 [file DataSheet_1.pdf]

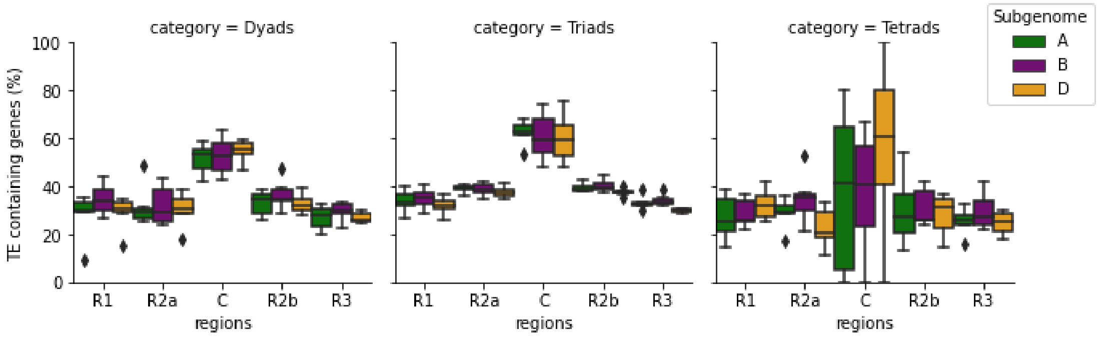

Figure S1: Percentage of TE containing genes belonging to the dyads, triads, or tetrads categories in the five chromosomal regions (R1, R2a, C, R2b, and R3) in each of the three bread wheat subgenomes. Gene distribution was calculated individually for genes that belong to the same category and found in the same and chromosomal region in each one of 18 bread wheat chromosomes. Genes found in chromosomes 1A, 1B, and 1D were eliminated from the analysis due to the lack of tetrads genes in the centromeres of chromosomes 1A and 1B. The boxplots depict the first quartile (Q1) and the third quartile (Q3) of the data with the median between them. The whiskers extend from the box to 1.5x the interquartile range (IQR). Rhombuses represent values found past the end of the whiskers. boxplots were drawn using the *boxplot* function from the seaborn python package.

A –

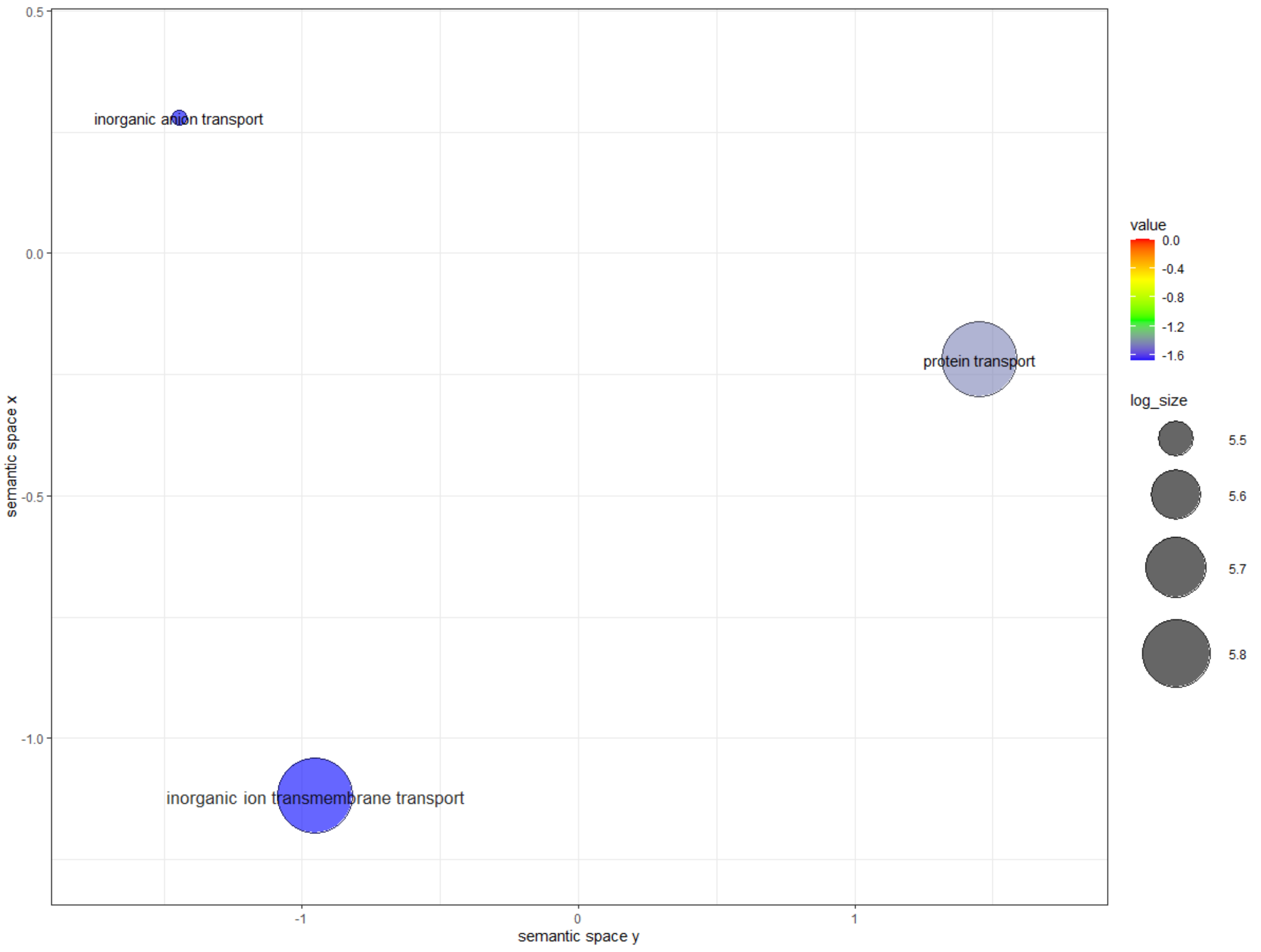

inorganic anion transport
protein transport
inorganic ion transmembrane transport
value
0.0
-0.4
-0.8
-1.2
-1.6
log_size
5.5
5.6
5.7
5.8
0.5
0.0
-0.5
-1.0
semantic space x
-1
0
1
semantic space y

B –

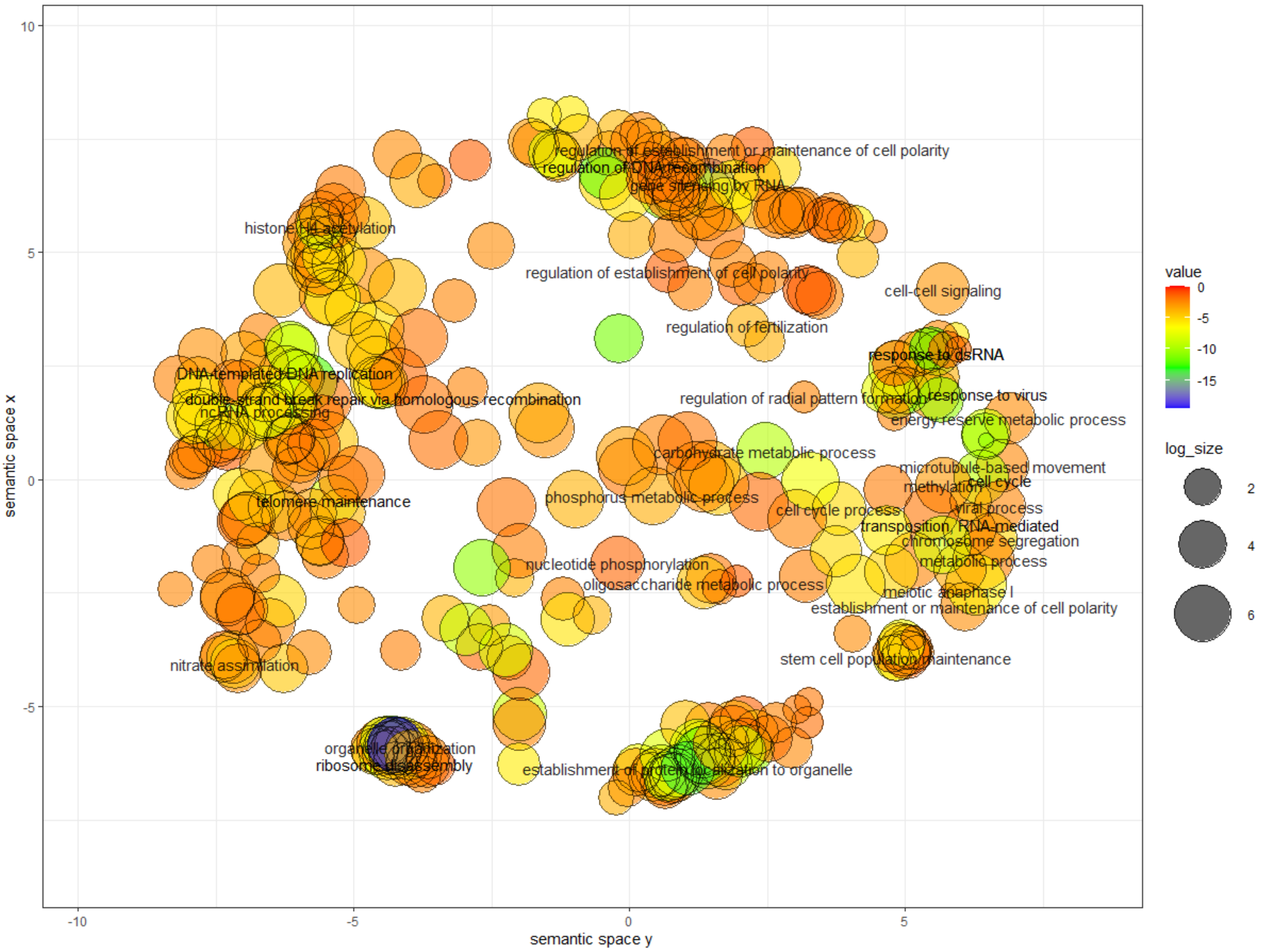
regulation of establishment or maintenance of cell polarity
regulation of DNA recombination
gene silencing by RNA
histone H4 acetylation
regulation of establishment of cell polarity
cell-cell signaling
regulation of fertilization
response to dsRNA
DNA-templated DNA replication
double-strand break repair via homologous recombination
ncRNA processing
regulation of radial pattern formation
response to virus
energy reserve metabolic process
carbohydrate metabolic process
microtubule-based movement
methylation
cell cycle
telomere maintenance
phosphorus metabolic process
cell cycle process
viral process
transposition, RNA-mediated
chromosome segregation
metabolic process
nucleotide phosphorylation
oligosaccharide metabolic process
meiotic anaphase I
establishment or maintenance of cell polarity
stem cell population maintenance
nitrate assimilation
organelle organization
ribosome disassembly
establishment of protein localization to organelle
semantic space x
semantic space y
value
log_size
10
5
0
-5
-10
-15
2
4
6

C –

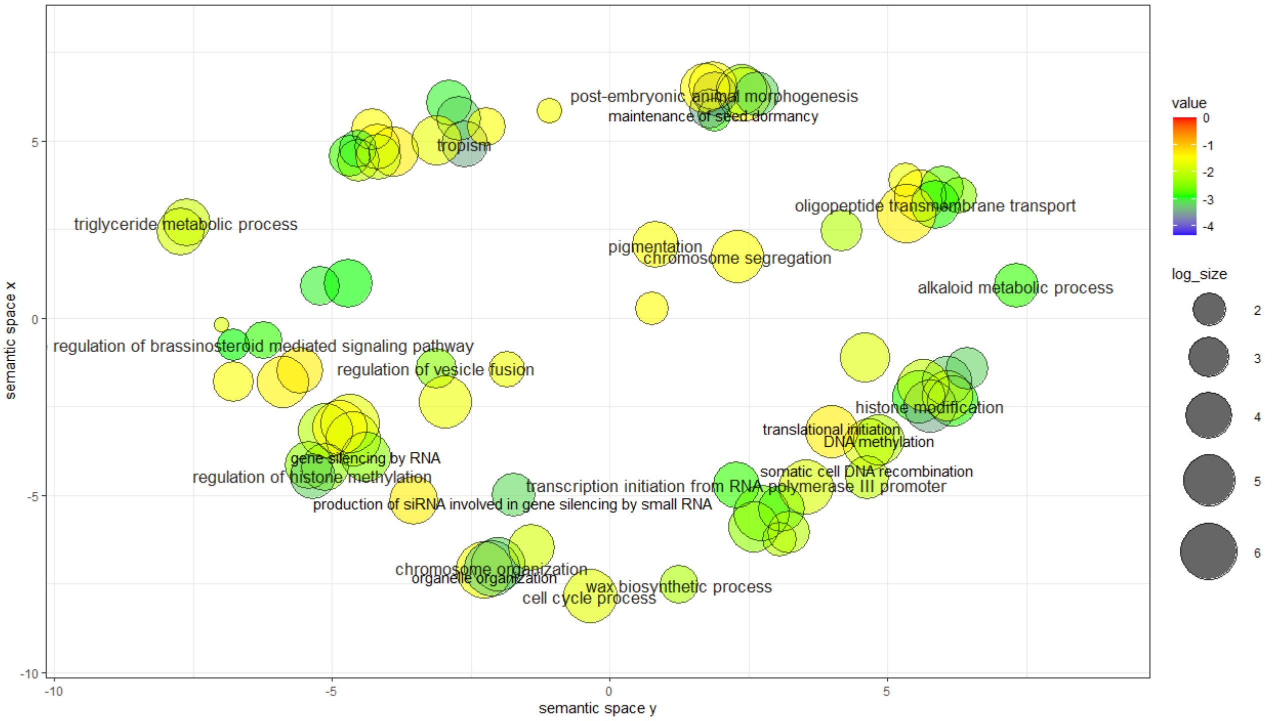
post-embryonic animal morphogenesis
maintenance of seed dormancy
tropism
oligopeptide transmembrane transport
triglyceride metabolic process
pigmentation
chromosome segregation
alkaloid metabolic process
regulation of brassinosteroid mediated signaling pathway
regulation of vesicle fusion
histone modification
translational initiation
DNA methylation
gene silencing by RNA
regulation of histone methylation
somatic cell DNA recombination
transcription initiation from RNA polymerase III promoter
production of siRNA involved in gene silencing by small RNA
chromosome organization
organelle organization
wax biosynthetic process
cell cycle process
semantic space x
semantic space y
value
log_size

D –

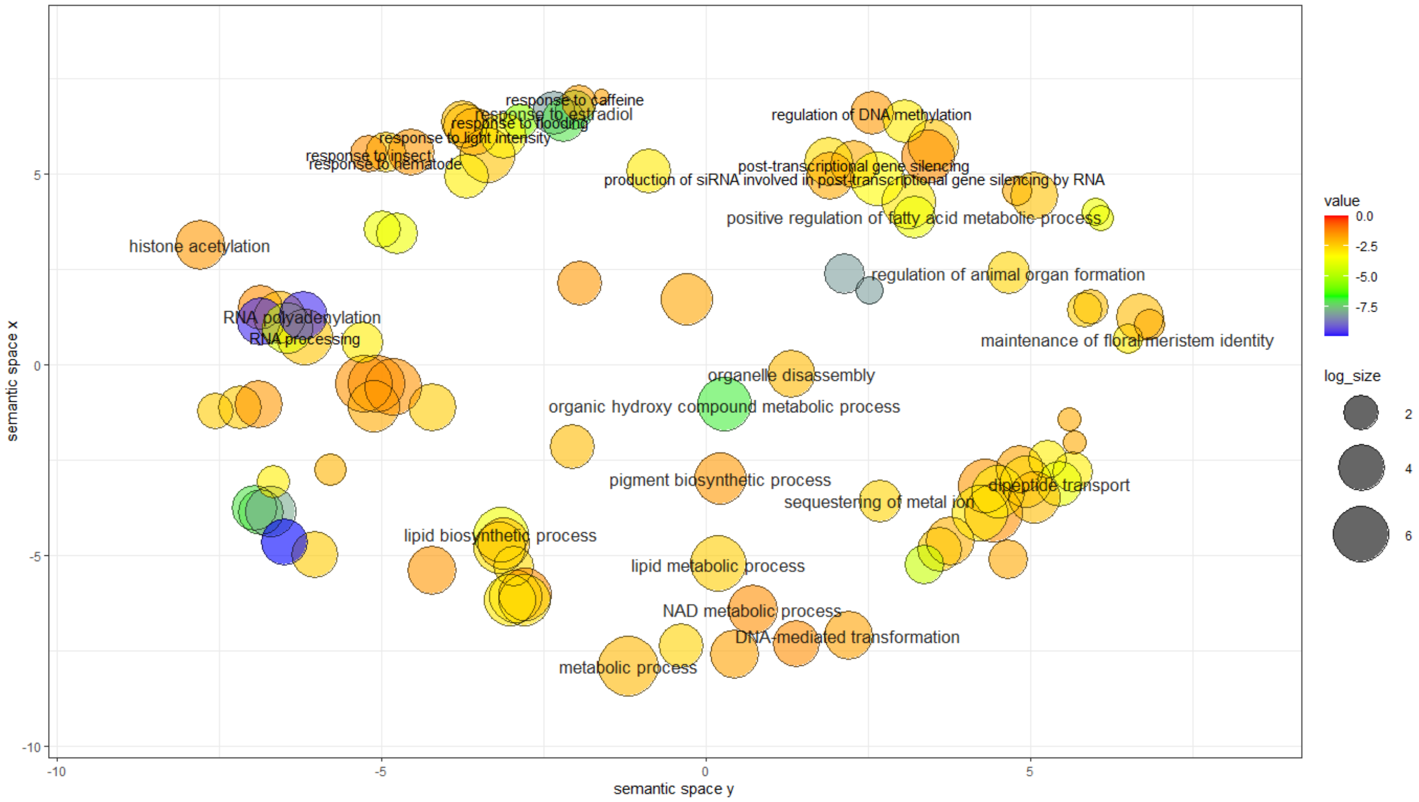
response to caffeine
response to estradiol
response to flooding
response to light intensity
response to insect
response to nematode
regulation of DNA methylation
post-transcriptional gene silencing
production of siRNA involved in post-transcriptional gene silencing by RNA
positive regulation of fatty acid metabolic process
histone acetylation
regulation of animal organ formation
RNA polyadenylation
RNA processing
maintenance of floral meristem identity
organelle disassembly
organic hydroxy compound metabolic process
pigment biosynthetic process
sequestering of metal ion
dipeptide transport
lipid biosynthetic process
lipid metabolic process
NAD metabolic process
DNA-mediated transformation
metabolic process
semantic space x
semantic space y
value
log_size

E –

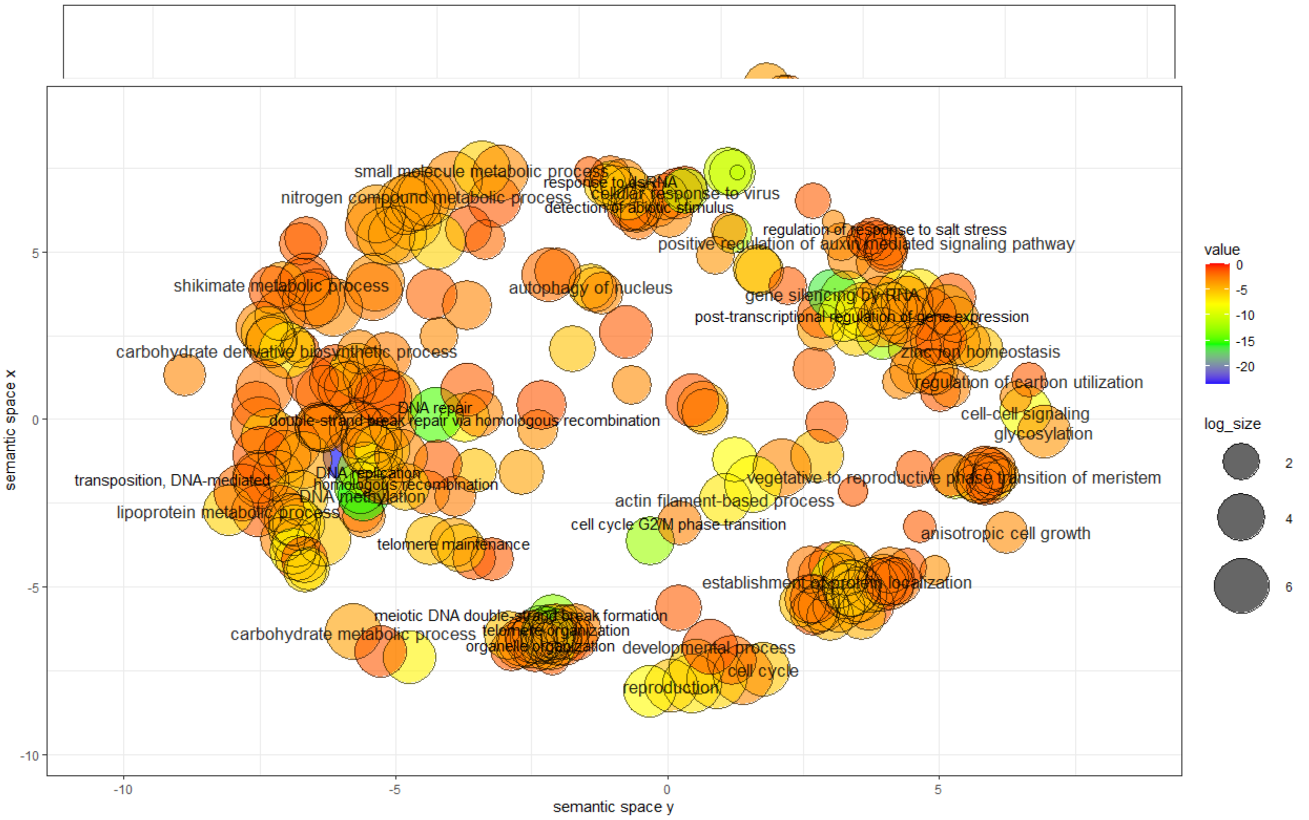
small molecule metabolic process
nitrogen compound metabolic process
response to dsRNA
cellular response to virus
detection of abiotic stimulus
regulation of response to salt stress
positive regulation of auxin mediated signaling pathway
shikimate metabolic process
autophagy of nucleus
gene silencing by RNA
post-transcriptional regulation of gene expression
carbohydrate derivative biosynthetic process
zinc ion homeostasis
regulation of carbon utilization
DNA repair
double-strand break repair via homologous recombination
cell-cell signaling
glycosylation
transposition, DNA-mediated
DNA replication
homologous recombination
DNA methylation
vegetative to reproductive phase transition of meristem
actin filament-based process
lipoprotein metabolic process
cell cycle G2/M phase transition
anisotropic cell growth
telomere maintenance
establishment of protein localization
meiotic DNA double-strand break formation
carbohydrate metabolic process
telomere organization
organelle organization
developmental process
cell cycle
reproduction
value
0
-5
-10
-15
-20
log_size
2
4
6
semantic space x
semantic space y
5
0
-5
-10
-10
-5
0
5

F –

G –

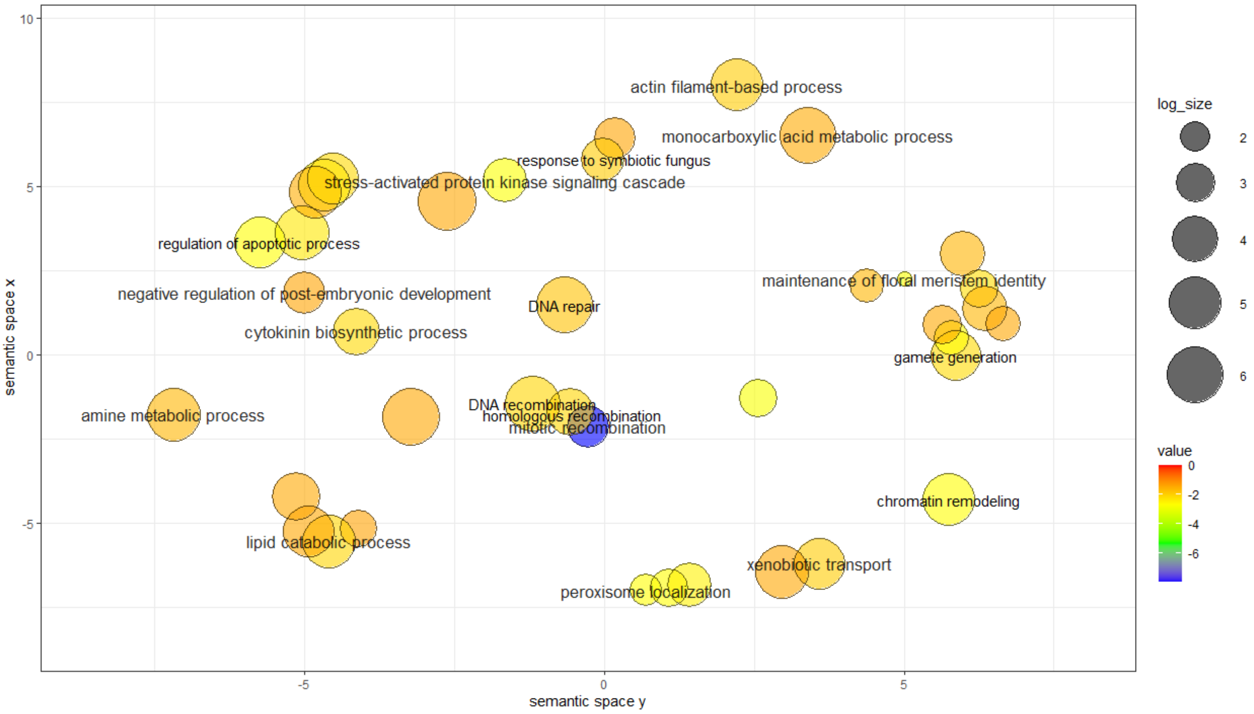
actin filament-based process
monocarboxylic acid metabolic process
response to symbiotic fungus
stress-activated protein kinase signaling cascade
regulation of apoptotic process
maintenance of floral meristem identity
negative regulation of post-embryonic development
DNA repair
cytokinin biosynthetic process
gamete generation
amine metabolic process
DNA recombination
homologous recombination
mitotic recombination
chromatin remodeling
lipid catabolic process
xenobiotic transport
peroxisome localization
log_size
2
3
4
5
6
value
0
-2
-4
-6
semantic space x
semantic space y
10
5
0
-5
-5
0
5

H –

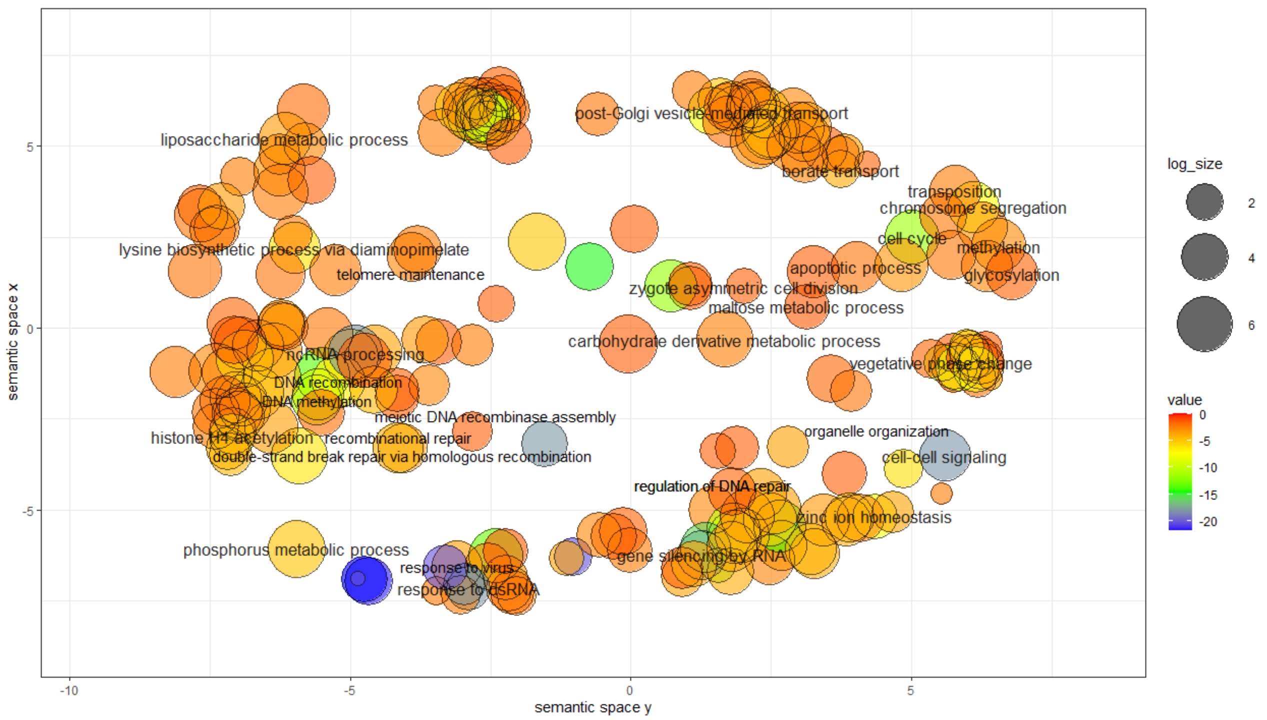
post-Golgi vesicle-mediated transport
liposaccharide metabolic process
borate transport
transposition
chromosome segregation
cell cycle
methylation
glycosylation
lysine biosynthetic process via diaminopimelate
telomere maintenance
apoptotic process
zygote asymmetric cell division
maltose metabolic process
carbohydrate derivative metabolic process
vegetative phase change
ncRNA processing
DNA recombination
DNA methylation
meiotic DNA recombinase assembly
histone H4 acetylation
recombinational repair
double-strand break repair via homologous recombination
organelle organization
cell-cell signaling
regulation of DNA repair
zinc ion homeostasis
phosphorus metabolic process
gene silencing by RNA
response to virus
response to dsRNA
semantic space x
semantic space y
log_size
value

I –

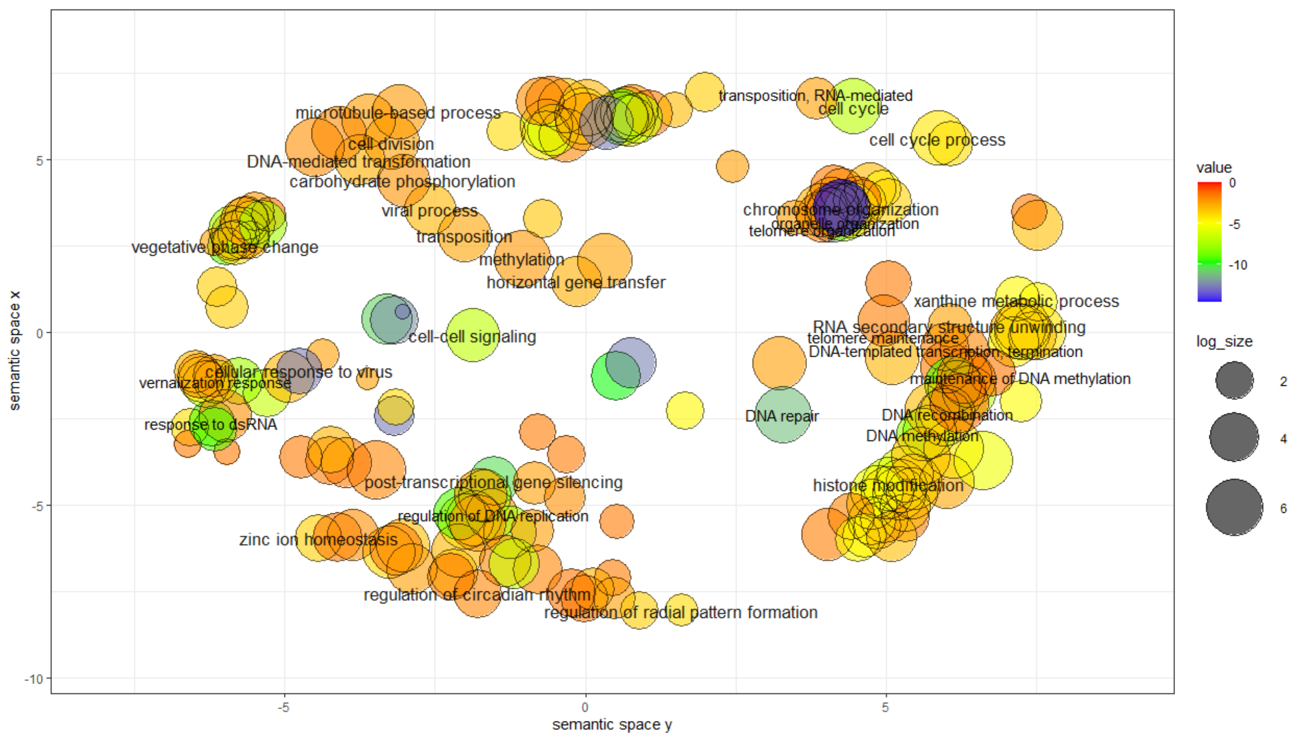
transposition, RNA-mediated
cell cycle
microtubule-based process
cell division
cell cycle process
DNA-mediated transformation
carbohydrate phosphorylation
viral process
chromosome organization
telomere organization
organelle organization
transposition
vegetative phase change
methylation
horizontal gene transfer
xanthine metabolic process
RNA secondary structure unwinding
telomere maintenance
DNA-templated transcription, termination
cell-cell signaling
cellular response to virus
vernalization response
maintenance of DNA methylation
response to dsRNA
DNA repair
DNA recombination
DNA methylation
post-transcriptional gene silencing
histone modification
regulation of DNA replication
zinc ion homeostasis
regulation of circadian rhythm
regulation of radial pattern formation
semantic space x
semantic space y
value
log_size

J –

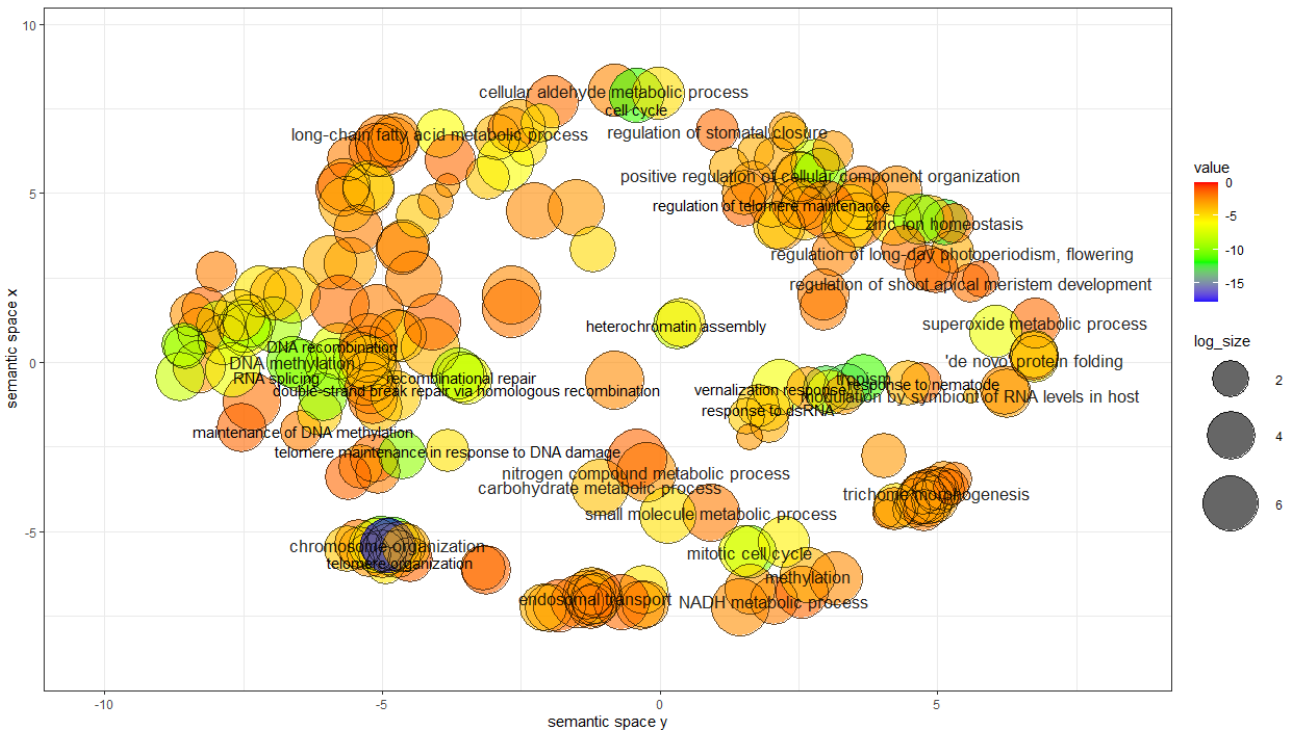

cellular aldehyde metabolic process
cell cycle
long-chain fatty acid metabolic process
regulation of stomatal closure
positive regulation of cellular component organization
regulation of telomere maintenance
zinc ion homeostasis
regulation of long-day photoperiodism, flowering
regulation of shoot apical meristem development
heterochromatin assembly
superoxide metabolic process
'de novo' protein folding
DNA recombination
DNA methylation
RNA splicing
recombinational repair
double-strand break repair via homologous recombination
vernalization response
tropism
response to nematode
modulation by symbiont of RNA levels in host
response to dsRNA
maintenance of DNA methylation
telomere maintenance in response to DNA damage
nitrogen compound metabolic process
carbohydrate metabolic process
trichome morphogenesis
small molecule metabolic process
chromosome organization
telomere organization
mitotic cell cycle
methylation
endosomal transport
NADH metabolic process
semantic space x
semantic space y
value
log_size

K –

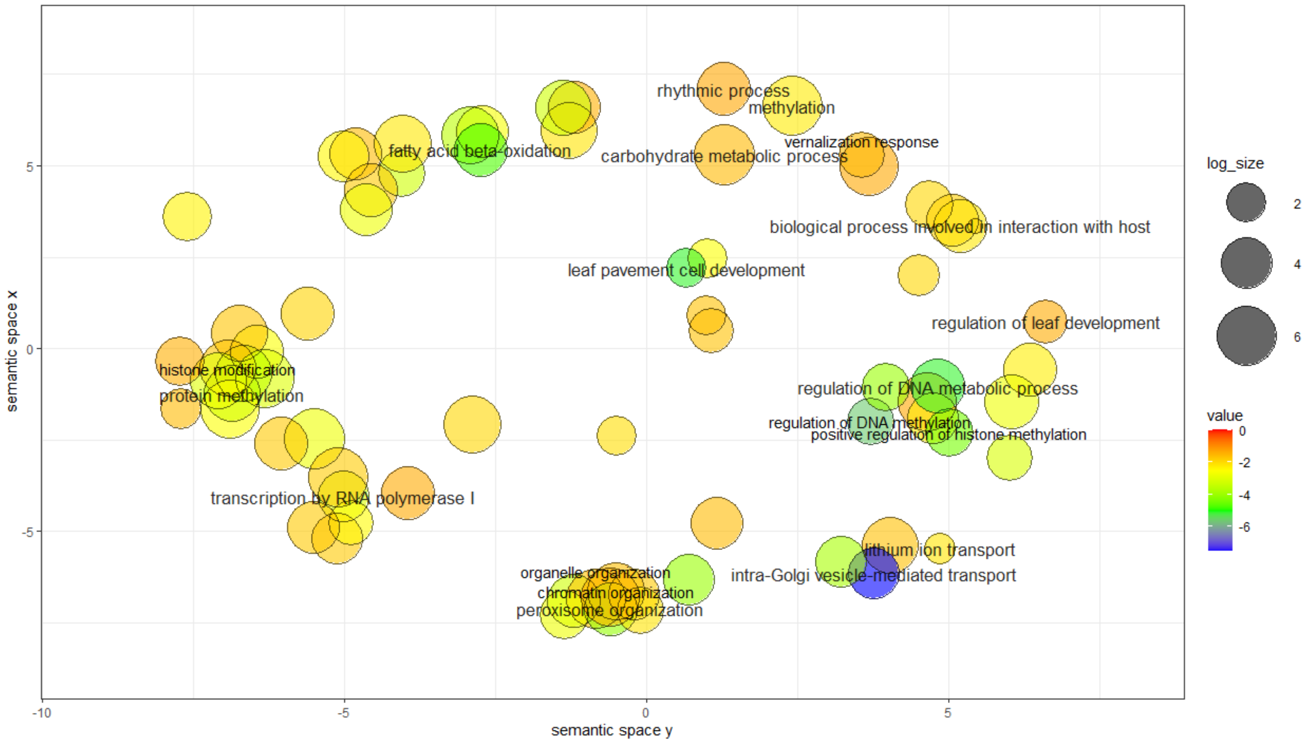

rhythmic process
methylation
fatty acid beta-oxidation
carbohydrate metabolic process
vernalization response
biological process involved in interaction with host
leaf pavement cell development
regulation of leaf development
histone modification
protein methylation
regulation of DNA metabolic process
regulation of DNA methylation
positive regulation of histone methylation
transcription by RNA polymerase I
lithium ion transport
intra-Golgi vesicle-mediated transport
organelle organization
chromatin organization
peroxisome organization
semantic space x
semantic space y
log_size
value

L –

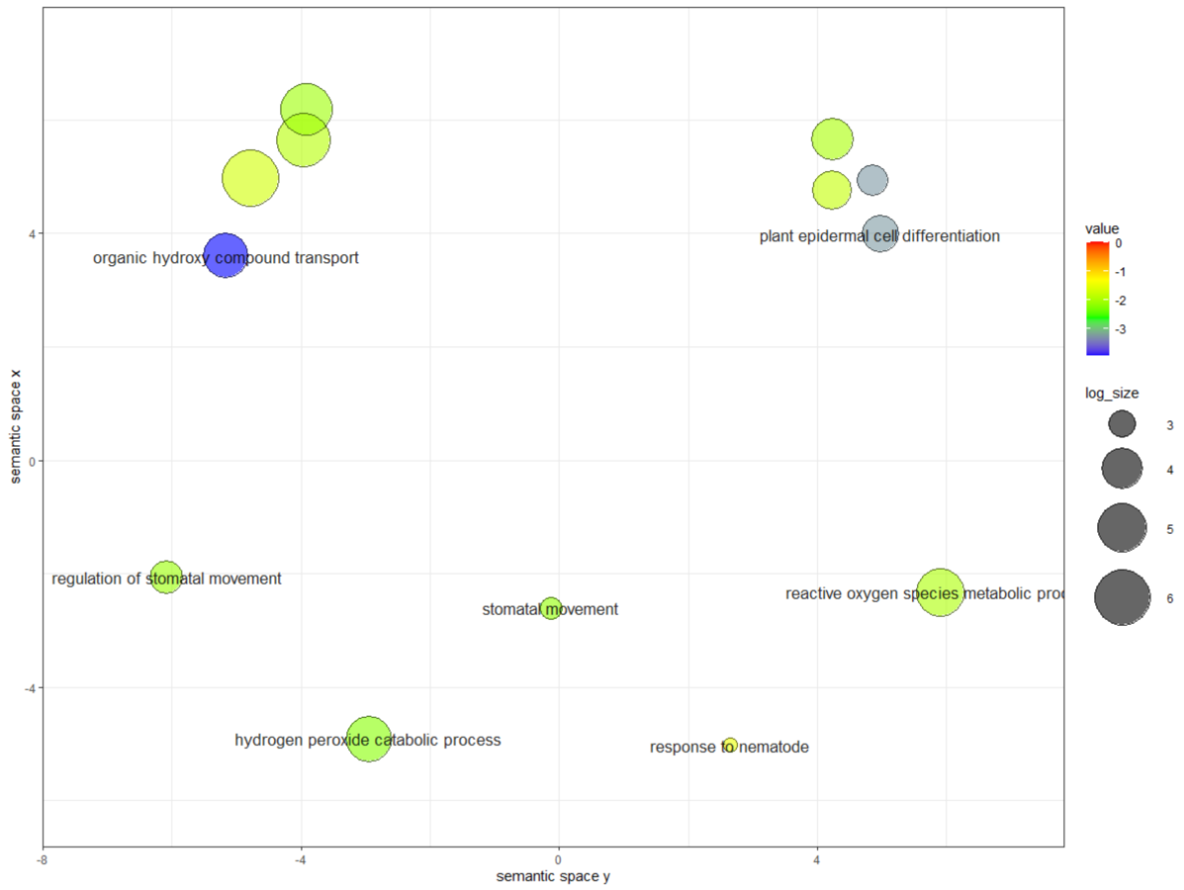

organic hydroxy compound transport
plant epidermal cell differentiation
regulation of stomatal movement
stomatal movement
reactive oxygen species metabolic proc
hydrogen peroxide catabolic process
response to nematode
semantic space x
semantic space y
value
log_size

M –

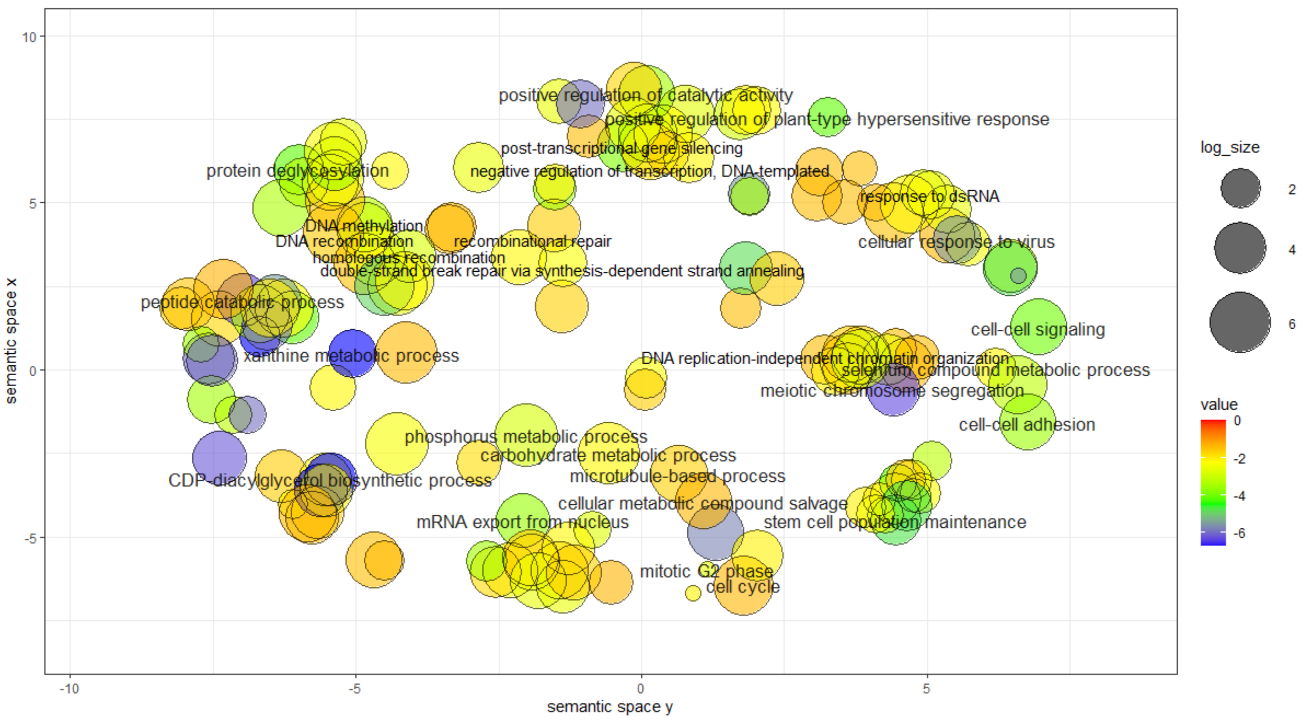

positive regulation of catalytic activity
positive regulation of plant-type hypersensitive response
post-transcriptional gene silencing
negative regulation of transcription, DNA-templated
protein deglycosylation
response to dsRNA
DNA methylation
DNA recombination
recombinational repair
homologous recombination
double-strand break repair via synthesis-dependent strand annealing
cellular response to virus
peptide catabolic process
cell-cell signaling
xanthine metabolic process
DNA replication-independent chromatin organization
selenium compound metabolic process
meiotic chromosome segregation
cell-cell adhesion
phosphorus metabolic process
carbohydrate metabolic process
microtubule-based process
CDP-diacylglycerol biosynthetic process
cellular metabolic compound salvage
mRNA export from nucleus
stem cell population maintenance
mitotic G2 phase
cell cycle
semantic space x
semantic space y
log_size
value

Figure S2 – Enriched terms for BP category for all triads with all superfamilies A-DTA, B-DTC, C-DTH, D-DTM, E-DTT, F-DTX, G-DXX, H-RIX, I-RLC, J-RLG, K-RLX, L-SIX, M-XXX. The scatterplots were generated by REVIGO , bubble color indicates the FDR value and the size indicates the frequency of the GO term in the Gene Ontology Annotation (GOA) database, bubbles of more general terms are larger.

A –

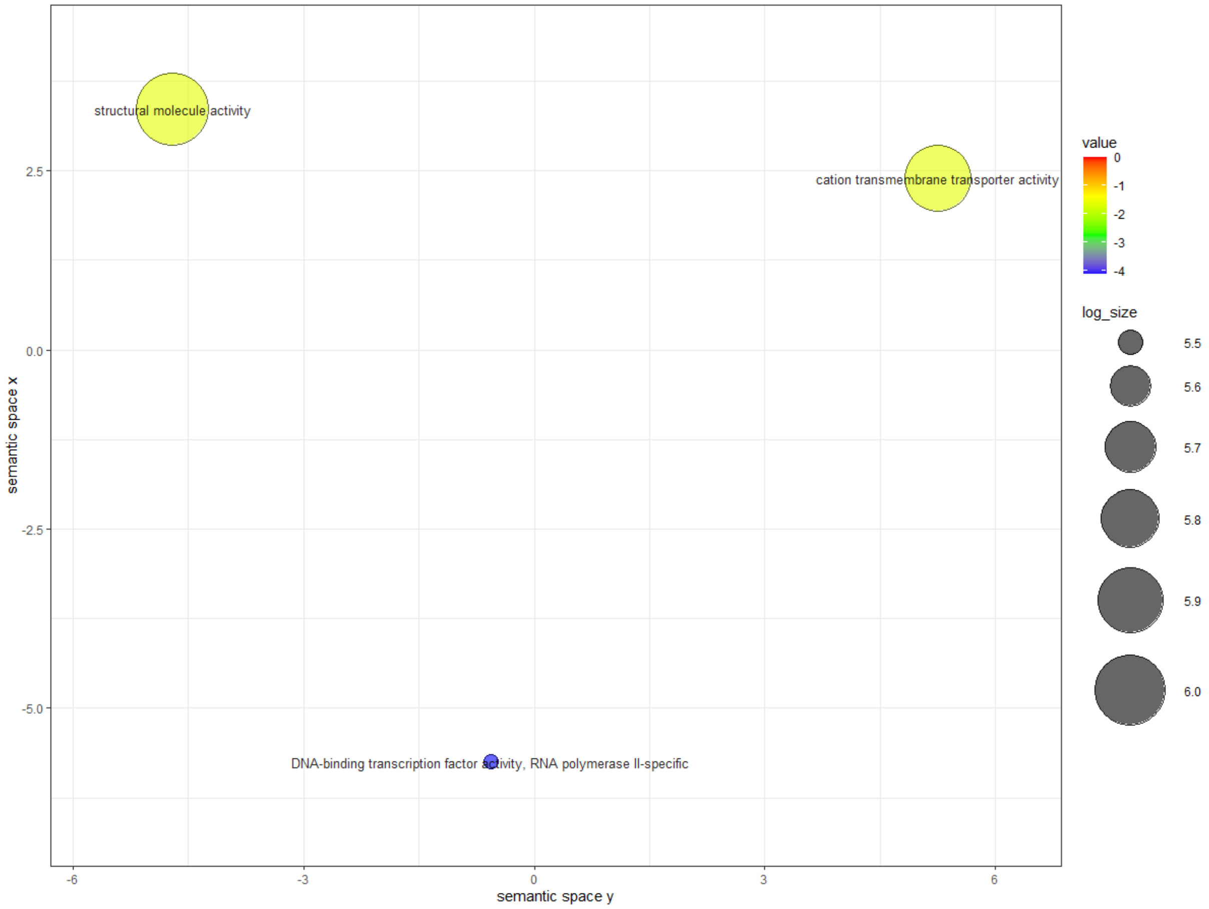

B –

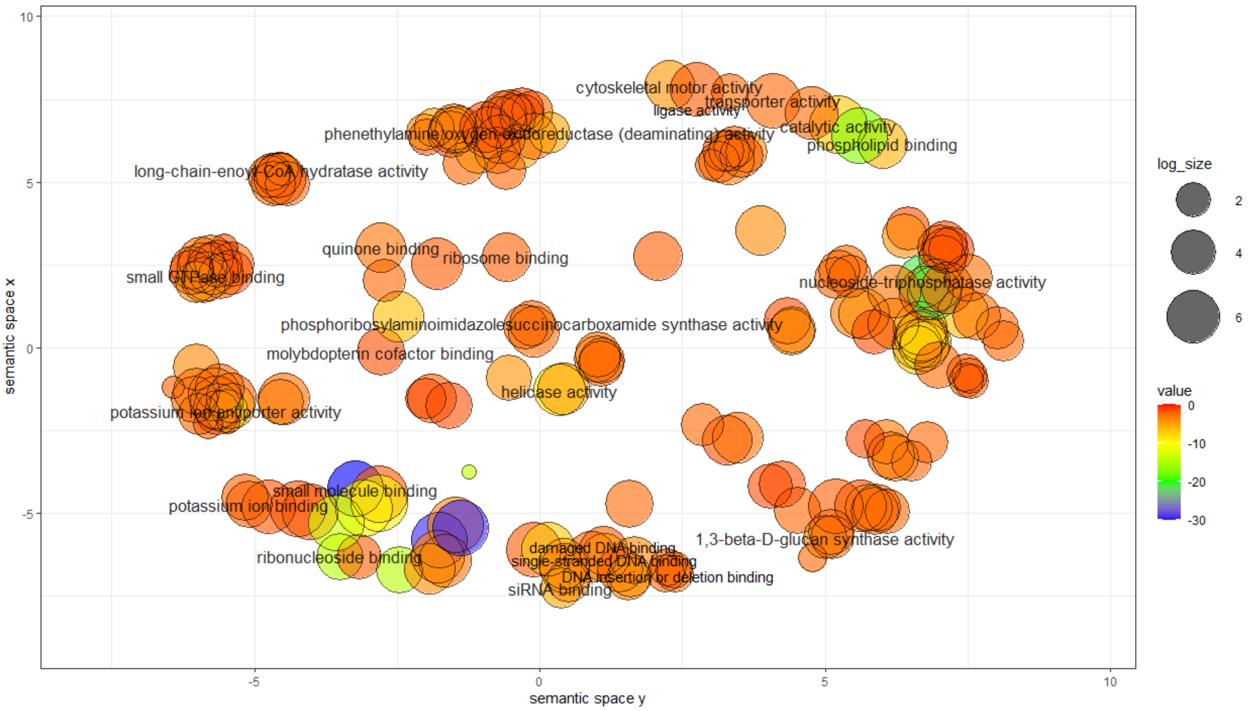

C –

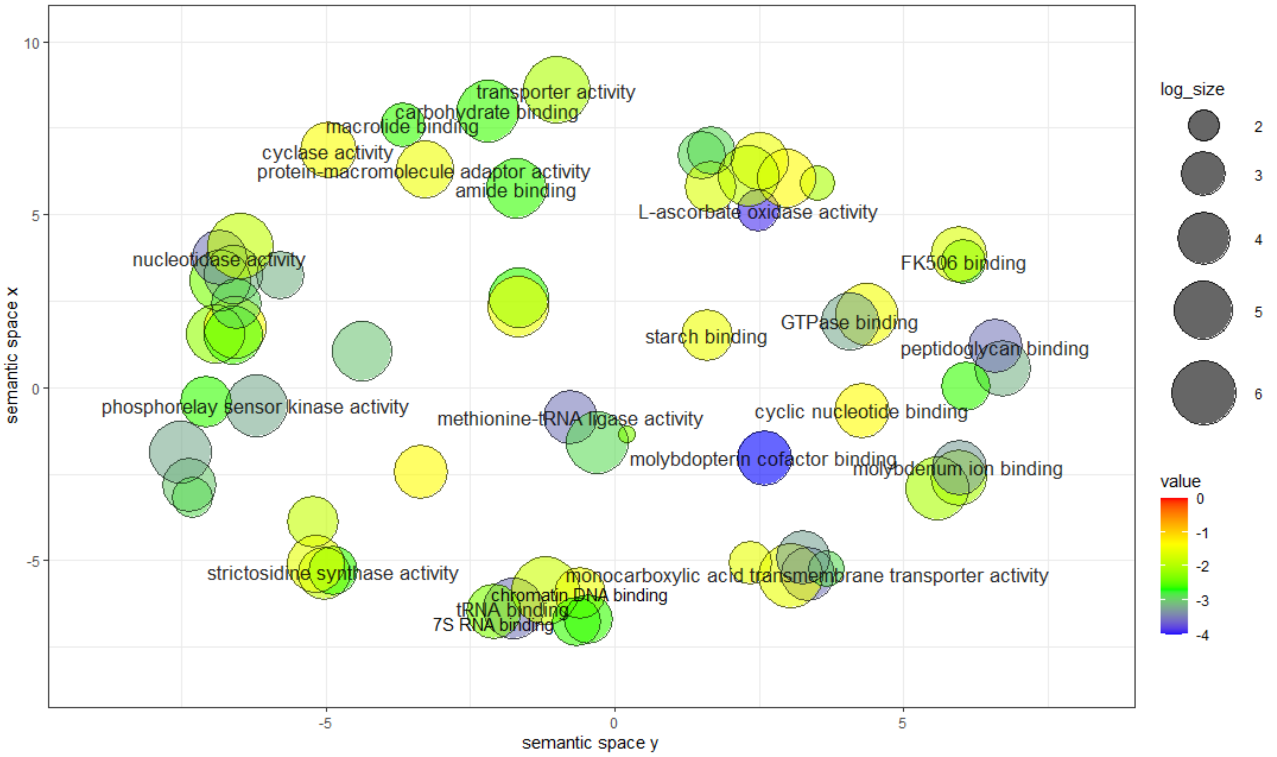
transporter activity
carbohydrate binding
macrolide binding
cyclase activity
protein-macromolecule adaptor activity
amide binding
L-ascorbate oxidase activity
nucleotidase activity
FK506 binding
GTPase binding
starch binding
peptidoglycan binding
phosphorelay sensor kinase activity
methionine-tRNA ligase activity
cyclic nucleotide binding
molybdopterin cofactor binding
molybdenum ion binding
strictosidine synthase activity
monocarboxylic acid transmembrane transporter activity
chromatin DNA binding
tRNA binding
7S RNA binding
semantic space x
semantic space y
10
5
0
-5
log_size
2
3
4
5
6
value
0
-1
-2
-3
-4

D –

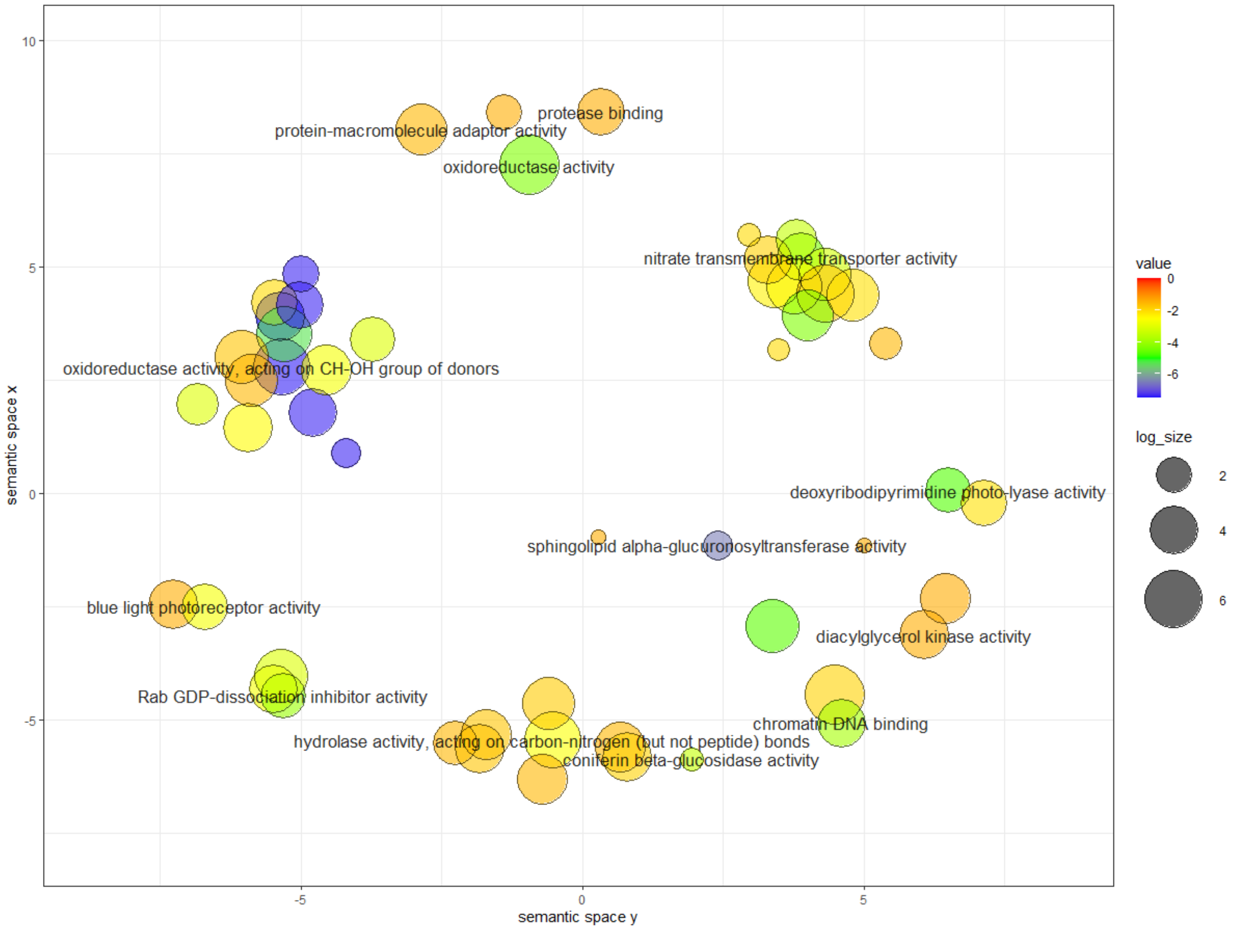
protease binding
protein-macromolecule adaptor activity
oxidoreductase activity
nitrate transmembrane transporter activity
oxidoreductase activity, acting on CH-OH group of donors
deoxyribodipyrimidine photo-lyase activity
sphingolipid alpha-glucuronosyltransferase activity
blue light photoreceptor activity
diacylglycerol kinase activity
Rab GDP-dissociation inhibitor activity
chromatin DNA binding
hydrolase activity, acting on carbon-nitrogen (but not peptide) bonds
coniferin beta-glucosidase activity
semantic space x
semantic space y
10
5
0
-5
value
0
-2
-4
-6
log_size
2
4
6

E –

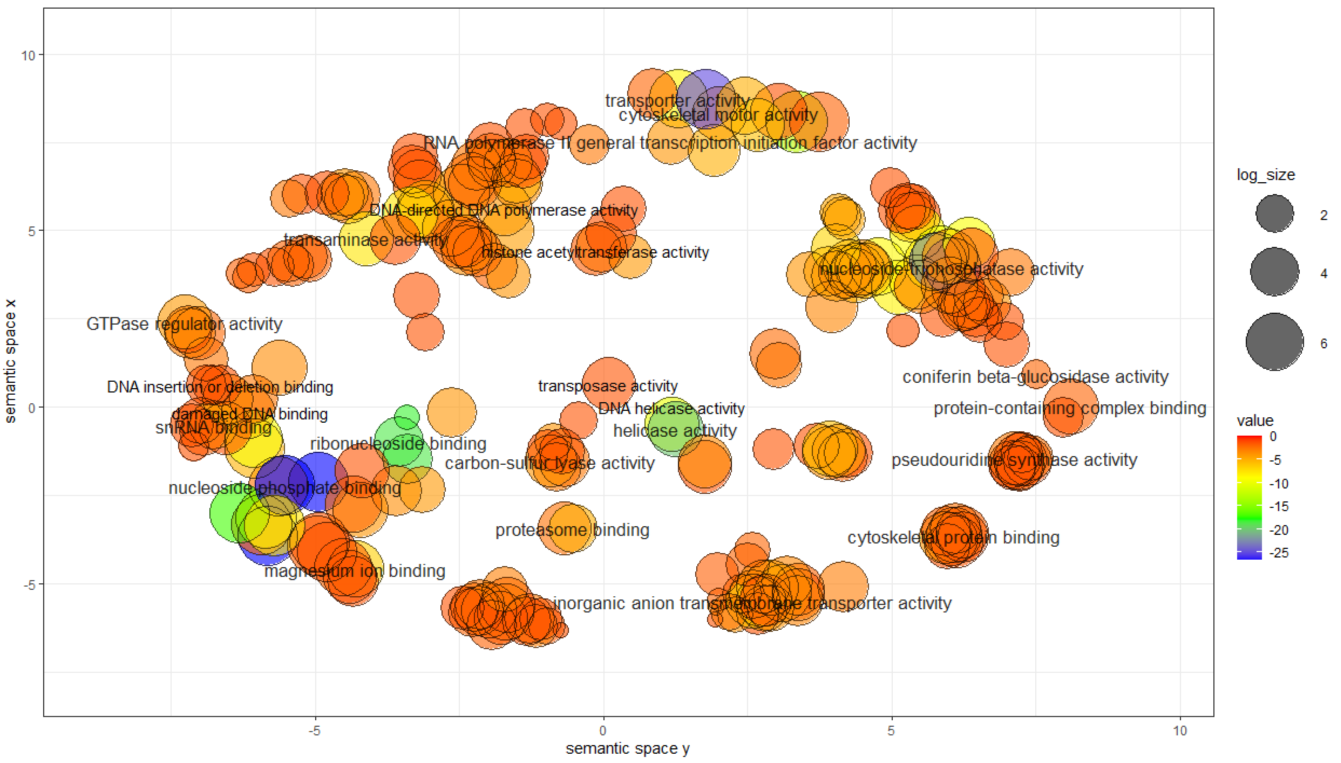
transporter activity
cytoskeletal motor activity
RNA polymerase II general transcription initiation factor activity
DNA-directed DNA polymerase activity
transaminase activity
histone acetyltransferase activity
nucleoside-triphosphatase activity
GTPase regulator activity
coniferin beta-glucosidase activity
DNA insertion or deletion binding
transposase activity
protein-containing complex binding
damaged DNA binding
DNA helicase activity
snRNA binding
helicase activity
ribonucleoside binding
carbon-sulfur lyase activity
pseudouridine synthase activity
nucleoside phosphate binding
proteasome binding
cytoskeletal protein binding
magnesium ion binding
inorganic anion transmembrane transporter activity
semantic space x
semantic space y
log_size
value

F –

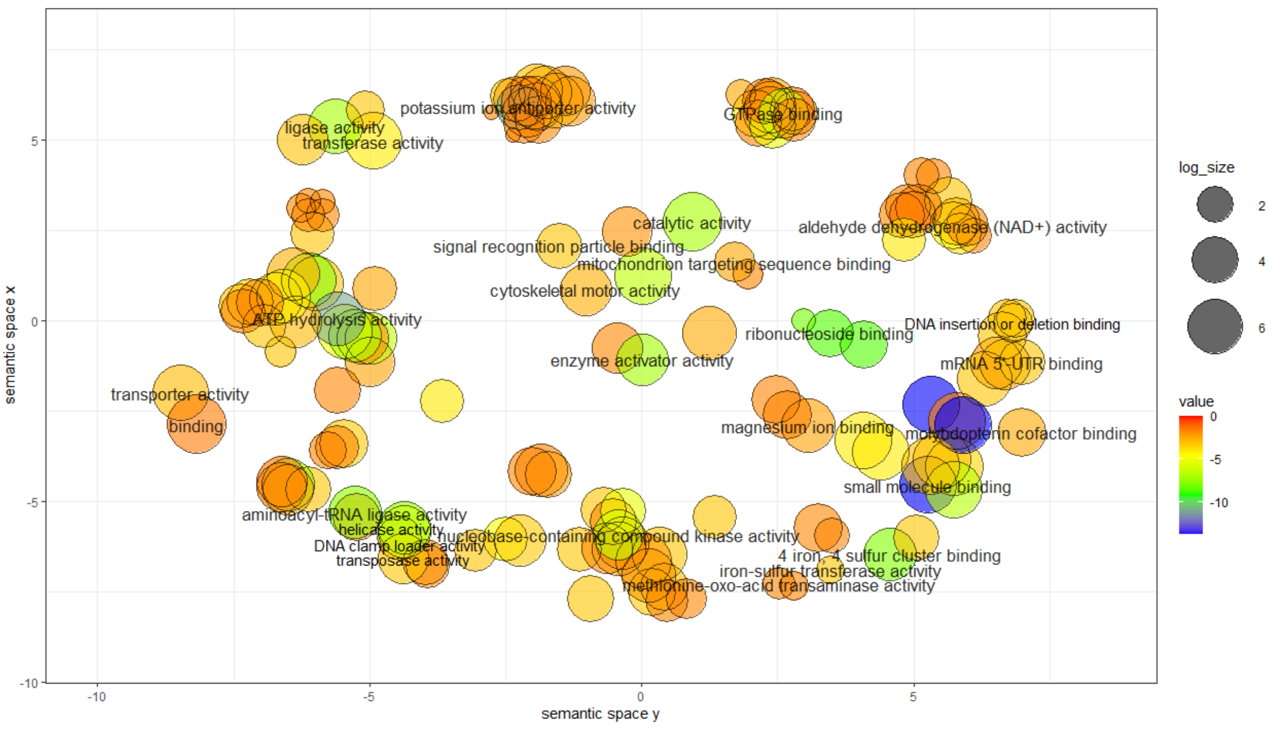
potassium ion antiporter activity
GTPase binding
ligase activity
transferase activity
catalytic activity
aldehyde dehydrogenase (NAD+) activity
signal recognition particle binding
mitochondrion targeting sequence binding
cytoskeletal motor activity
ATP hydrolysis activity
DNA insertion or deletion binding
ribonucleoside binding
enzyme activator activity
mRNA 5'-UTR binding
transporter activity
binding
magnesium ion binding
molybdopterin cofactor binding
small molecule binding
aminoacyl-tRNA ligase activity
helicase activity
nucleobase-containing compound kinase activity
DNA clamp loader activity
transposase activity
4 iron, 4 sulfur cluster binding
iron-sulfur transferase activity
methionine-oxo-acid transaminase activity
semantic space x
semantic space y
log_size
value

G –

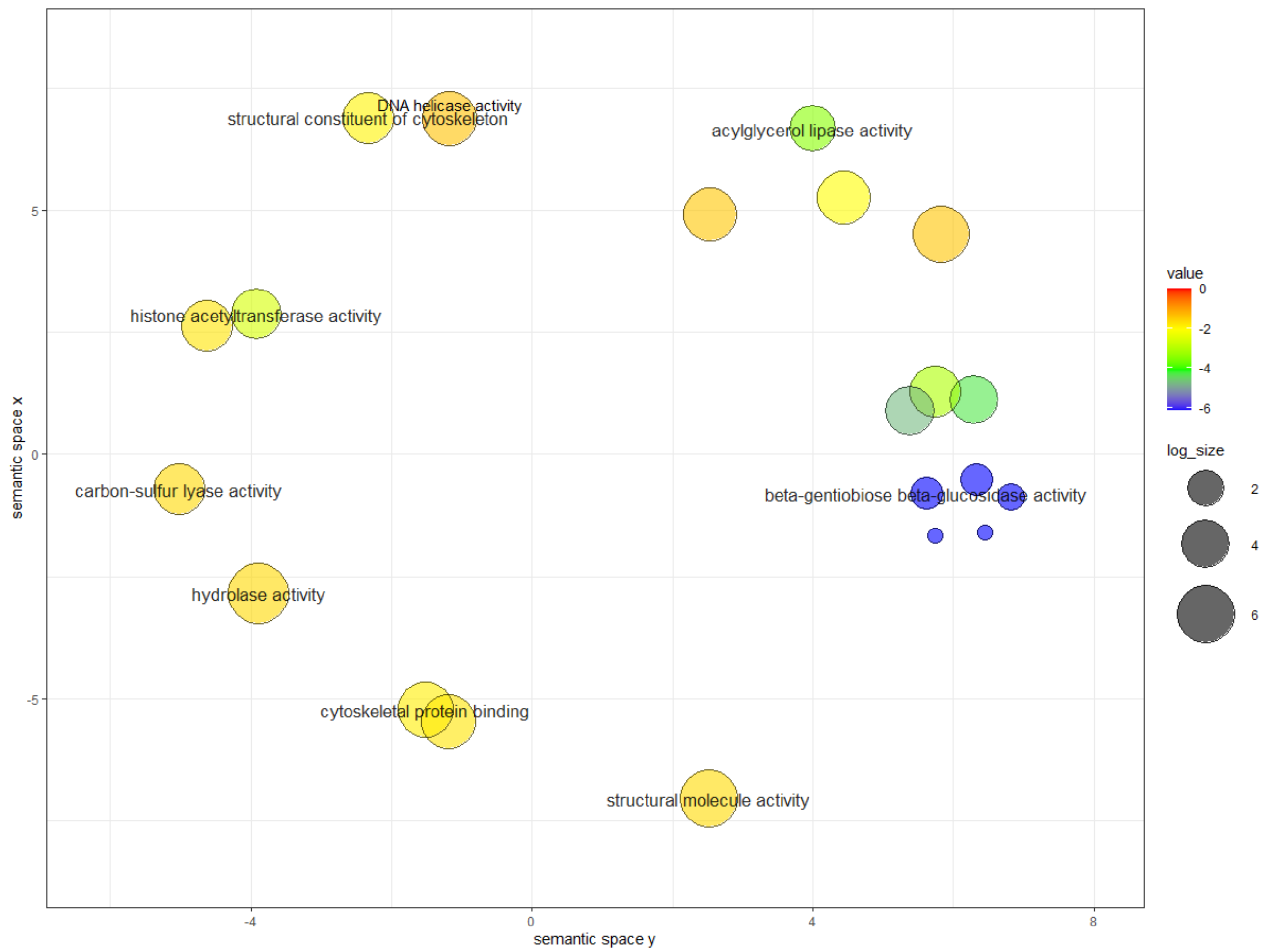
DNA helicase activity
structural constituent of cytoskeleton
acylglycerol lipase activity
histone acetyltransferase activity
carbon-sulfur lyase activity
beta-gentiobiose beta-glucosidase activity
hydrolase activity
cytoskeletal protein binding
structural molecule activity
semantic space x
semantic space y
5
0
-5
-4
0
4
8
value
0
-2
-4
-6
log_size
2
4
6

H –

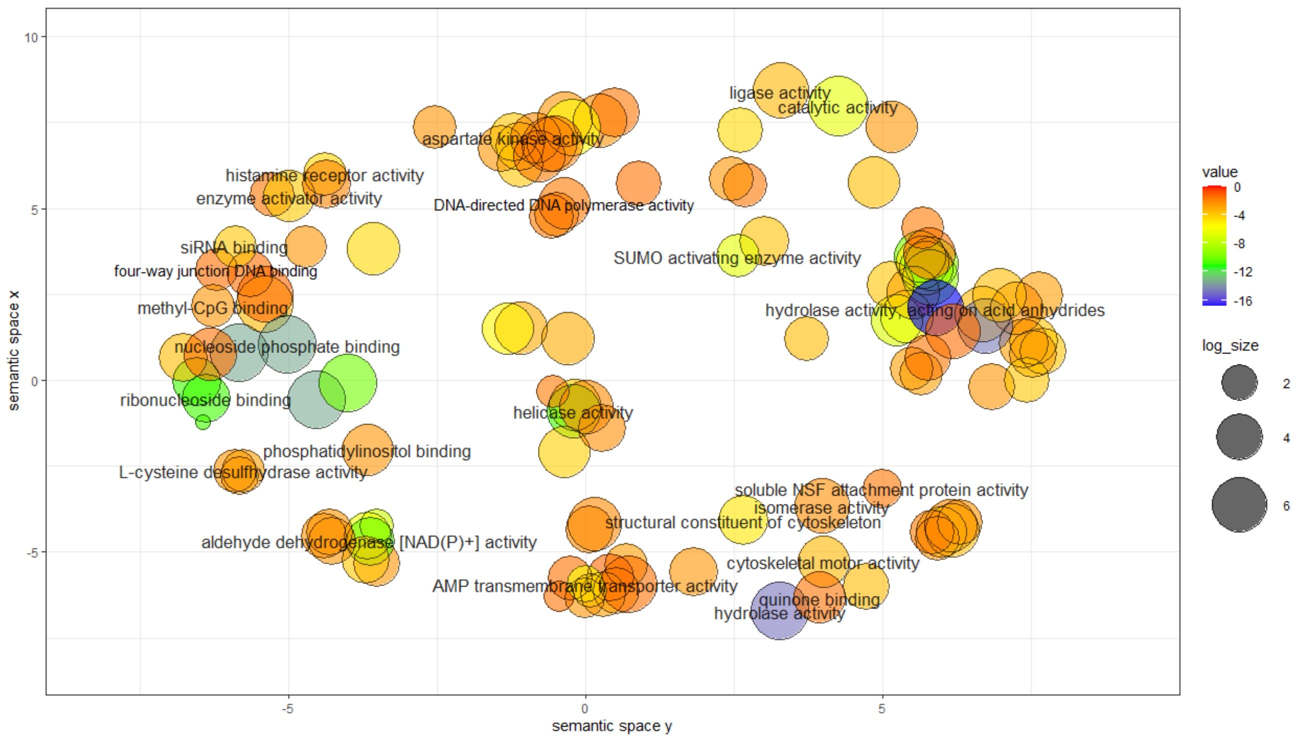
ligase activity
catalytic activity
aspartate kinase activity
histamine receptor activity
enzyme activator activity
DNA-directed DNA polymerase activity
siRNA binding
four-way junction DNA binding
SUMO activating enzyme activity
methyl-CpG binding
hydrolase activity, acting on acid anhydrides
nucleoside phosphate binding
ribonucleoside binding
helicase activity
phosphatidylinositol binding
L-cysteine desulfhydrase activity
soluble NSF attachment protein activity
isomerase activity
structural constituent of cytoskeleton
aldehyde dehydrogenase [NAD(P)+] activity
cytoskeletal motor activity
AMP transmembrane transporter activity
quinone binding
hydrolase activity
semantic space x
semantic space y
10
5
0
-5
-5
0
5
value
0
-4
-8
-12
-16
log_size
2
4
6

I –

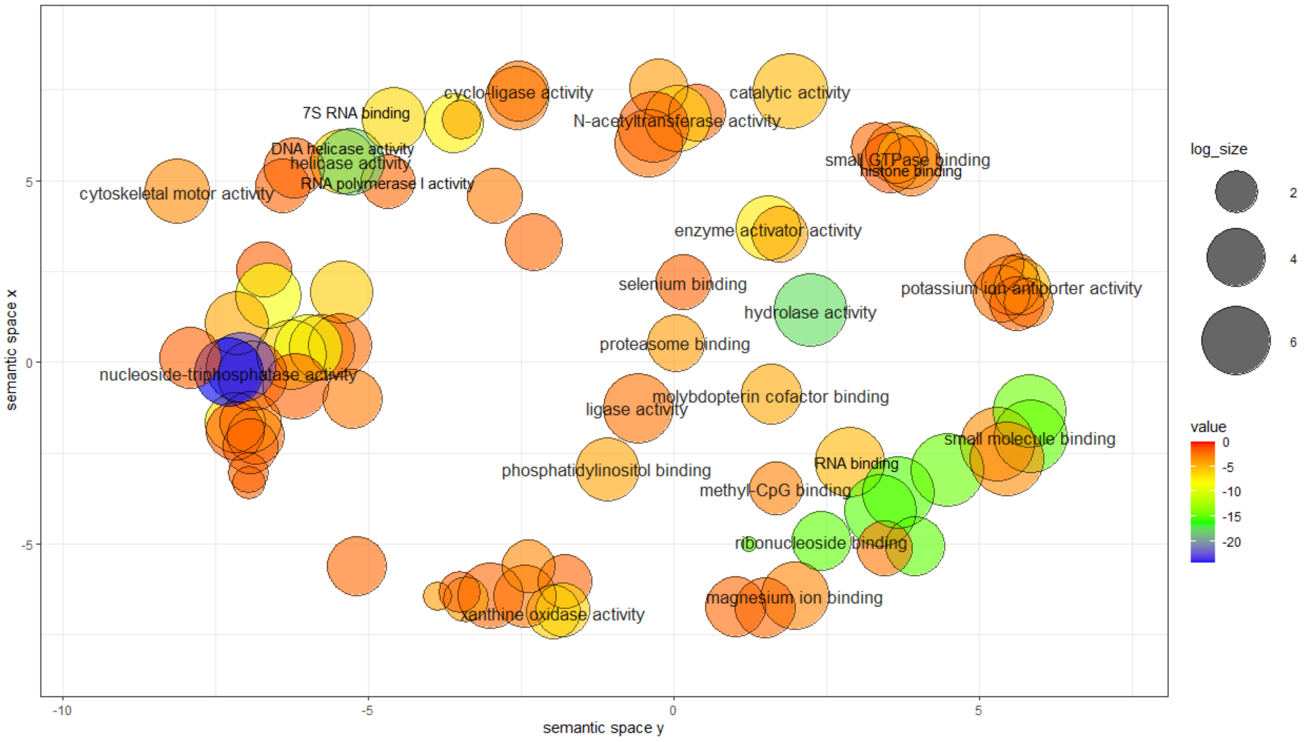
catalytic activity
cyclo-ligase activity
7S RNA binding
N-acetyltransferase activity
DNA helicase activity
helicase activity
small GTPase binding
histone binding
RNA polymerase I activity
cytoskeletal motor activity
enzyme activator activity
selenium binding
potassium ion antiporter activity
hydrolase activity
proteasome binding
nucleoside-triphosphatase activity
molybdopterin cofactor binding
ligase activity
small molecule binding
RNA binding
phosphatidylinositol binding
methyl-CpG binding
ribonucleoside binding
magnesium ion binding
xanthine oxidase activity
semantic space x
semantic space y
log_size
value

J –

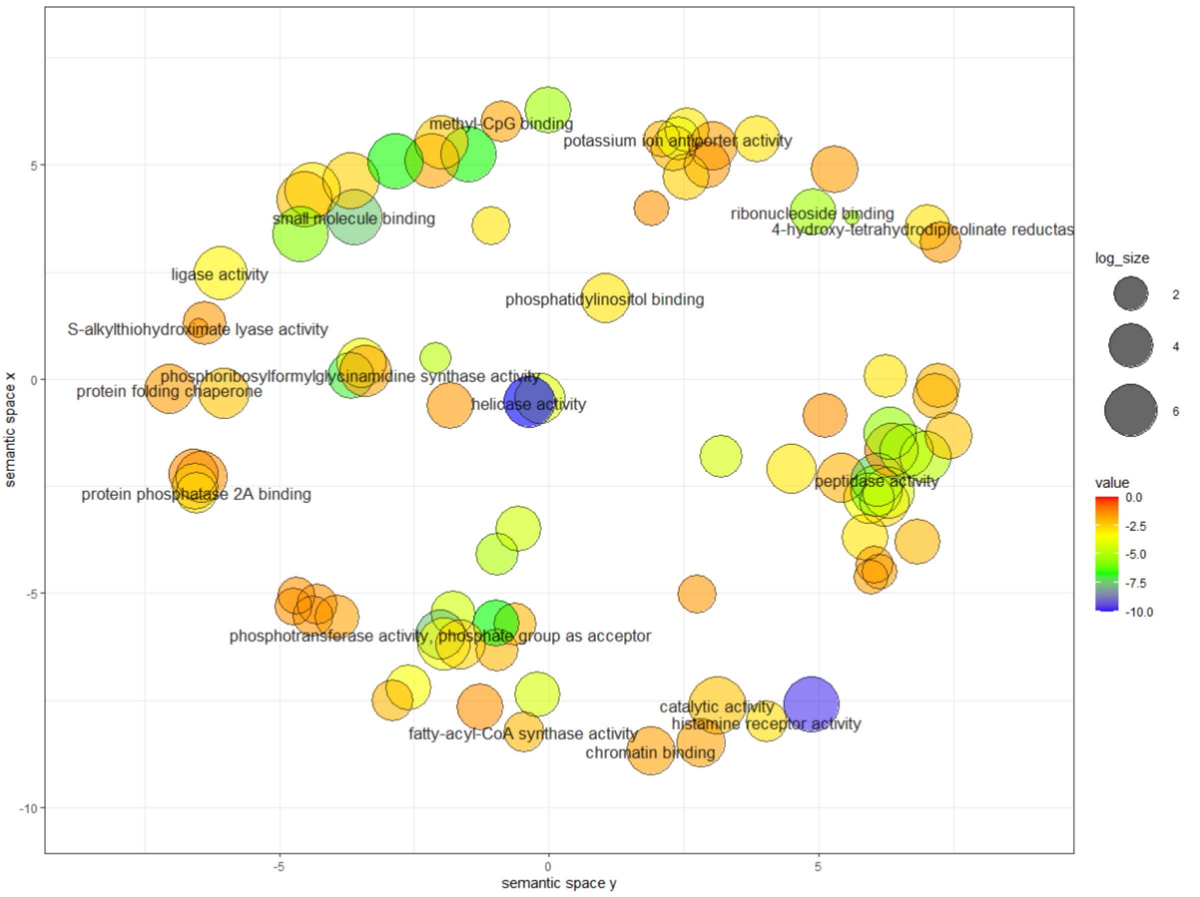
methyl-CpG binding
potassium ion antiporter activity
ribonucleoside binding
small molecule binding
4-hydroxy-tetrahydrodipicolinate reductas
ligase activity
phosphatidylinositol binding
S-alkylthiohydroximate lyase activity
phosphoribosylformylglycinamidine synthase activity
protein folding chaperone
helicase activity
protein phosphatase 2A binding
peptidase activity
phosphotransferase activity, phosphate group as acceptor
catalytic activity
histamine receptor activity
fatty-acyl-CoA synthase activity
chromatin binding
semantic space x
semantic space y
log_size
value

K –

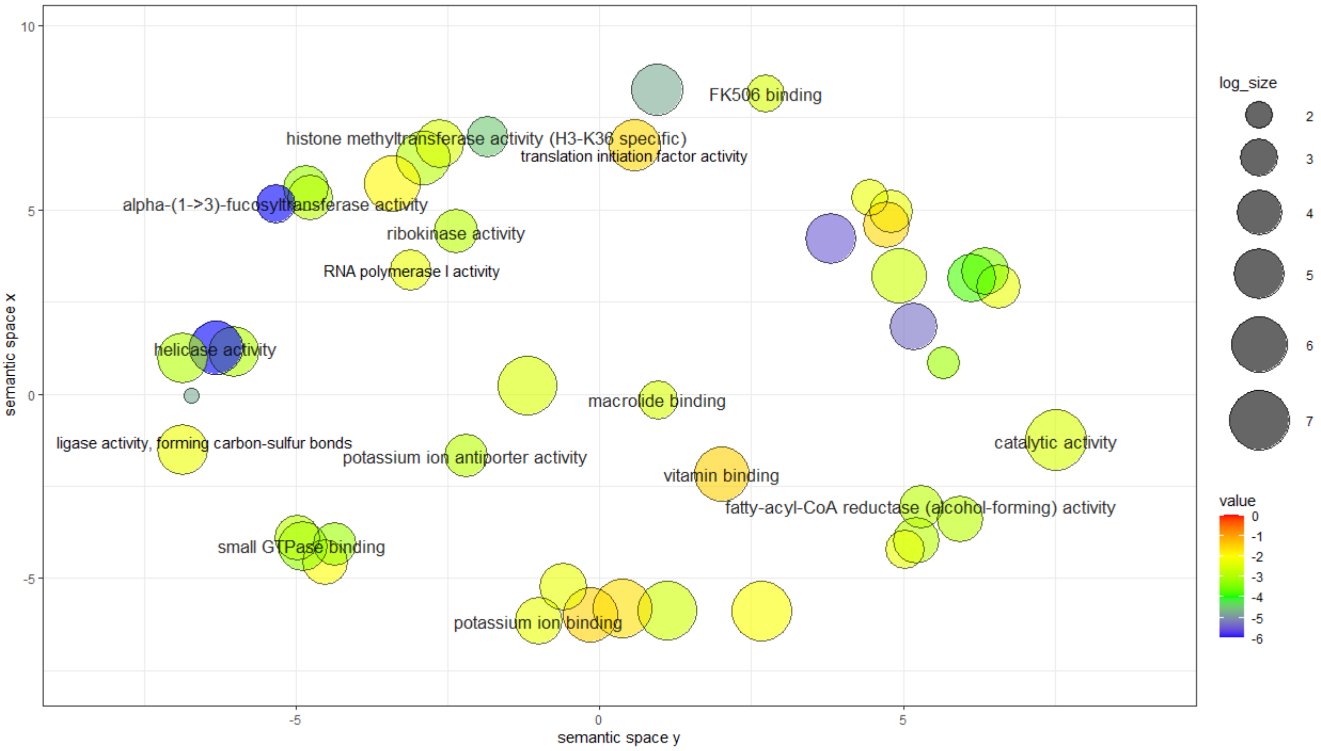
FK506 binding
histone methyltransferase activity (H3-K36 specific)
translation initiation factor activity
alpha-(1->3)-fucosyltransferase activity
ribokinase activity
RNA polymerase I activity
helicase activity
macrolide binding
ligase activity, forming carbon-sulfur bonds
potassium ion antiporter activity
catalytic activity
vitamin binding
fatty-acyl-CoA reductase (alcohol-forming) activity
small GTPase binding
potassium ion binding
semantic space x
semantic space y
log_size
value

L –

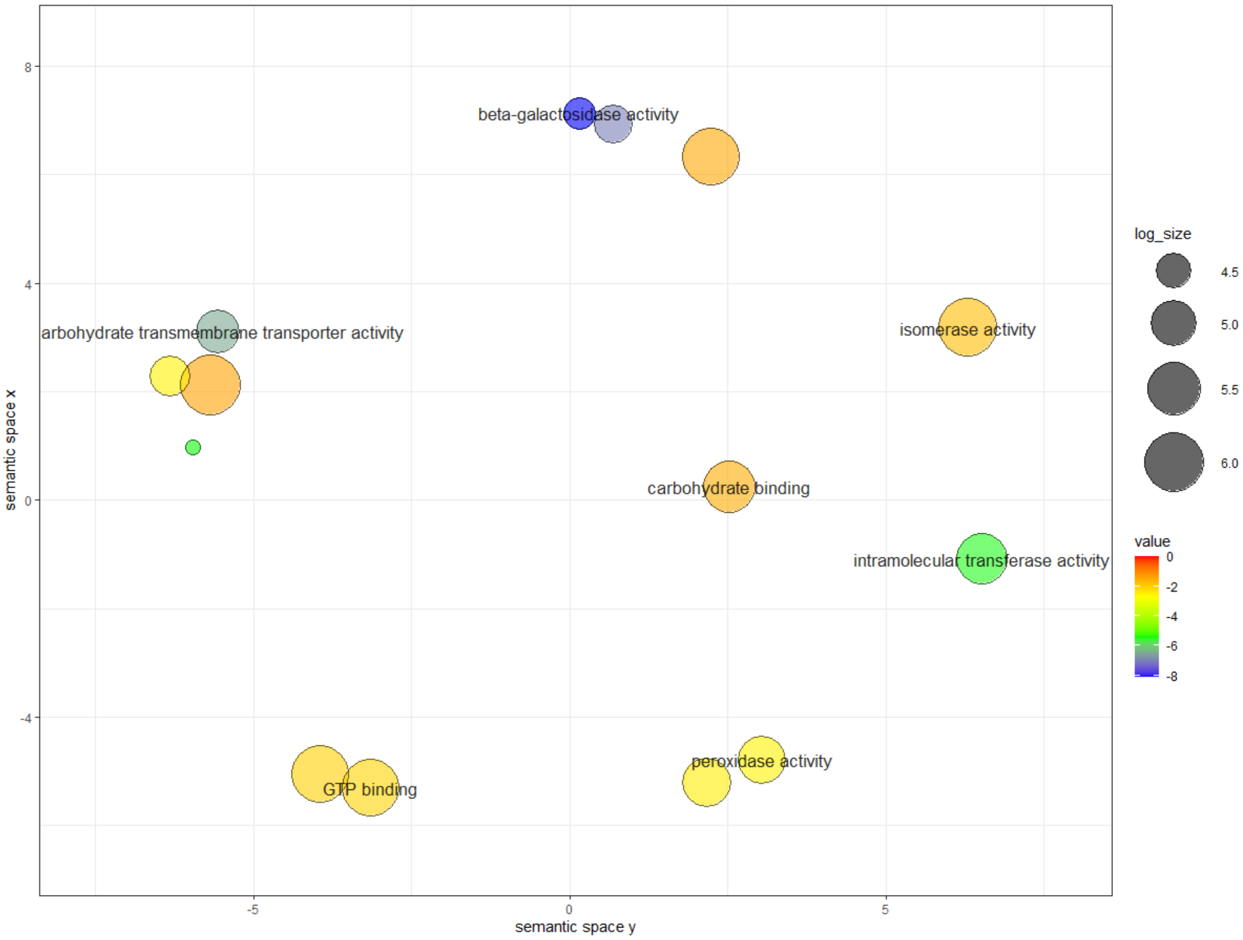
beta-galactosidase activity
arbohydrate transmembrane transporter activity
isomerase activity
carbohydrate binding
intramolecular transferase activity
GTP binding
peroxidase activity
semantic space x
semantic space y
log_size
value

M –

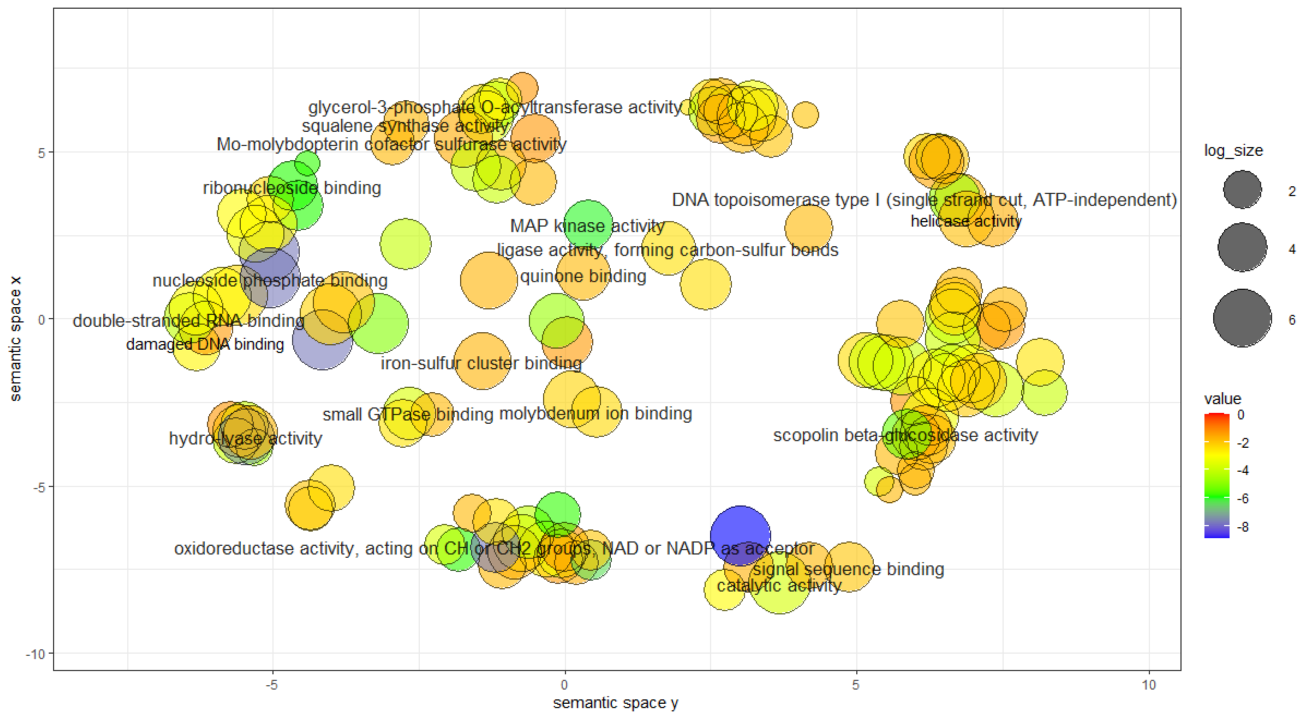

Figure S3 – Enriched terms for MF category for all triads with all superfamilies A-DTA, B-DTC, C-DTH, D-DTM, E-DTT, F-DTX, G-DXX, H-RIX, I-RLC, J-RLG, K-RLX, L-SIX, M-XXX. The scatterplots were generated by REVIGO , bubble color indicates the FDR value and the size indicates the frequency of the GO term in the GOA database, bubbles of more general terms are larger.

A –

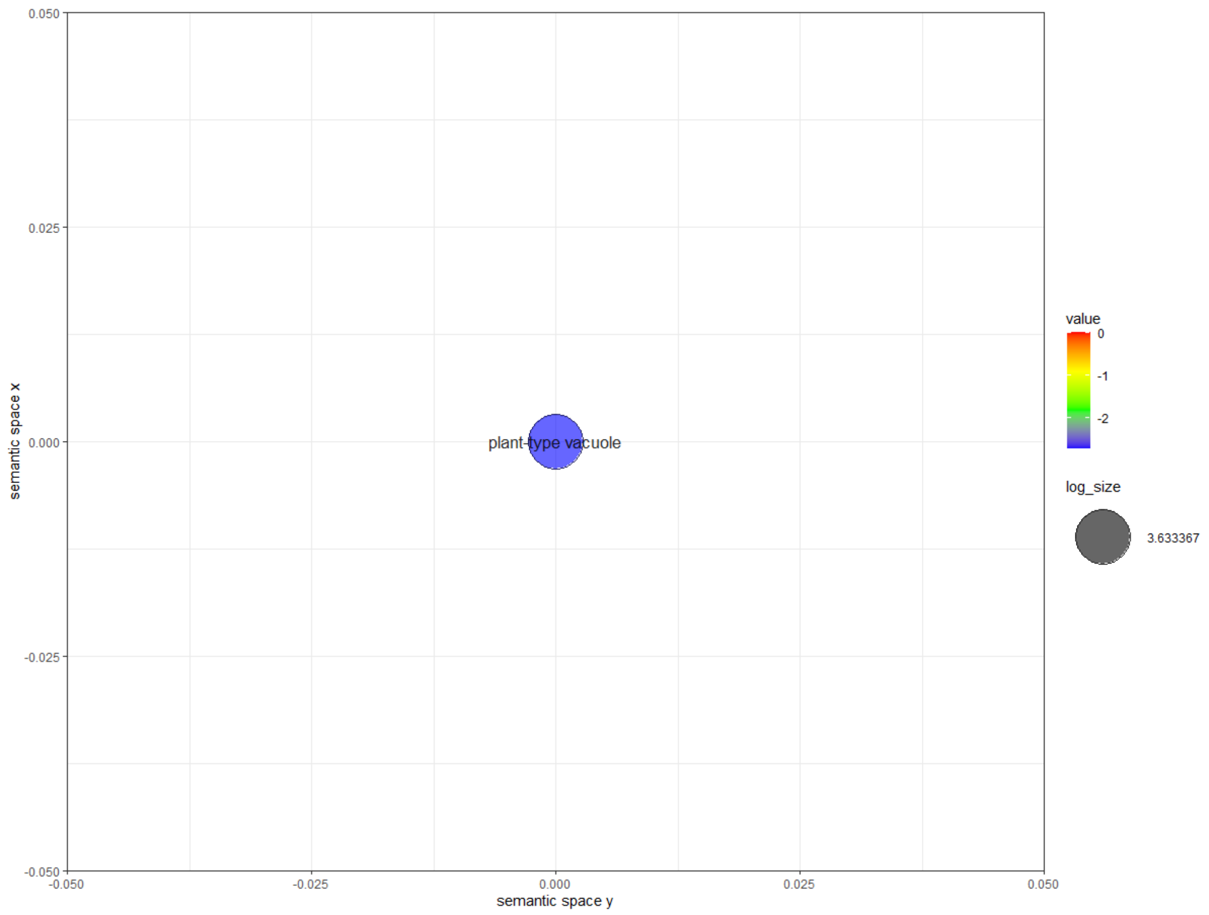

B –

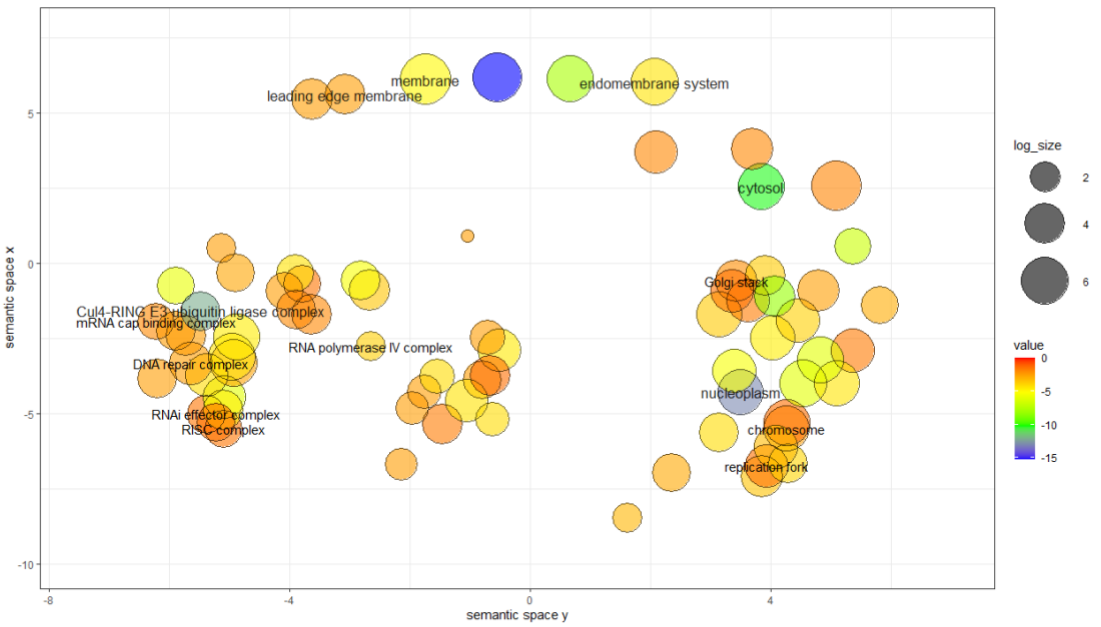
membrane
leading edge membrane
endomembrane system
cytosol
Golgi stack
Cul4-RING E3 ubiquitin ligase complex
mRNA cap binding complex
DNA repair complex
RNA polymerase IV complex
nucleoplasm
RNAi effector complex
RISC complex
chromosome
replication fork
semantic space x
semantic space y
log_size
value

C –

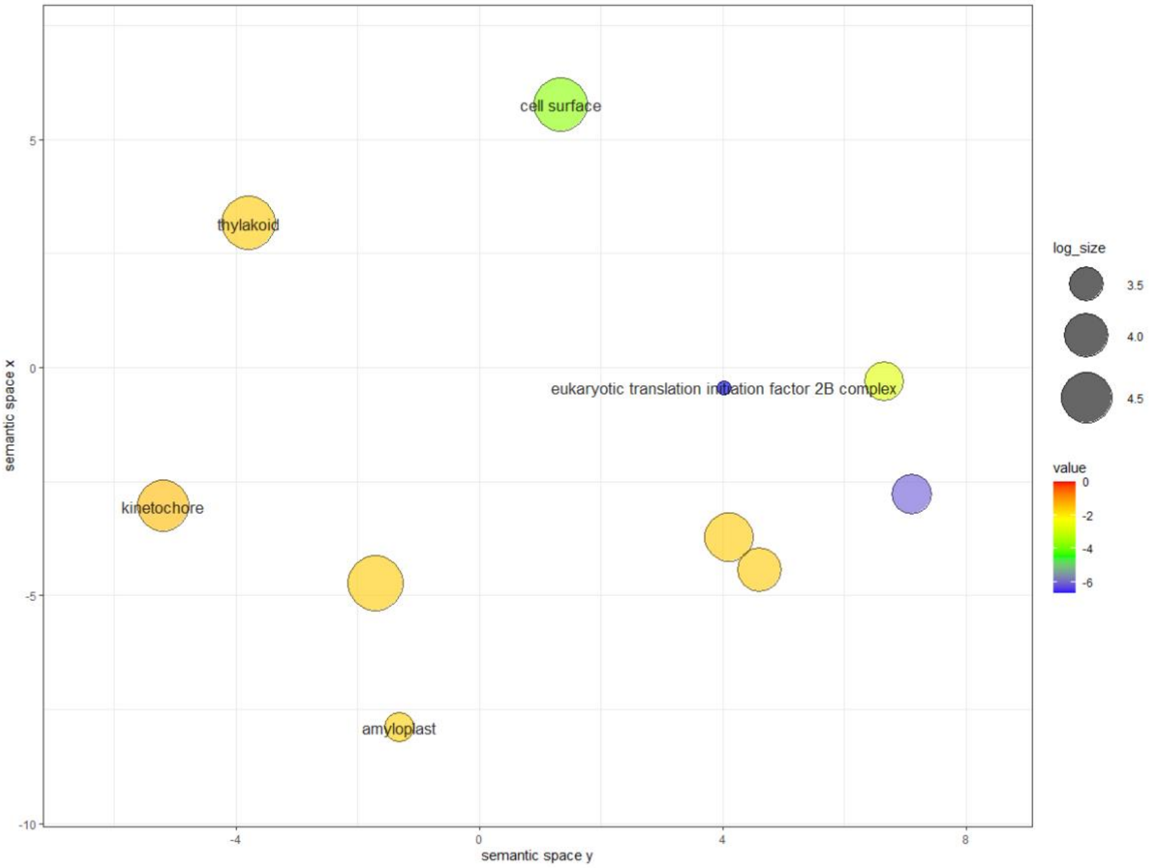
cell surface
thylakoid
eukaryotic translation initiation factor 2B complex
kinetochore
amyloplast
semantic space x
semantic space y
log_size
value

D –

E –

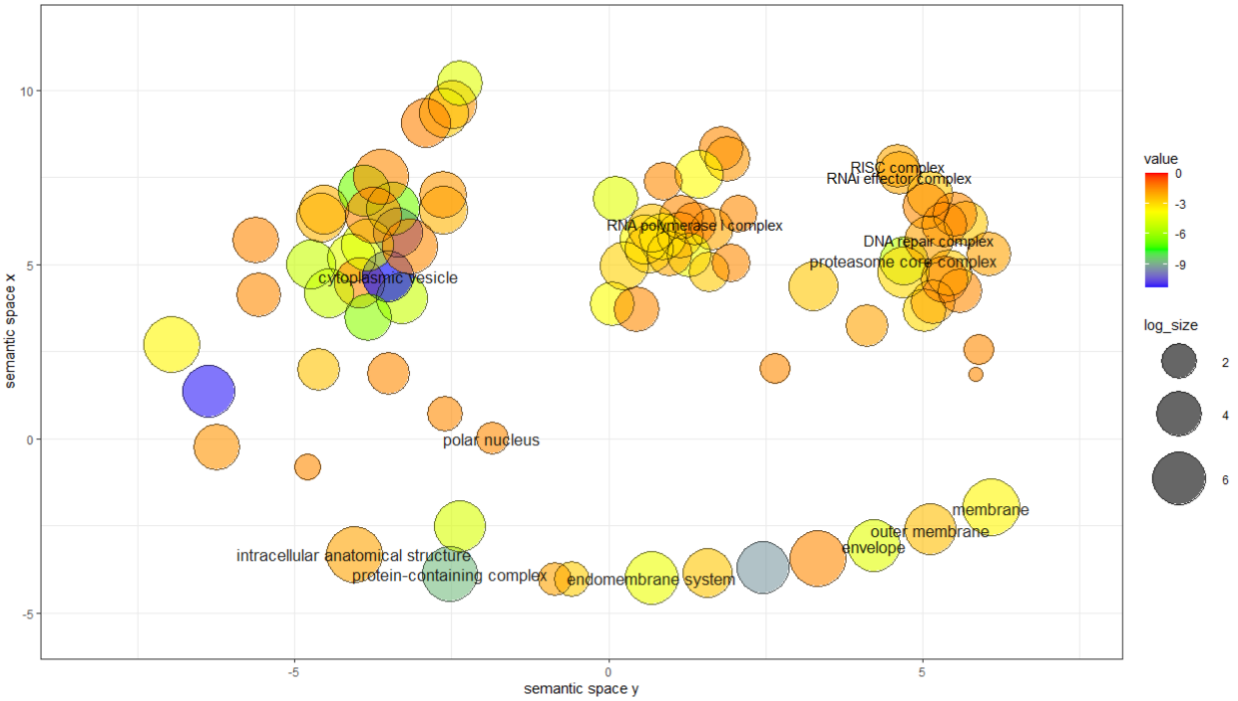

RISC complex
RNAi effector complex
RNA polymerase I complex
DNA repair complex
proteasome core complex
cytoplasmic vesicle
polar nucleus
membrane
outer membrane
envelope
intracellular anatomical structure
protein-containing complex
endomembrane system
semantic space x
semantic space y
value
log_size

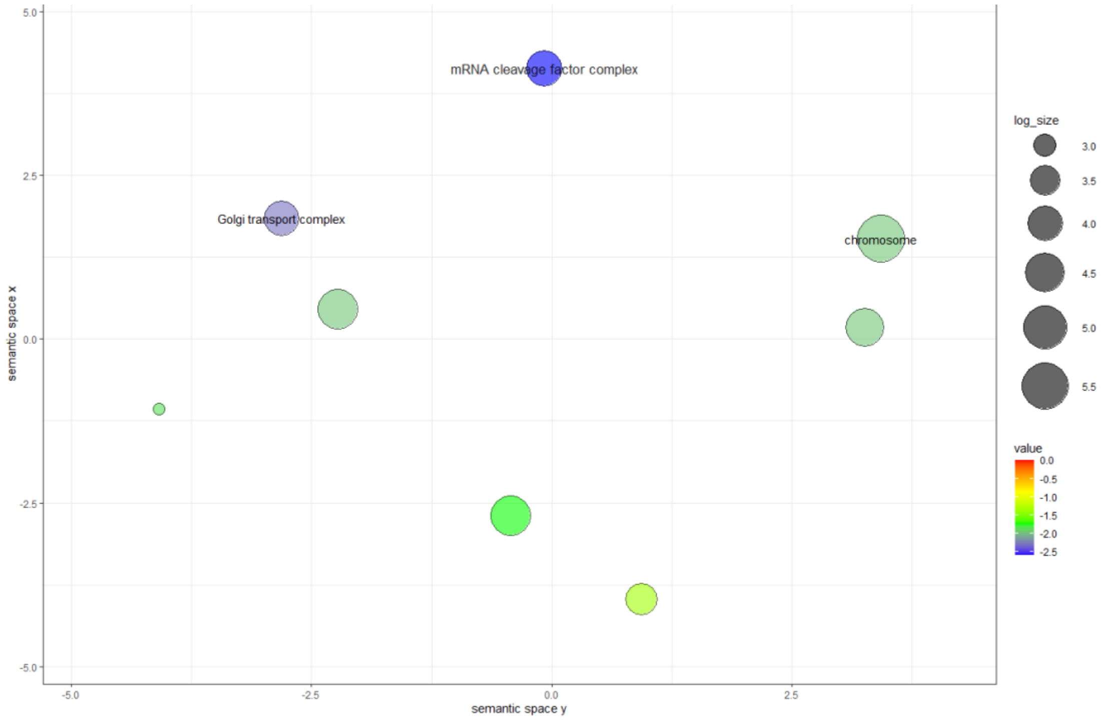

mRNA cleavage factor complex
Golgi transport complex
chromosome
semantic space x
semantic space y
log_size
value

F –

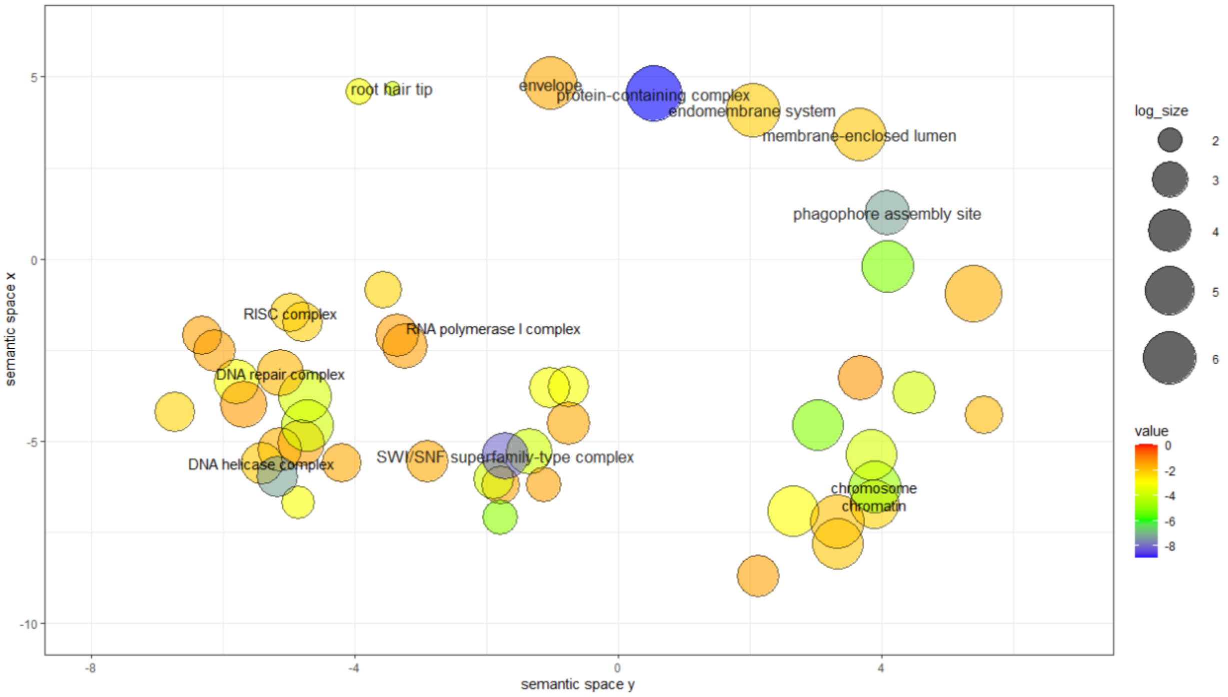
root hair tip
envelope
protein-containing complex
endomembrane system
membrane-enclosed lumen
phagophore assembly site
RISC complex
RNA polymerase I complex
DNA repair complex
SWI/SNF superfamily-type complex
DNA helicase complex
chromosome
chromatin
semantic space x
semantic space y
log_size
value

G –

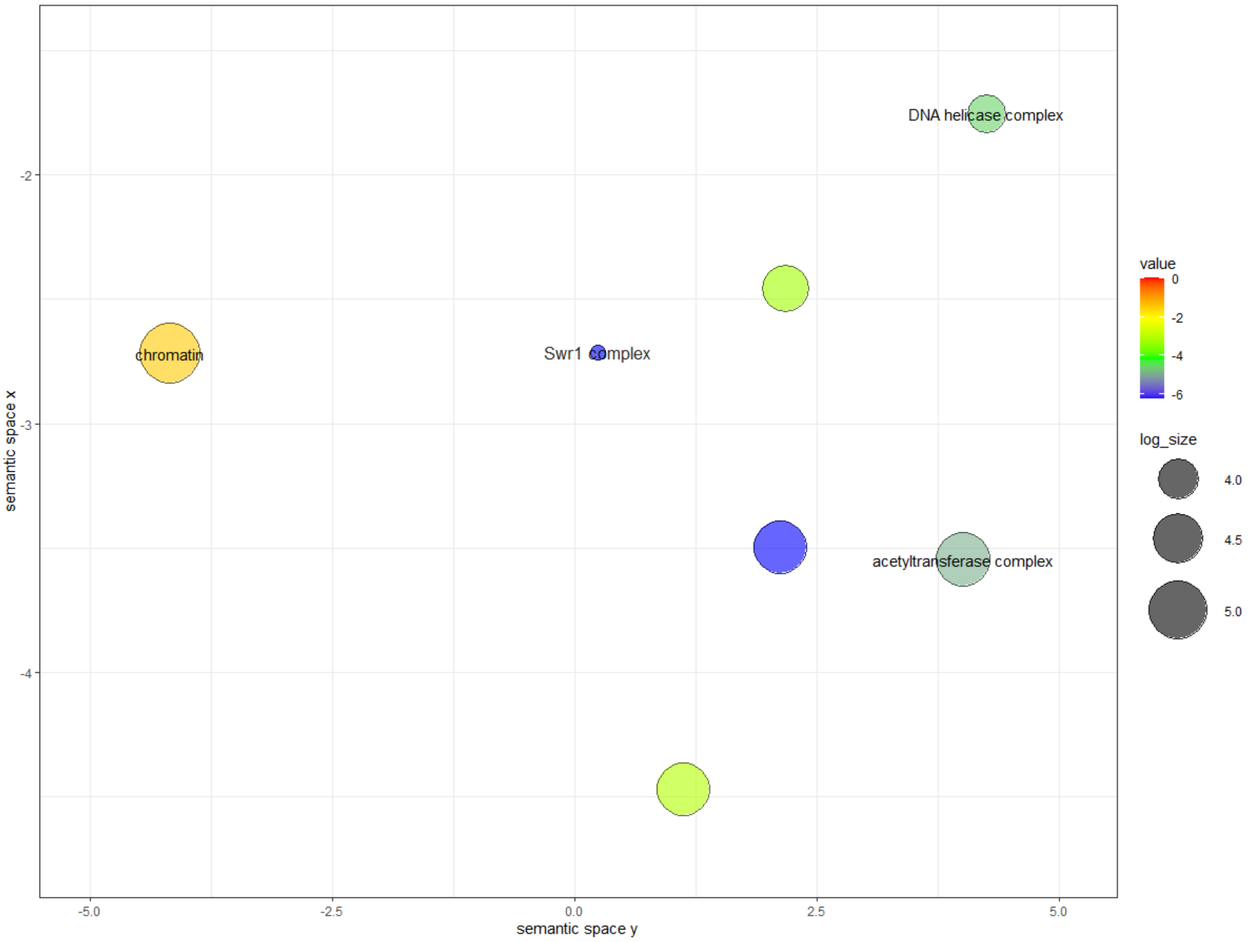
DNA helicase complex
chromatin
Swr1 complex
acetyltransferase complex
semantic space x
semantic space y
value
log_size

H –

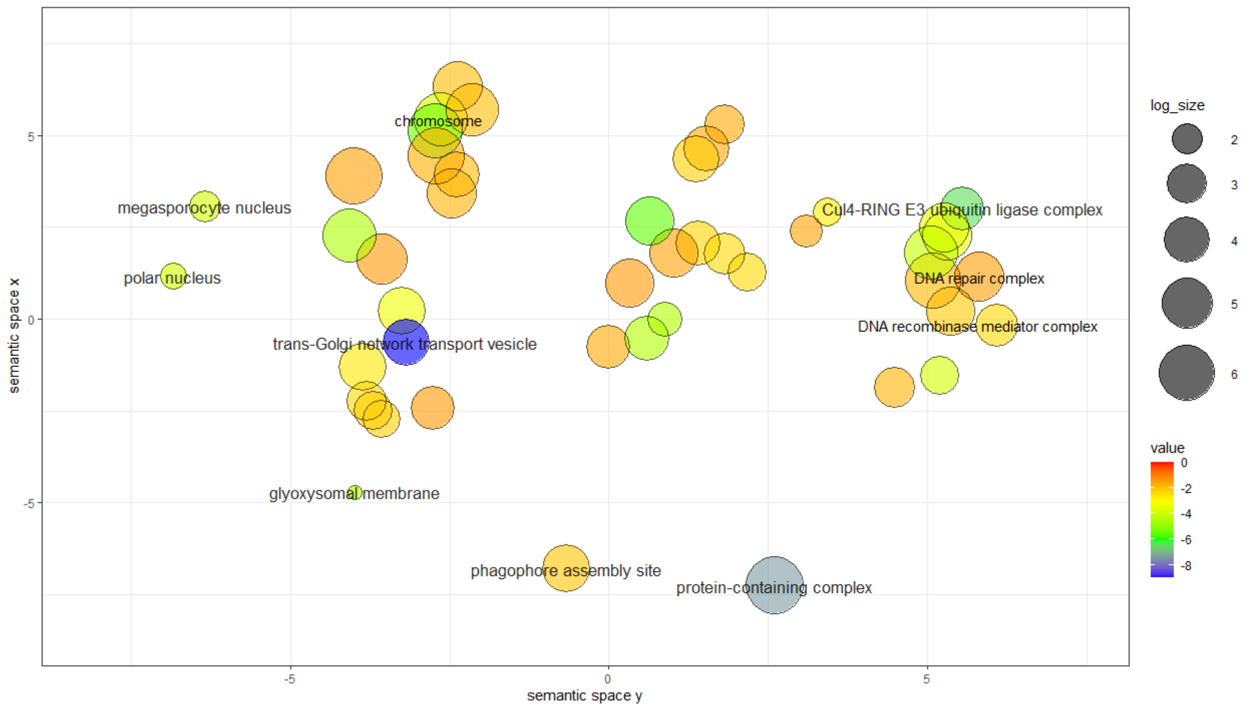
chromosome
megasporocyte nucleus
polar nucleus
trans-Golgi network transport vesicle
glyoxysomal membrane
phagophore assembly site
protein-containing complex
Cul4-RING E3 ubiquitin ligase complex
DNA repair complex
DNA recombinase mediator complex
semantic space x
semantic space y
log_size
value

I –

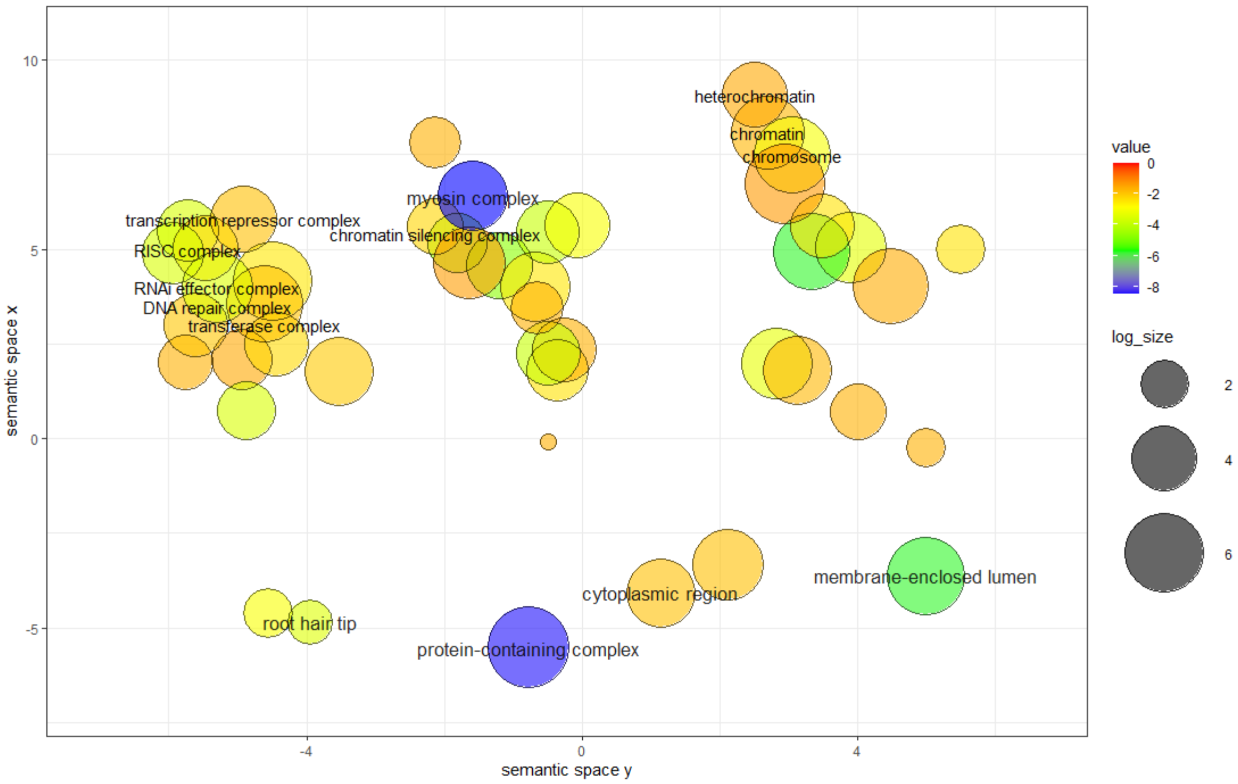
heterochromatin
chromatin
chromosome
myosin complex
transcription repressor complex
chromatin silencing complex
RISC complex
RNAi effector complex
DNA repair complex
transferase complex
cytoplasmic region
membrane-enclosed lumen
root hair tip
protein-containing complex
semantic space x
semantic space y
value
log_size

J –

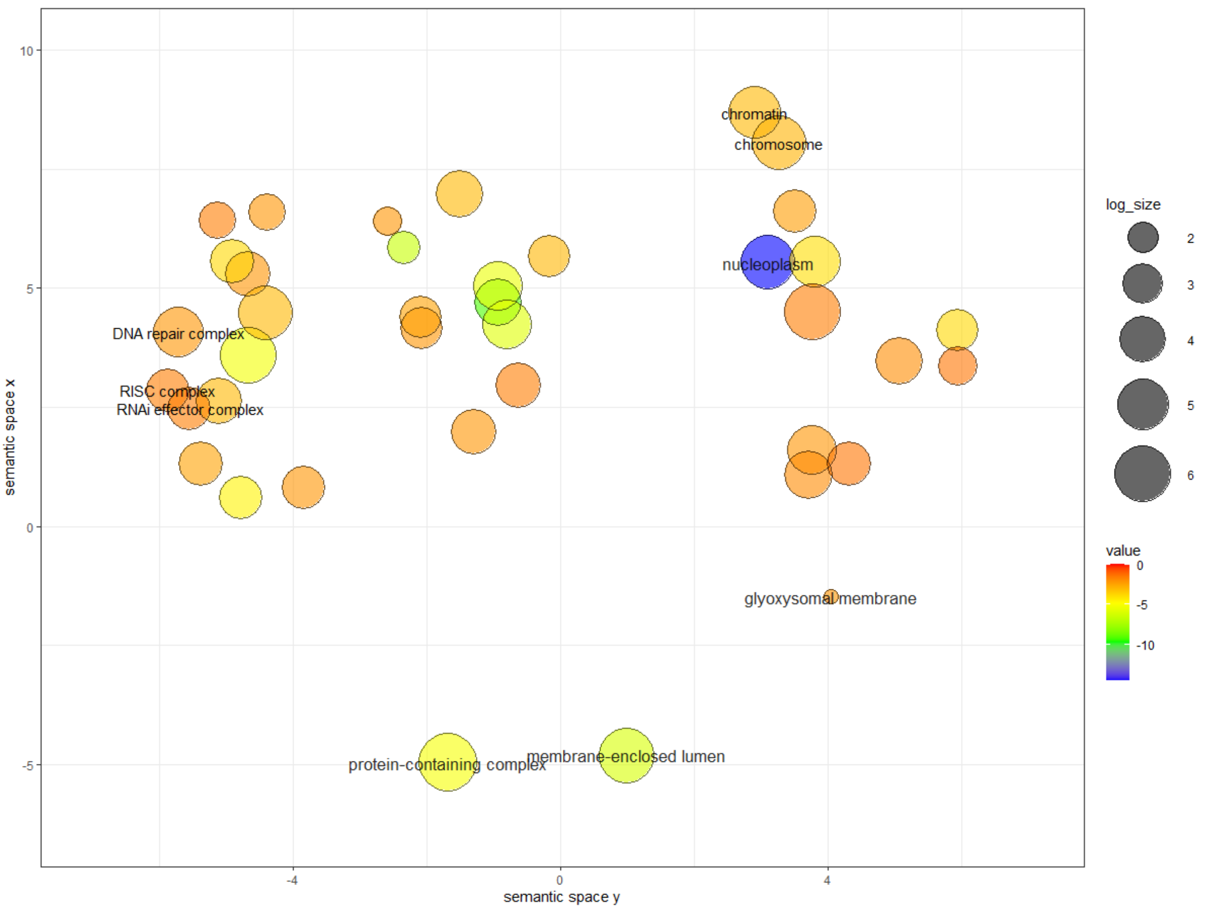
chromatin
chromosome
nucleoplasm
DNA repair complex
RISC complex
RNAi effector complex
glyoxysomal membrane
protein-containing complex
membrane-enclosed lumen
semantic space x
semantic space y
log_size
value

K –

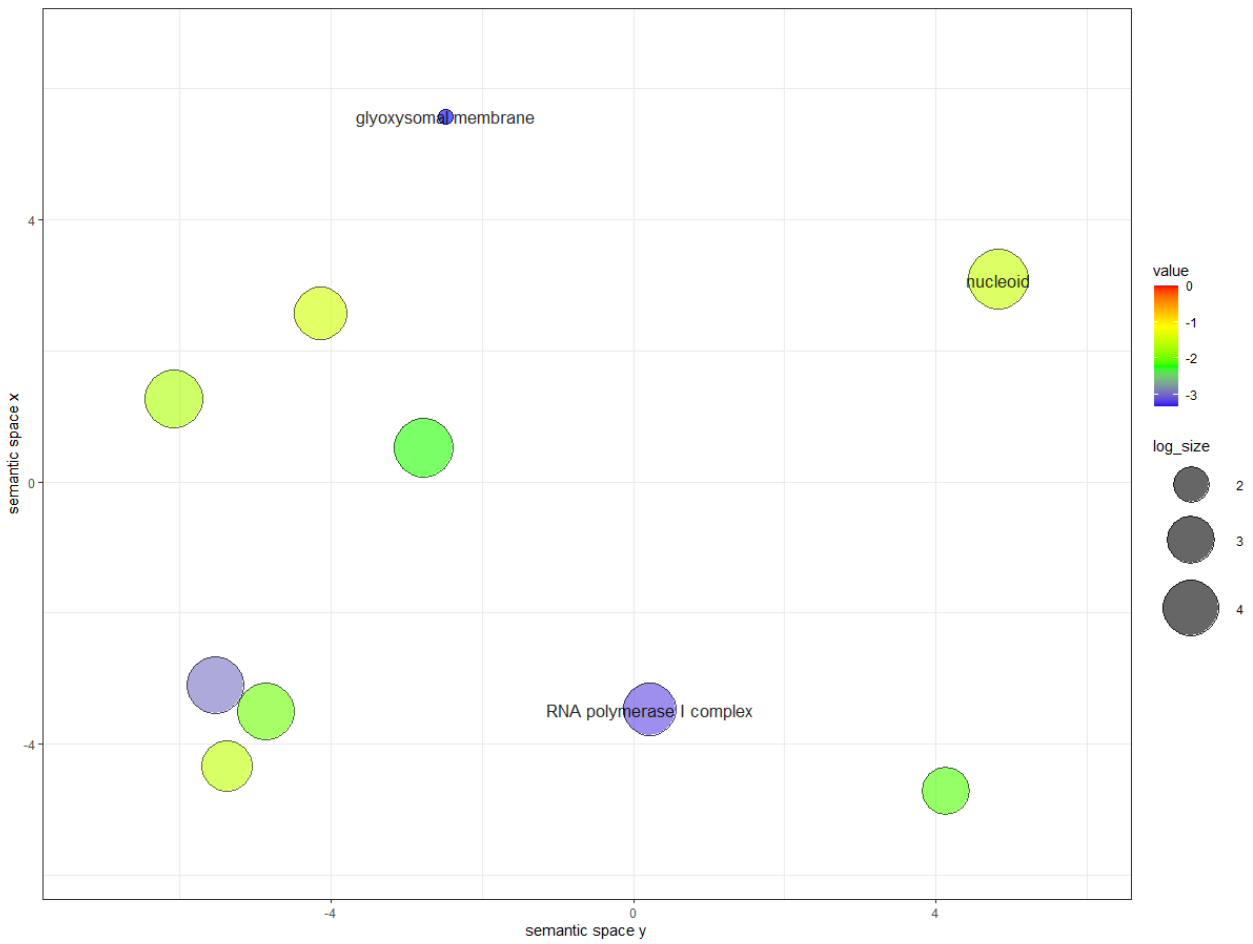
glyoxysomal membrane
nucleoid
RNA polymerase I complex
semantic space x
semantic space y
value
log_size

L –

M –

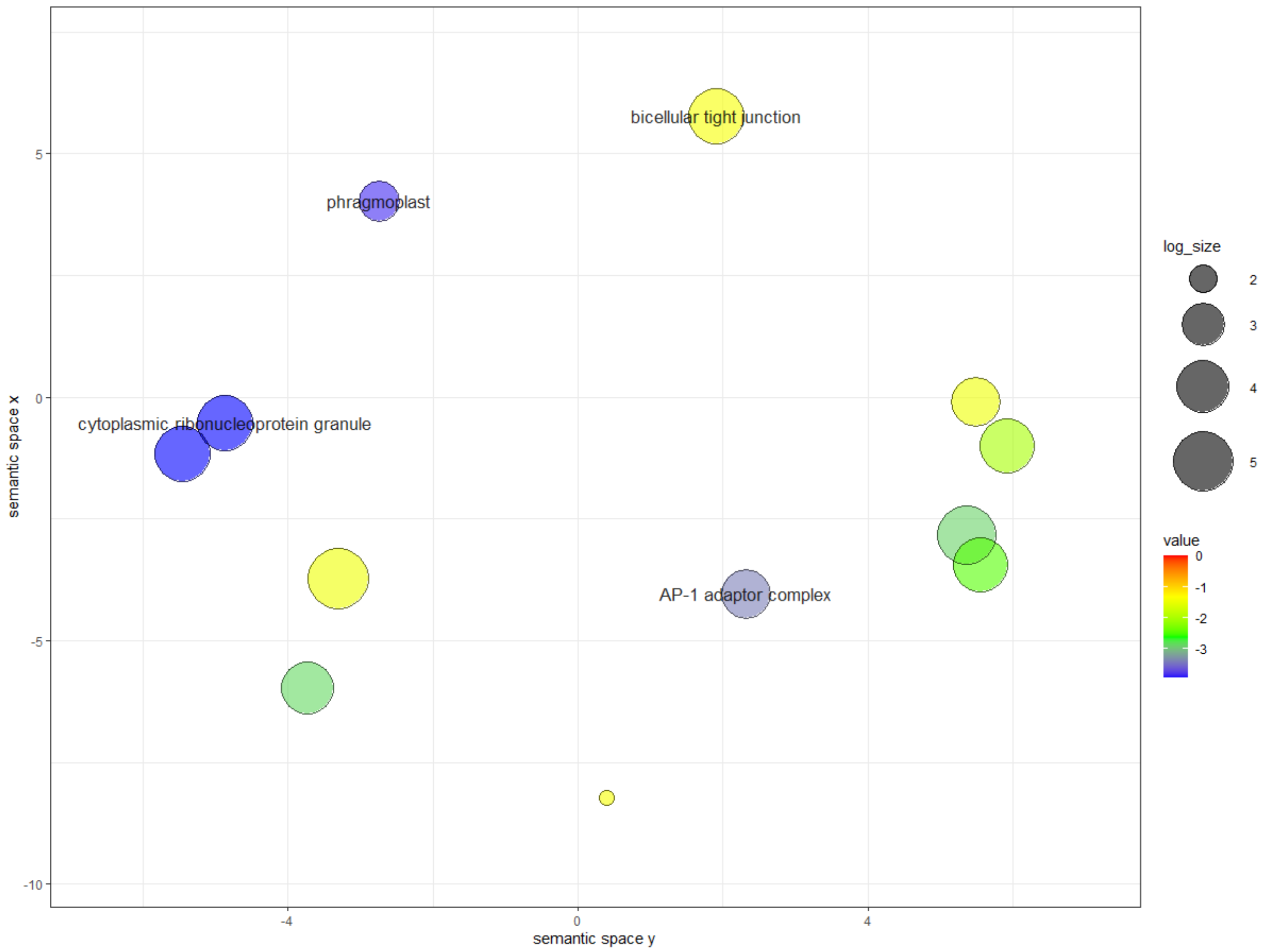

bicellular tight junction
phragmoplast
cytoplasmic ribonucleoprotein granule
AP-1 adaptor complex
semantic space x
semantic space y
5
0
-5
-10
-4
0
4
log_size
2
3
4
5
value
0
-1
-2
-3

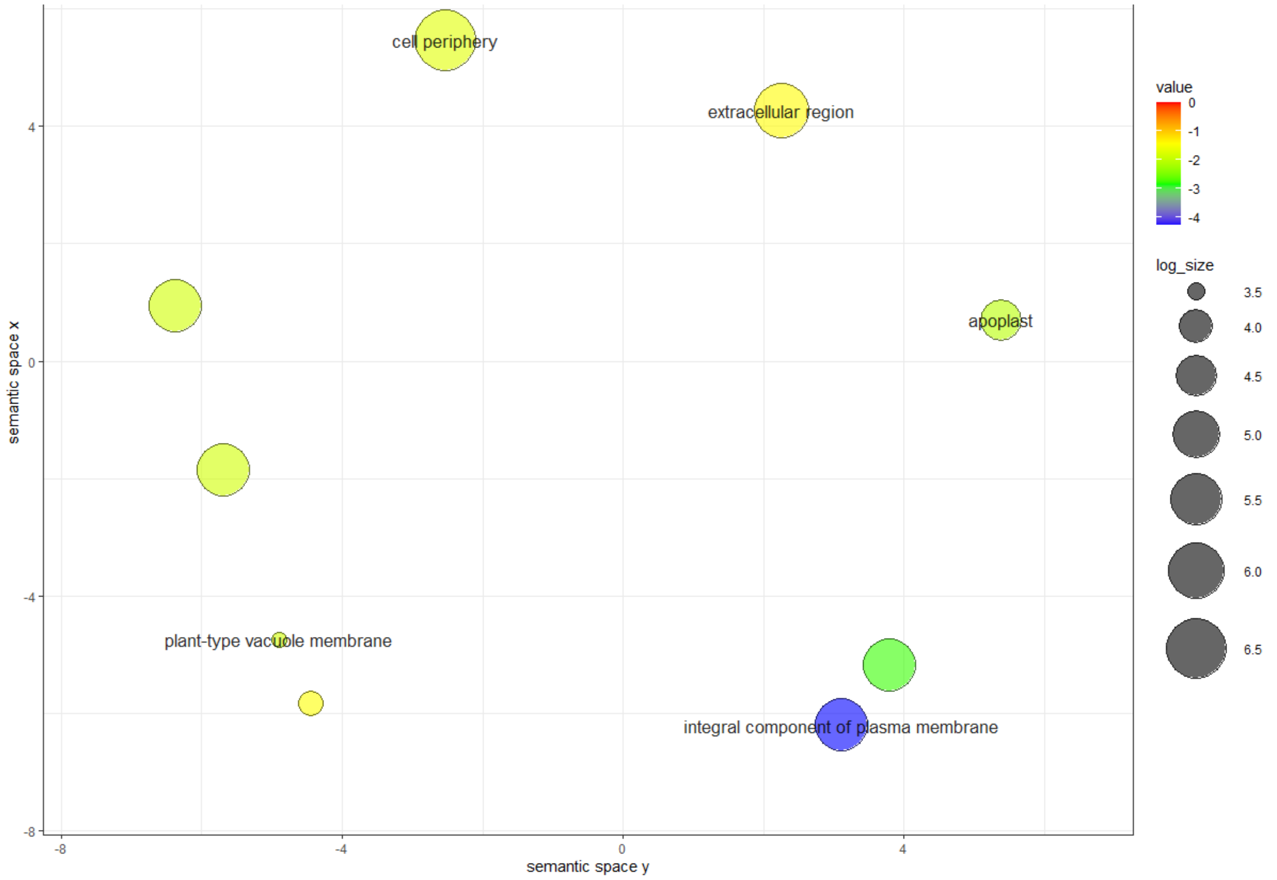

cell periphery
extracellular region
apoplast
plant-type vacuole membrane
integral component of plasma membrane
semantic space x
semantic space y
4
0
-4
-8
-8
-4
0
4
value
0
-1
-2
-3
-4
log_size
3.5
4.0
4.5
5.0
5.5
6.0
6.5

Figure S4 – Enriched terms for CC category for all triads with all superfamilies A-DTA, B-DTC, C-DTH, D-DTM, E-DTT, F-DTX, G-DXX, H-RIX, I-RLC, J-RLG, K-RLX, L-SIX, M-XXX. The scatterplots were generated by REVIGO , bubble color indicates the FDR value and the size indicates the frequency of the GO term in the GOA database, bubbles of more general terms are larger.

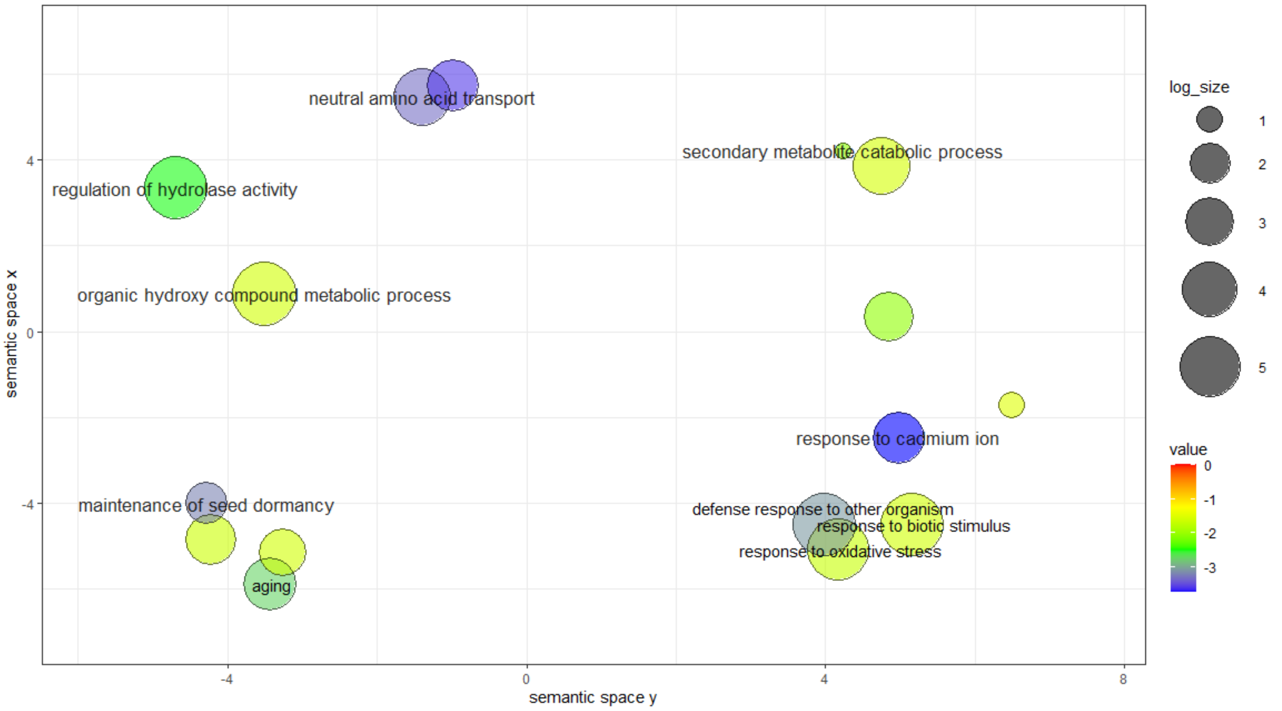

Figure S5 – Enriched terms for BP category for triads that include TE insertions from DTM superfamily and were assigned to the dominant relative expression category. The scatterplot was generated by REVIGO , bubble color indicates the FDR value and the size indicates the frequency of the GO term in the GOA database, bubbles of more general terms are larger.

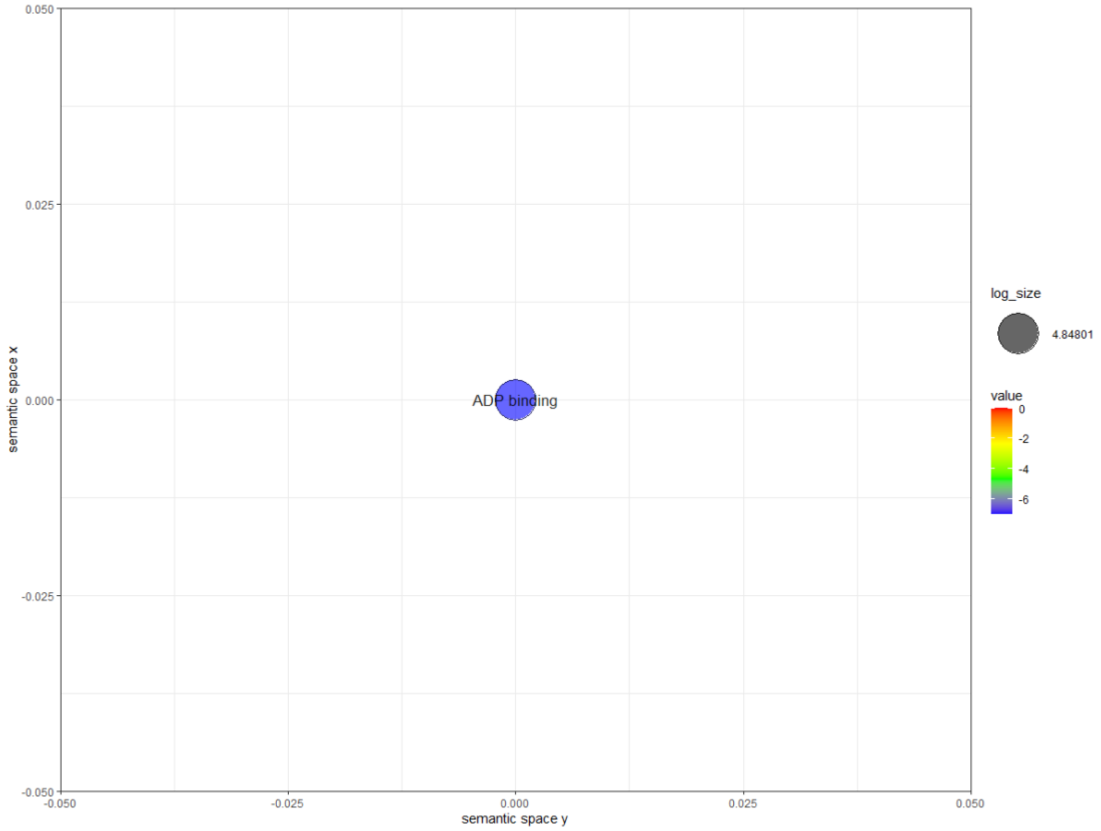

Figure S6 – Enriched terms for MF category for triads that include TE insertions from DTM superfamily and were assigned to the dominant relative expression category. The scatterplot was generated by REVIGO , bubble color indicates the FDR value and the size indicates the frequency of the GO term in the GOA database, bubbles of more general terms are larger.

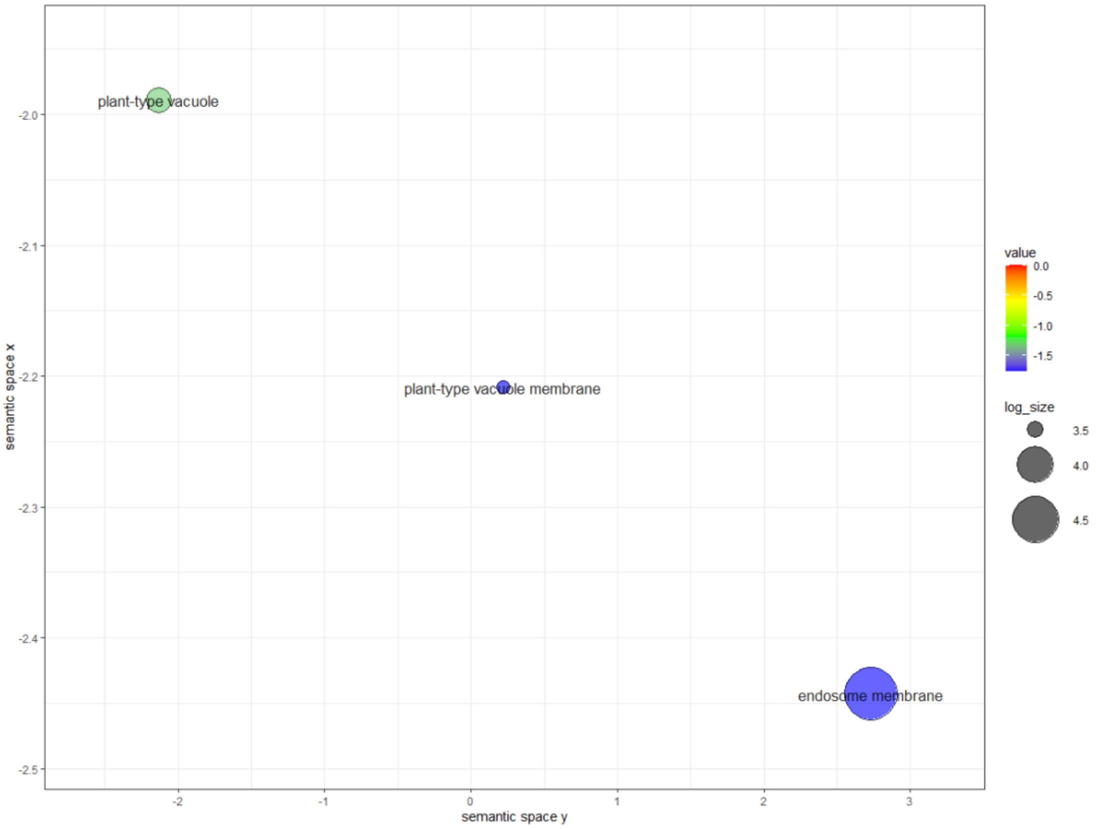

Figure S7 – Enriched terms for CC category for triads that include TE insertions from DTM superfamily and were assigned to the dominant relative expression category. The scatterplot was generated by REVIGO , bubble color indicates the FDR value and the size indicates the frequency of the GO term in the GOA database, bubbles of more general terms are larger.

A –

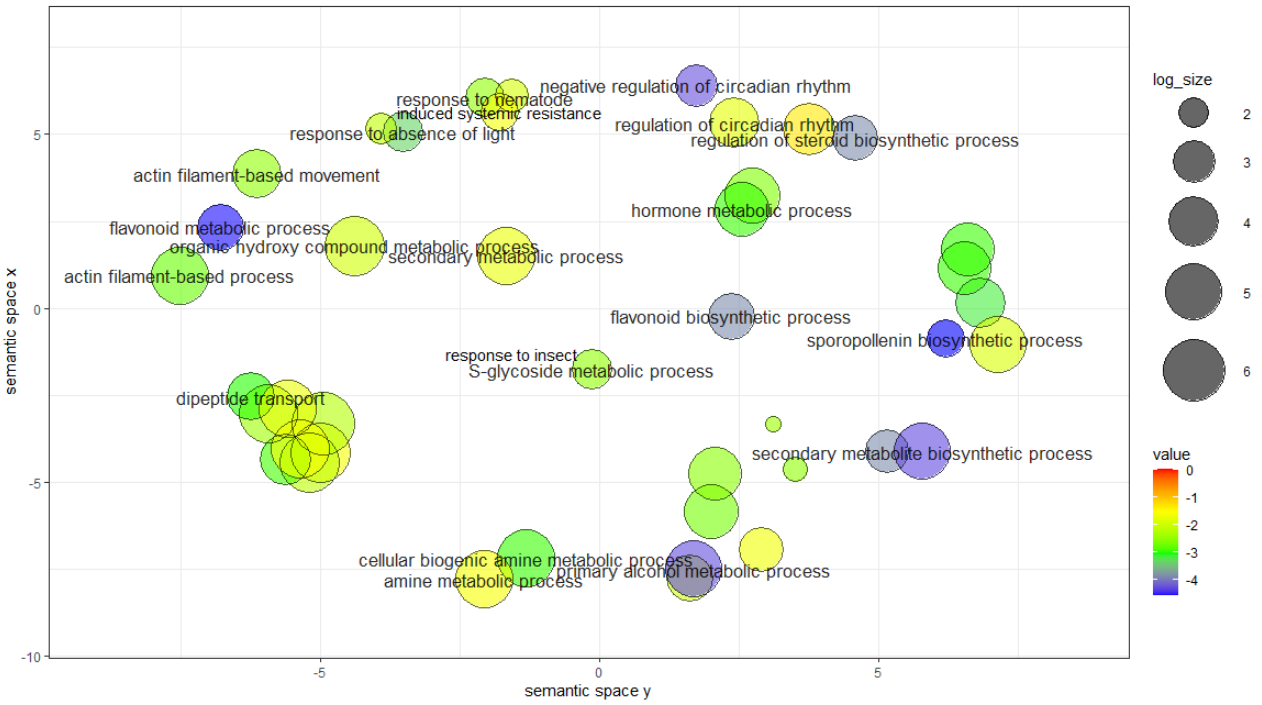

B –

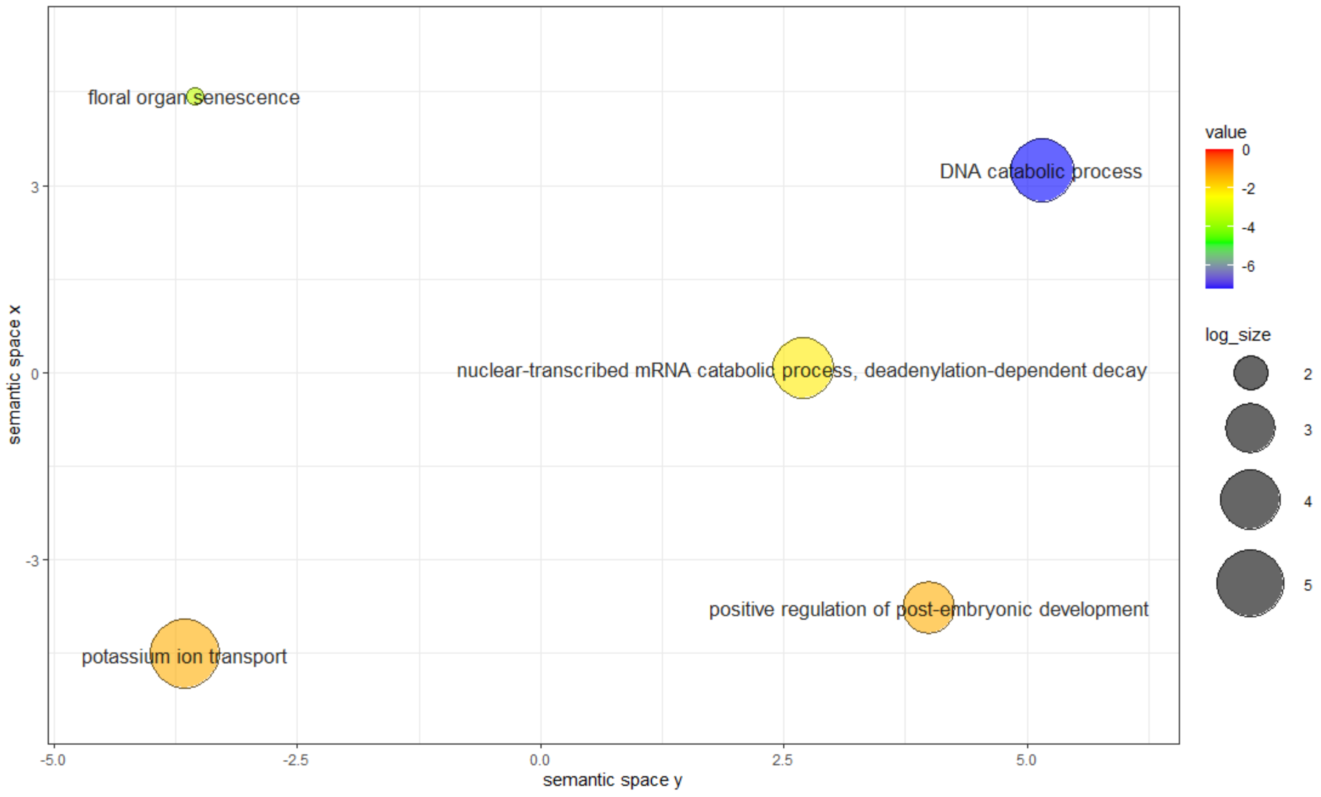

C –

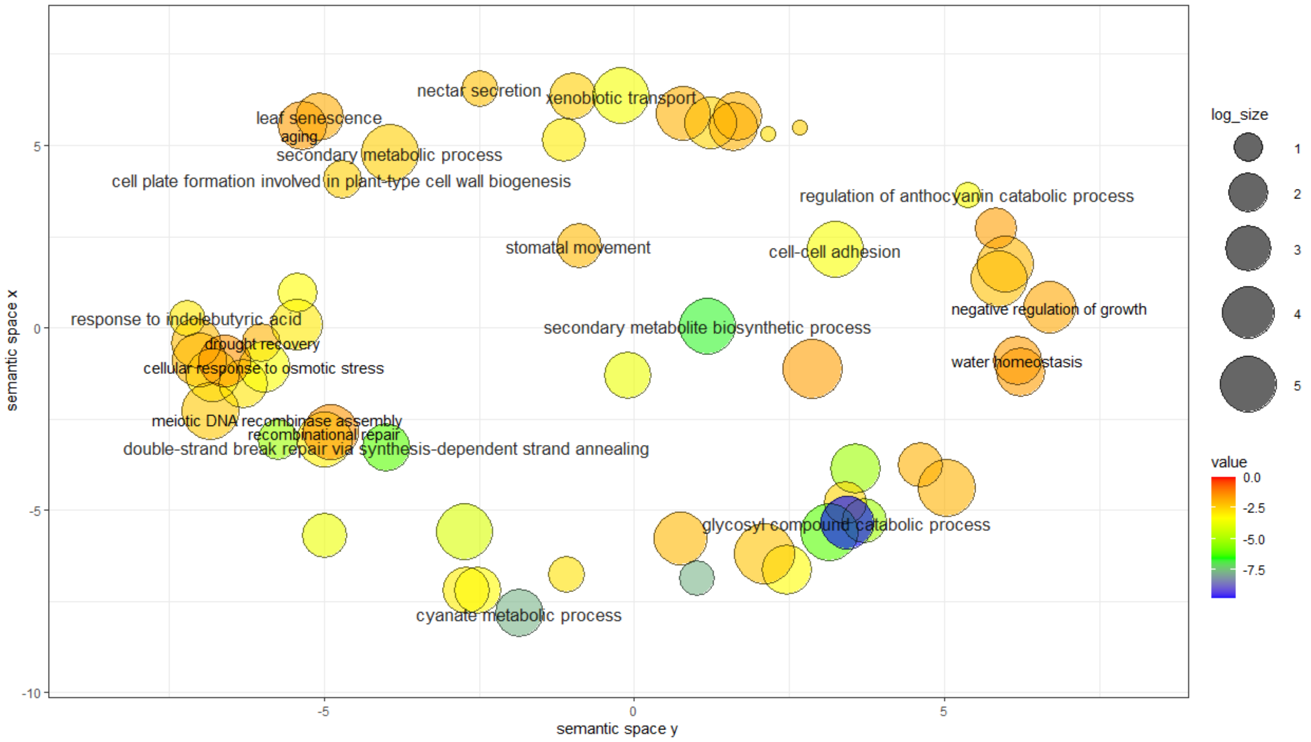

Figure S8 – Enriched terms for BP category for triads that include TE insertions from different superfamily and were assigned to the suppressed relative expression category.
A-DTM, B-RLX, C-XXX. The scatterplot was generated by REVIGO , bubble color indicates the FDR value and the size indicates the frequency of the GO term in the GOA database, bubbles of more general terms are larger.

A –

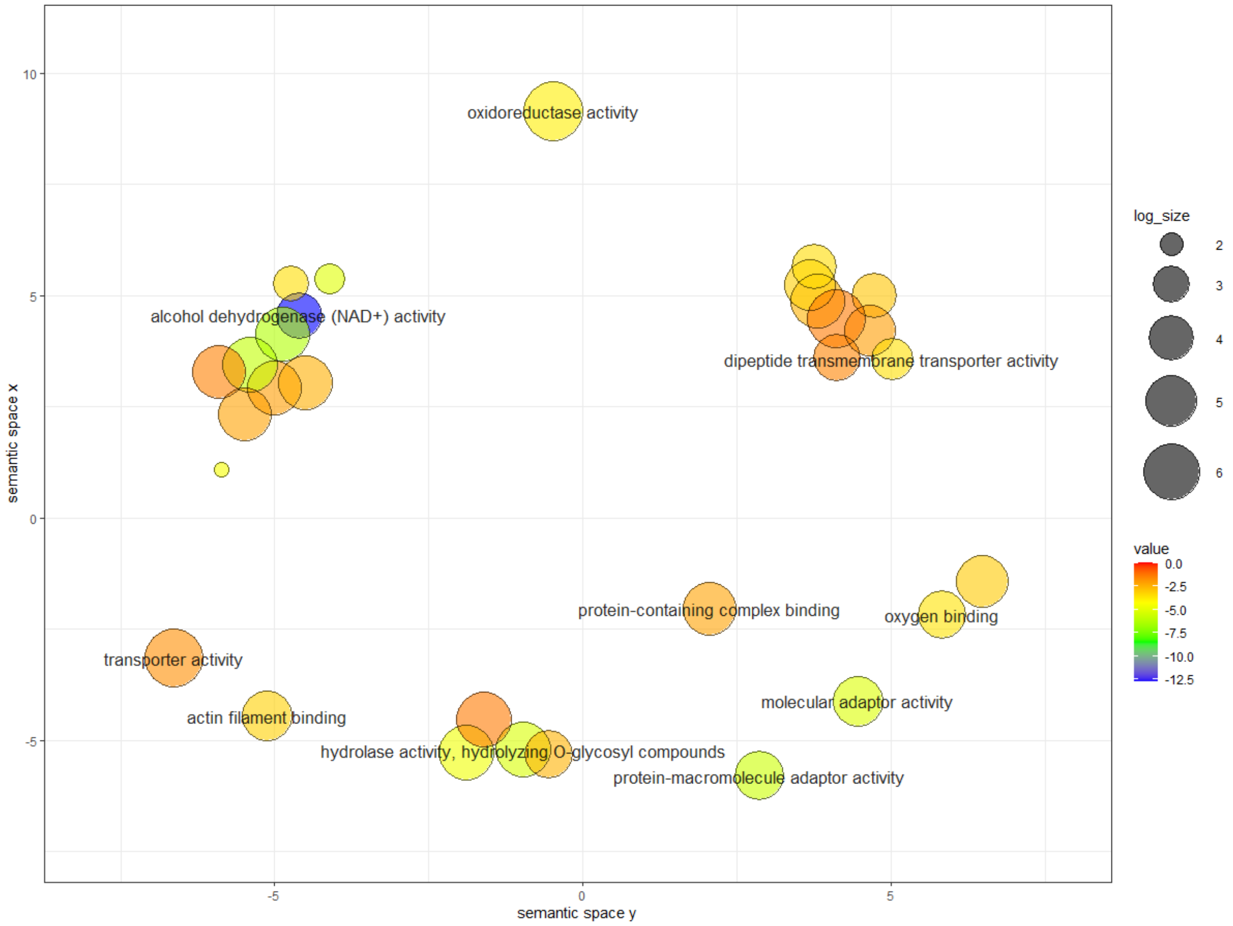

B –

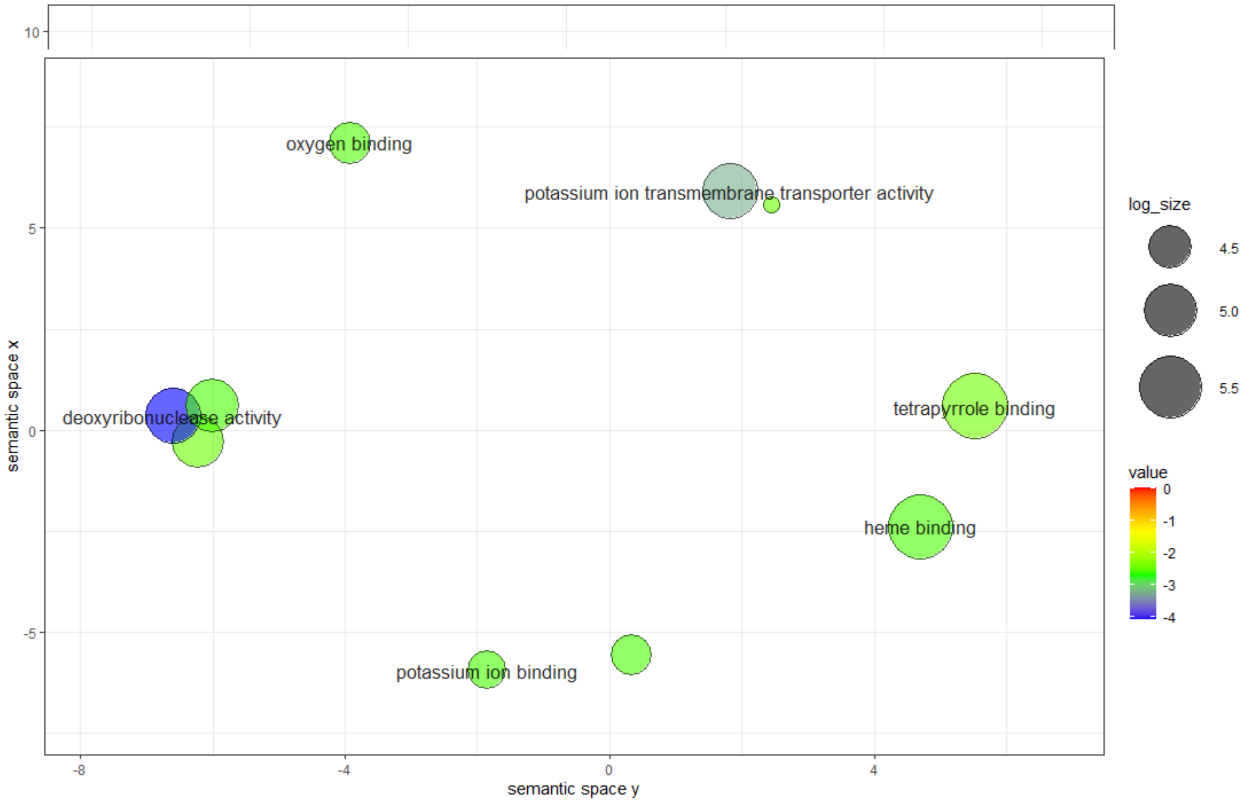
oxygen binding
potassium ion transmembrane transporter activity
deoxyribonuclease activity
tetrapyrrole binding
heme binding
potassium ion binding
semantic space x
semantic space y
log_size
4.5
5.0
5.5
value
0
-1
-2
-3
-4

C –

D –

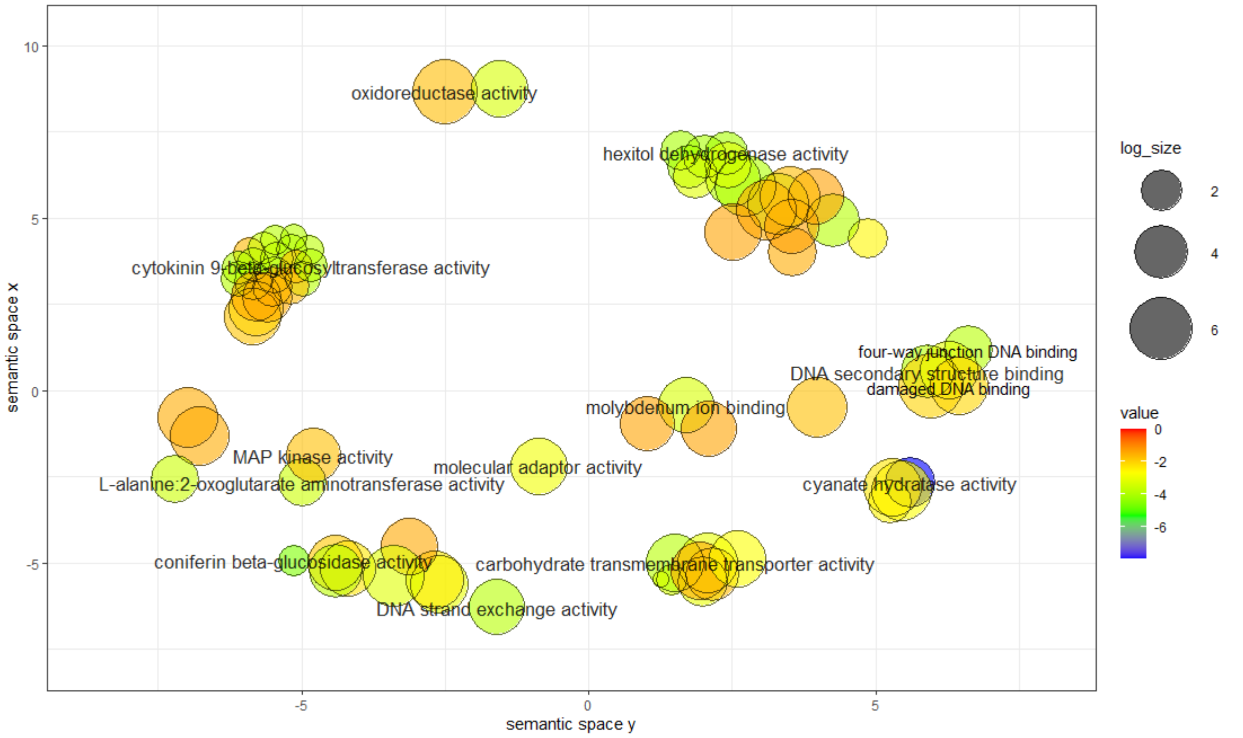
oxidoreductase activity
hexitol dehydrogenase activity
cytokinin 9-beta-glucosyltransferase activity
four-way junction DNA binding
DNA secondary structure binding
damaged DNA binding
molybdenum ion binding
MAP kinase activity
molecular adaptor activity
L-alanine:2-oxoglutarate aminotransferase activity
cyanate hydratase activity
coniferin beta-glucosidase activity
carbohydrate transmembrane transporter activity
DNA strand exchange activity
semantic space x
semantic space y
log_size
2
4
6
value
0
-2
-4
-6

Figure S9 – Enriched terms for MF category for triads that include TE insertions from different superfamily and were assigned to the suppressed relative expression category. A-DTM, B-RLC, C-RLX, D-XXX. The scatterplot was generated by REVIGO , bubble color indicates the FDR value and the size indicates the frequency of the GO term in the GOA database, bubbles of more general terms are larger.

A –

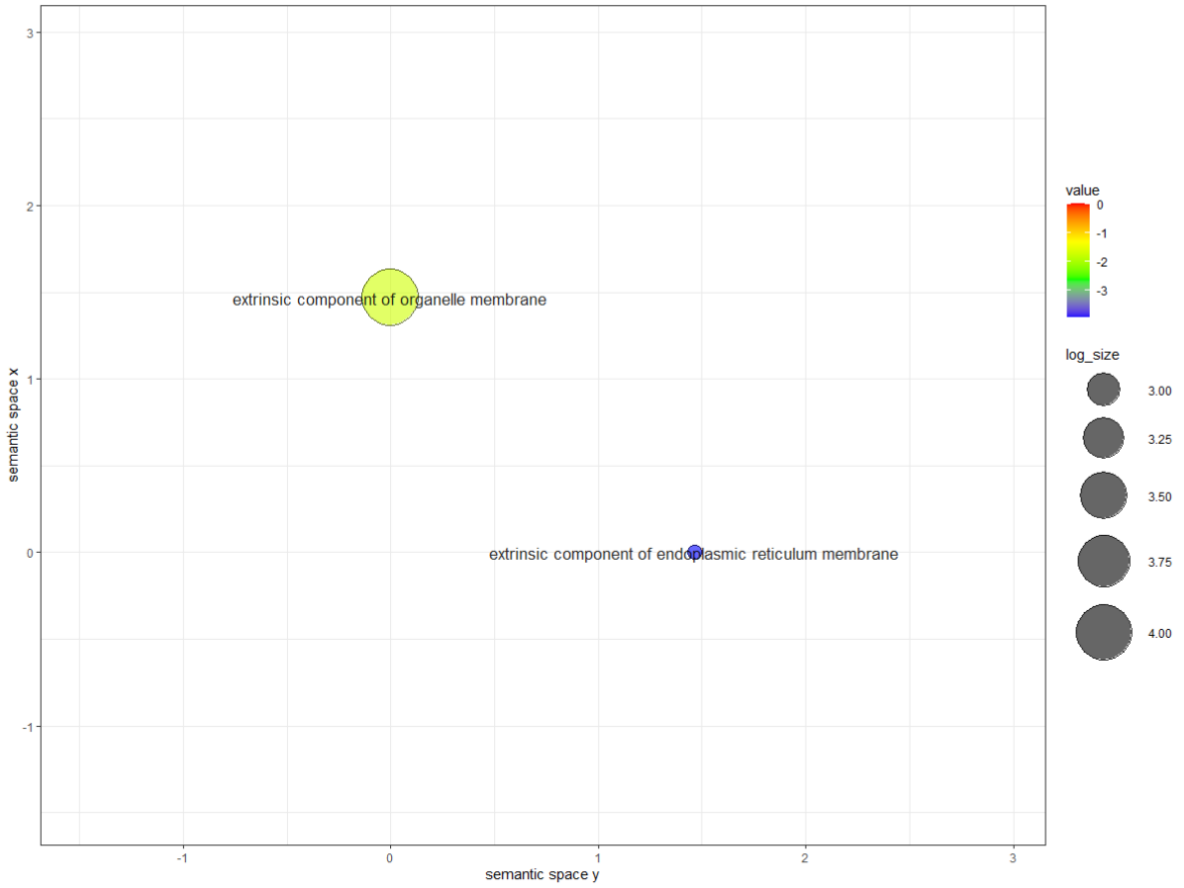

B –

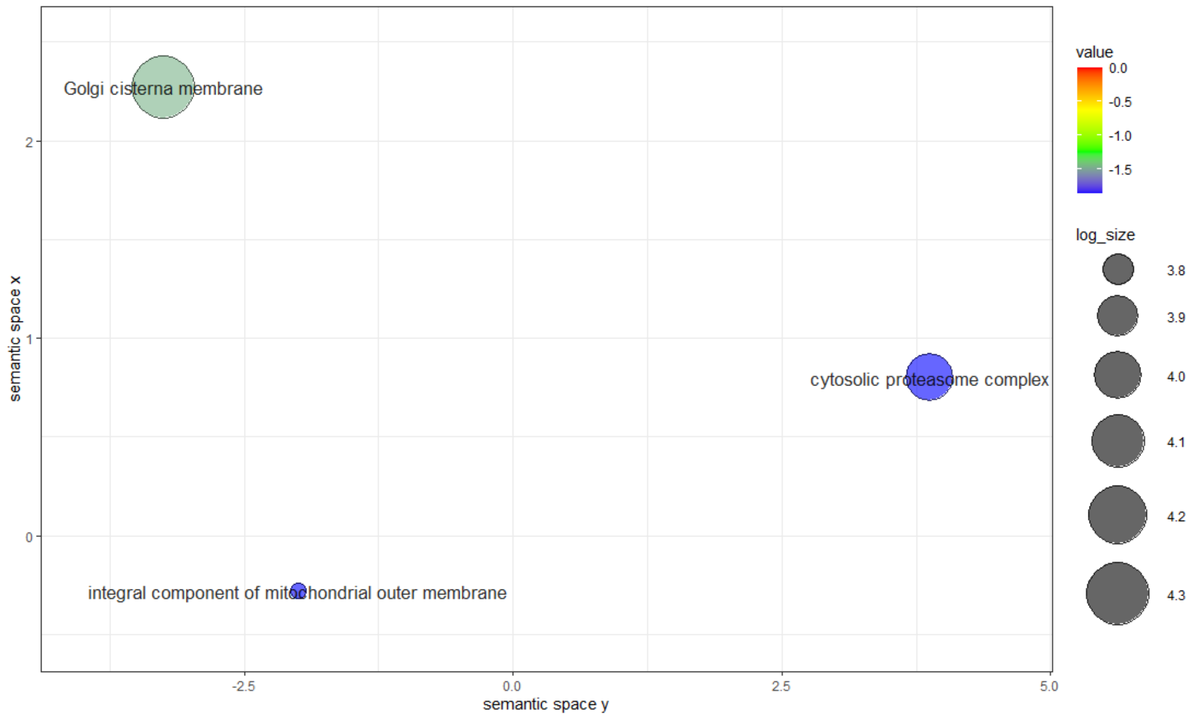

C –

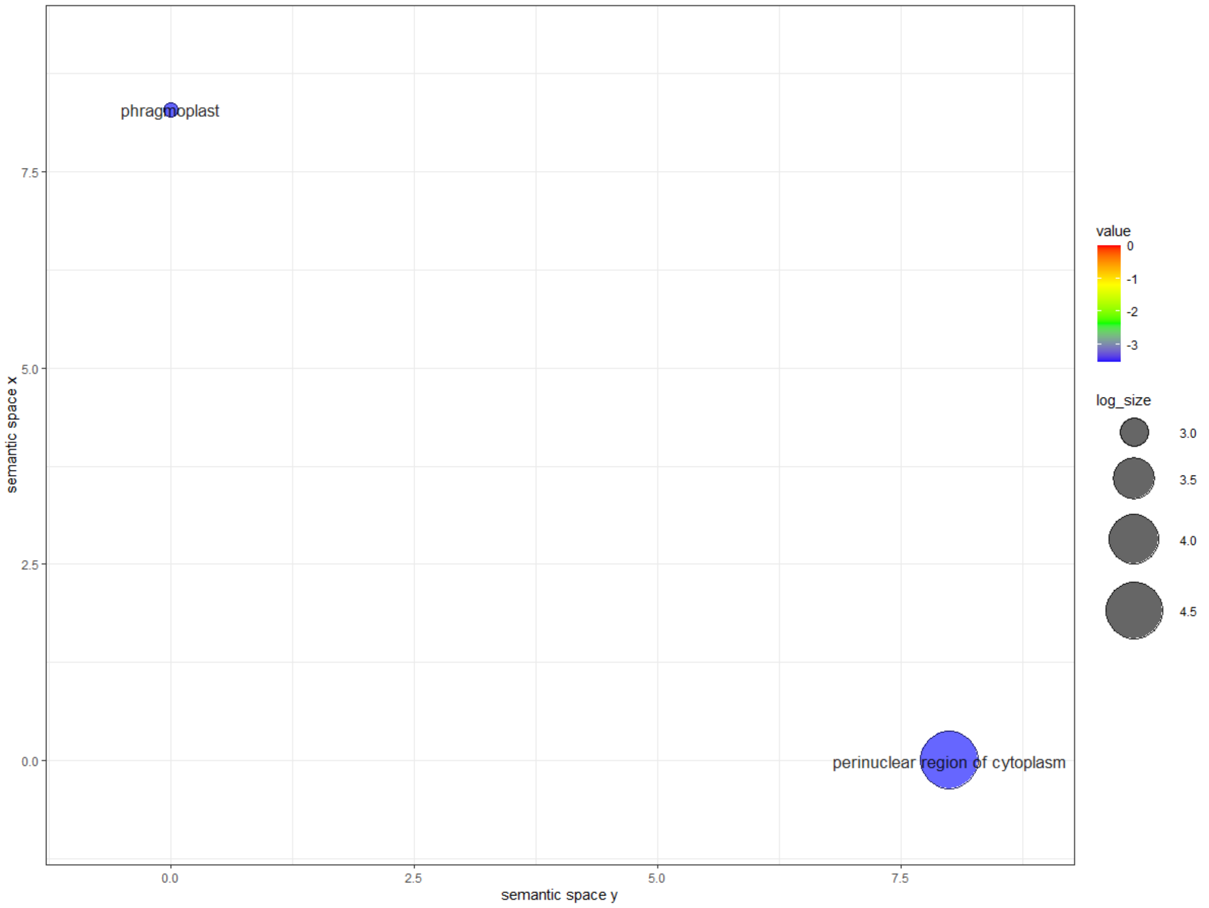

Figure S10 – Enriched terms for CC category for triads that include TE insertions from different superfamily and were assigned to the suppressed relative expression category. A-DTM, B-RLC, C-XXX. The scatterplot was generated by REVIGO , bubble color indicates the FDR value and the size indicates the frequency of the GO term in the GOA database, bubbles of more general terms are larger.

A –

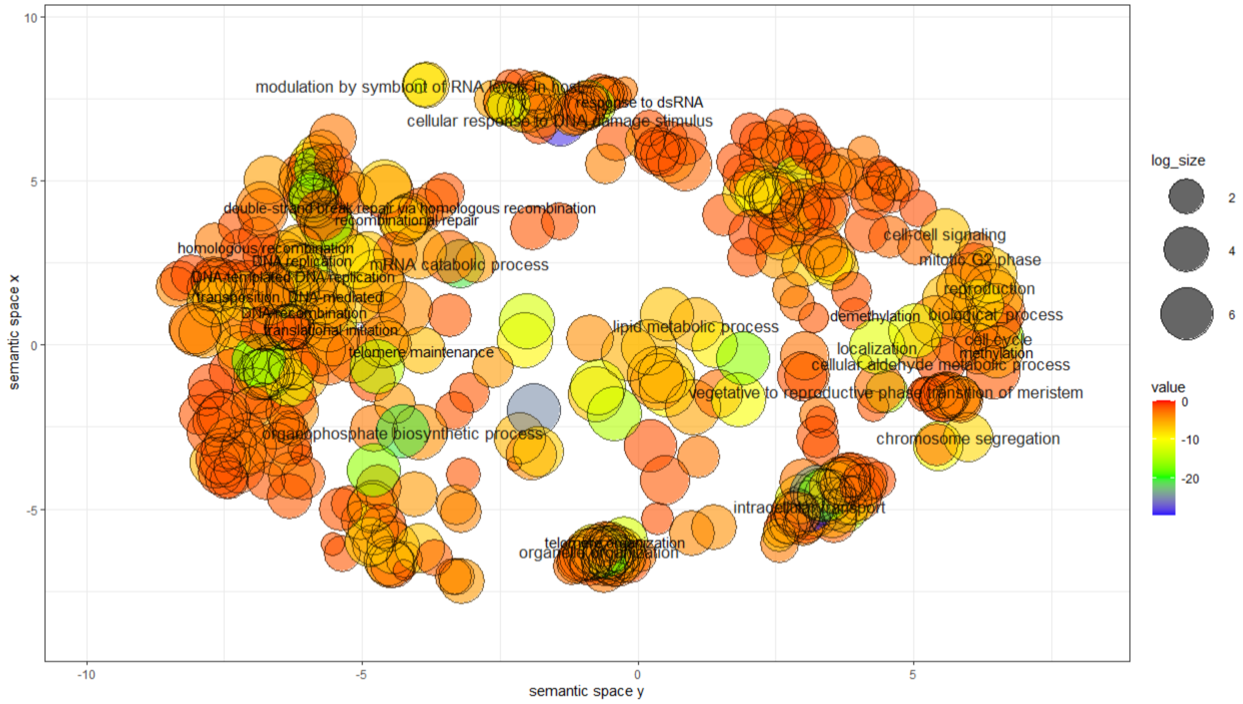

B –

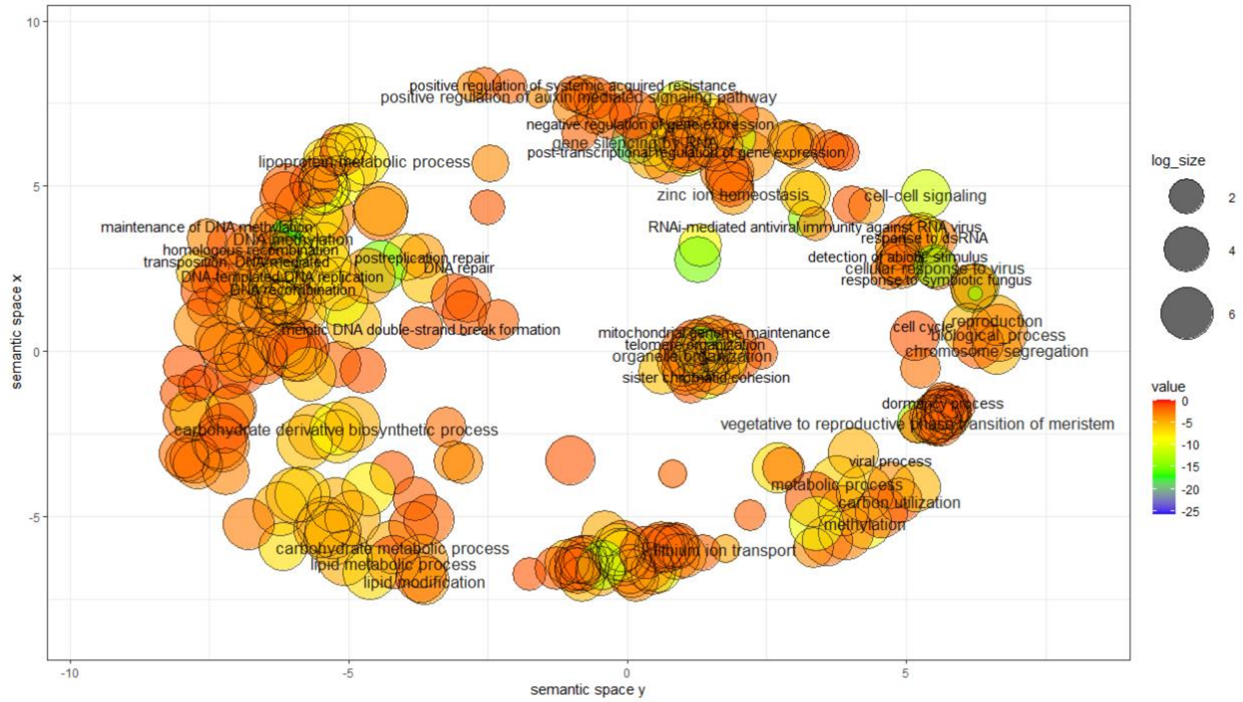

Figure S11 – Enriched terms for BP category for triads that include TE insertions from different superfamily and were assigned to the suppressed relative expression category. A-DTT, B-DTX. The scatterplot was generated by REVIGO , bubble color indicates the FDR value and the size indicates the frequency of the GO term in the GOA database, bubbles of more general terms are larger.

A –

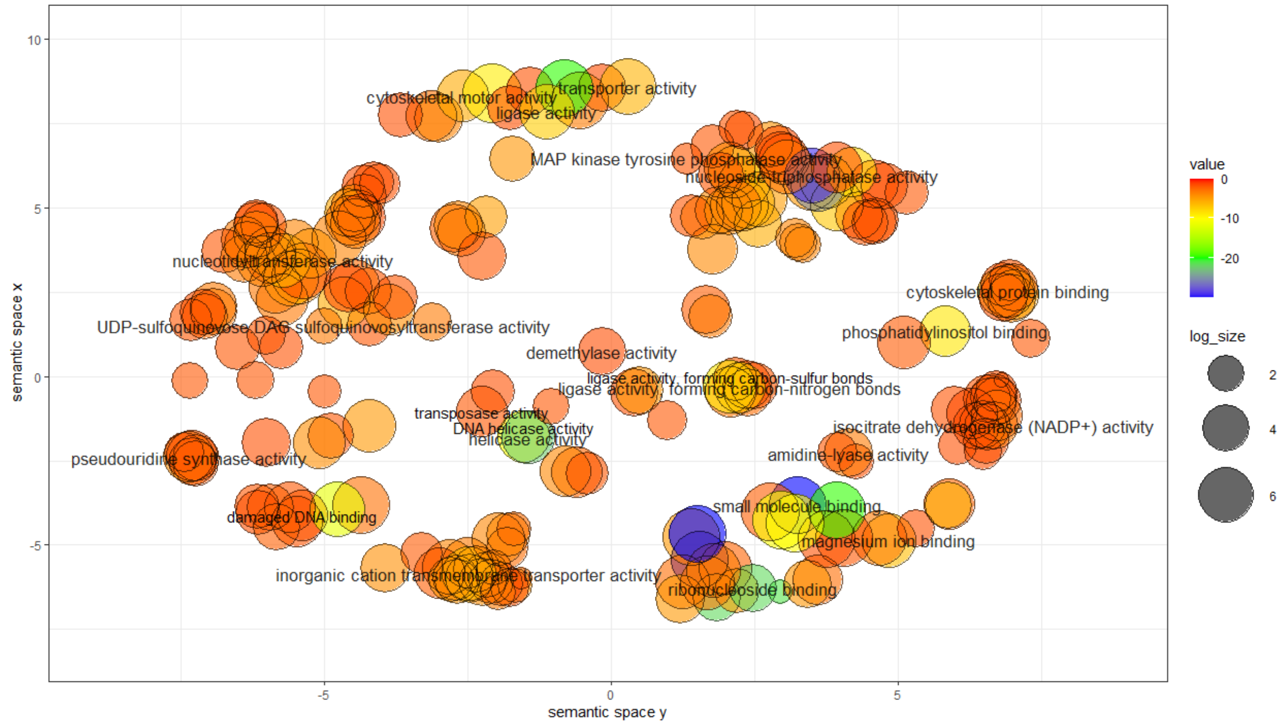

B –

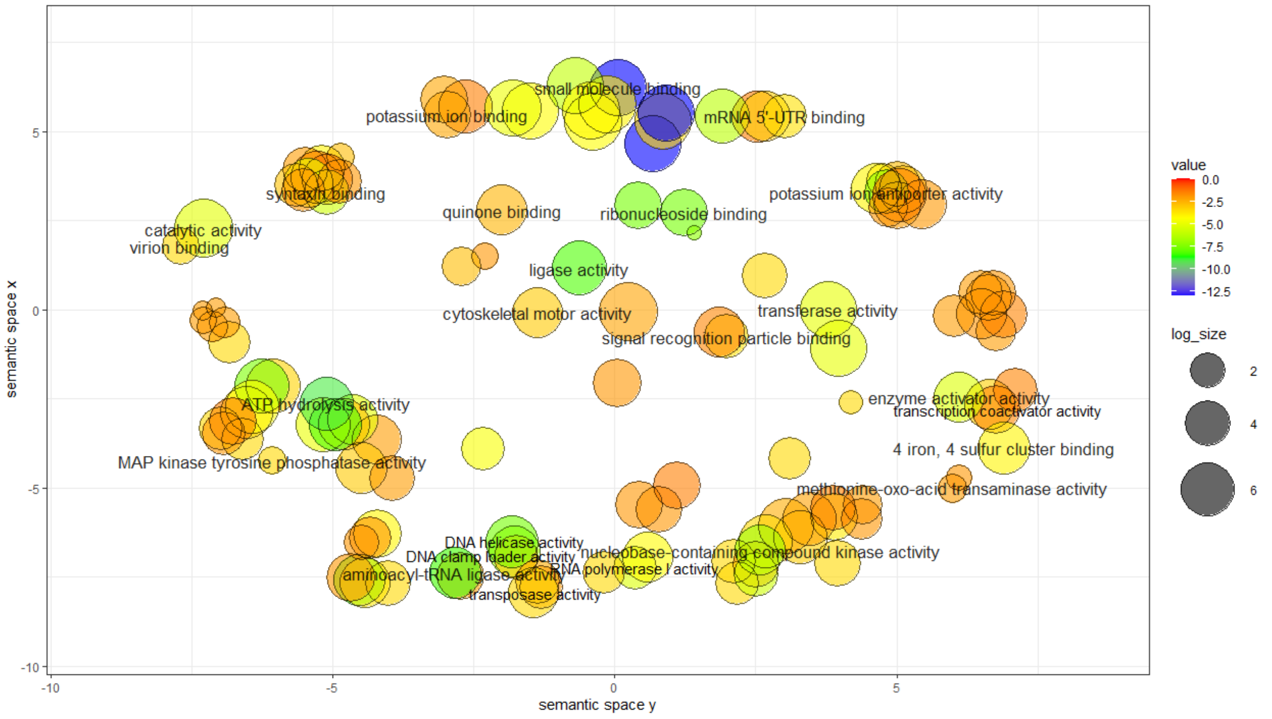

Figure S12 – Enriched terms for MF category for triads that include TE insertions from different superfamily and were assigned to the suppressed relative expression category. A-DTT, B-DTX. The scatterplot was generated by REVIGO , bubble color indicates the FDR value and the size indicates the frequency of the GO term in the GOA database, bubbles of more general terms are larger.

A –

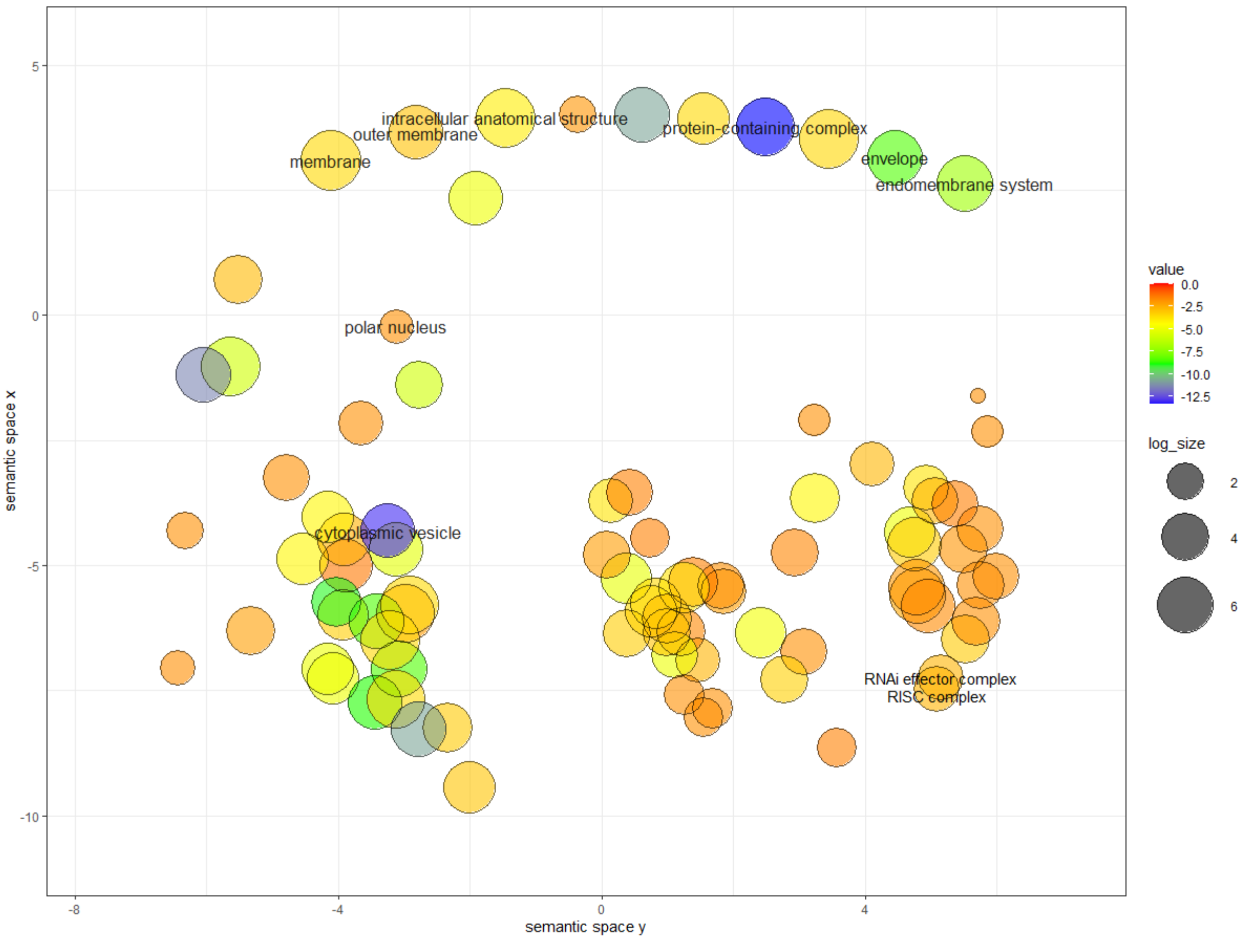

B –

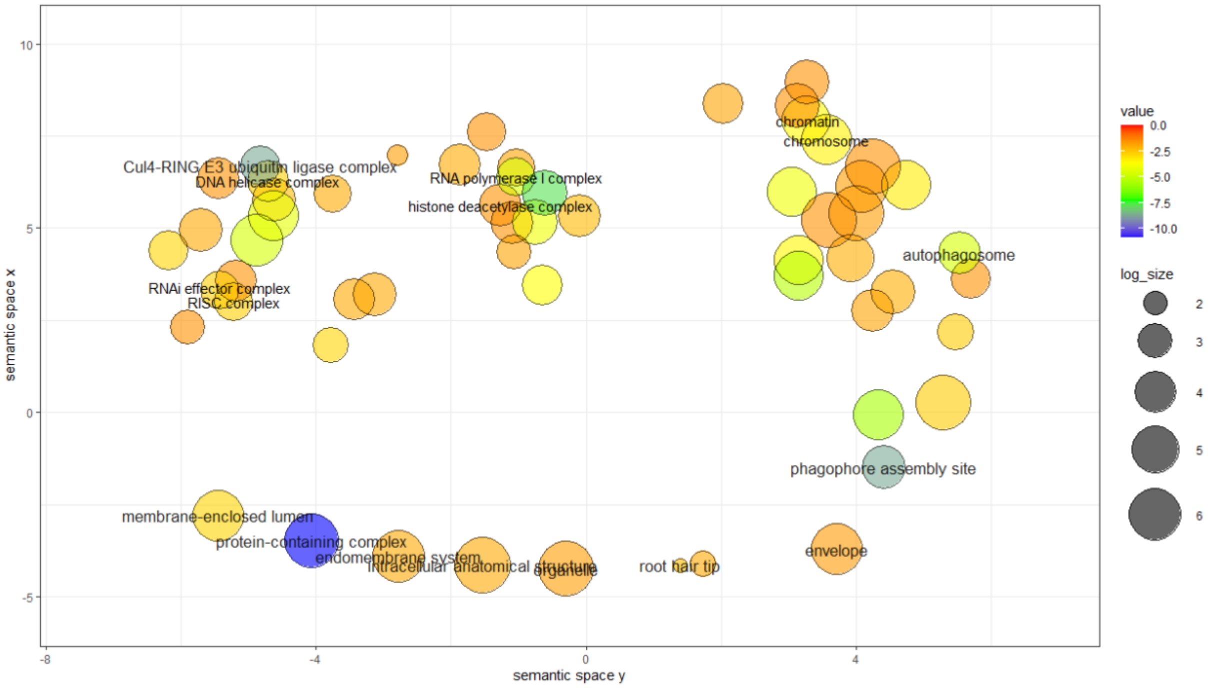

Figure S13 – Enriched terms for CC category for triads that include TE insertions from different superfamily and were assigned to the suppressed relative expression category. A-DTT, B-DTX. The scatterplot was generated by REVIGO , bubble color indicates the FDR value and the size indicates the frequency of the GO term in the GOA database, bubbles of more general terms are larger.
